# A protein coevolution method uncovers critical features of the Hepatitis C Virus fusion mechanism

**DOI:** 10.1371/journal.ppat.1006908

**Published:** 2018-03-05

**Authors:** Florian Douam, Floriane Fusil, Margot Enguehard, Linda Dib, Francesca Nadalin, Loïc Schwaller, Gabriela Hrebikova, Jimmy Mancip, Laurent Mailly, Roland Montserret, Qiang Ding, Carine Maisse, Emilie Carlot, Ke Xu, Els Verhoeyen, Thomas F. Baumert, Alexander Ploss, Alessandra Carbone, François-Loïc Cosset, Dimitri Lavillette

**Affiliations:** 1 CIRI–International Center for Infectiology Research, Team EVIR, Inserm, U1111, Université Claude Bernard Lyon 1, CNRS, UMR5308, Ecole Normale Supérieure de Lyon, Univ Lyon, Lyon, France; 2 CNRS UMR5557 Microbial ecology, Université Claude Bernard Lyon 1, INRA, UMR1418, Villeurbanne, France; 3 Department of Molecular Biology, Princeton University, Princeton NJ, United States of America; 4 University of Lyon, Université Claude Bernard Lyon1, INRA, EPHE, IVPC, Viral Infections and Comparative Pathology, UMR754, Lyon, France; 5 Institut Hospitalo-Universitaire, Pôle Hépato-digestif, Hôpitaux Universitaires de Strasbourg, Strasbourg, France; 6 Molecular Phylogenetics and Speciation, Département d’écologie et évolution, Université de Lausanne, Lausanne, Suisse; 7 Sorbonne Université, CNRS, IBPS, UMR 7238, Laboratoire de Biologie Computationnelle et Quantitative, Paris, France; 8 Mathematical Institute, Leiden University, Leiden, The Netherlands; 9 Inserm, U1110, Institut de Recherche sur les Maladies Virales et Hépatiques, Strasbourg, France; 10 Université de Strasbourg, Strasbourg, France; 11 Institut de Biologie et Chimie des Protéines, Bases Moléculaires et Structurales des Systèmes Infectieux, Labex Ecofect, UMR 5086 CNRS, Université de Lyon, Lyon, France; 12 CAS Key Laboratory of Molecular Virology and Immunology, Unit of interspecies transmission of arboviruses and antivirals, Institut Pasteur of Shanghai, Shanghai Institutes for Biological Sciences, Chinese Academy of Sciences, Shanghai, China; 13 Institut Universitaire de France, Paris, France; NIH, UNITED STATES

## Abstract

Amino-acid coevolution can be referred to mutational compensatory patterns preserving the function of a protein. Viral envelope glycoproteins, which mediate entry of enveloped viruses into their host cells, are shaped by coevolution signals that confer to viruses the plasticity to evade neutralizing antibodies without altering viral entry mechanisms. The functions and structures of the two envelope glycoproteins of the Hepatitis C Virus (HCV), E1 and E2, are poorly described. Especially, how these two proteins mediate the HCV fusion process between the viral and the cell membrane remains elusive. Here, as a proof of concept, we aimed to take advantage of an original coevolution method recently developed to shed light on the HCV fusion mechanism. When first applied to the well-characterized Dengue Virus (DENV) envelope glycoproteins, coevolution analysis was able to predict important structural features and rearrangements of these viral protein complexes. When applied to HCV E1E2, computational coevolution analysis predicted that E1 and E2 refold interdependently during fusion through rearrangements of the E2 Back Layer (BL). Consistently, a soluble BL-derived polypeptide inhibited HCV infection of hepatoma cell lines, primary human hepatocytes and humanized liver mice. We showed that this polypeptide specifically inhibited HCV fusogenic rearrangements, hence supporting the critical role of this domain during HCV fusion. By combining coevolution analysis and *in vitro* assays, we also uncovered functionally-significant coevolving signals between E1 and E2 BL/Stem regions that govern HCV fusion, demonstrating the accuracy of our coevolution predictions. Altogether, our work shed light on important structural features of the HCV fusion mechanism and contributes to advance our functional understanding of this process. This study also provides an important proof of concept that coevolution can be employed to explore viral protein mediated-processes, and can guide the development of innovative translational strategies against challenging human-tropic viruses.

## Author summary

Several virus-mediated molecular processes remain poorly described, which dampen the development of potent anti-viral therapies. Hence, new experimental strategies need to be undertaken to improve and accelerate our understanding of these processes. Here, as a proof of concept, we employ amino-acid coevolution as a tool to gain insights into the structural rearrangements of Hepatitis C Virus (HCV) envelope glycoproteins E1 and E2 during virus fusion with the cell membrane, and provide a basis for the inhibition of this process. Our coevolution analysis predicted that a specific domain of E2, the Back Layer (BL) is involved into significant conformational changes with E1 during the fusion of the HCV membrane with the cellular membrane. Consistently, a recombinant, soluble form of the BL was able to inhibit E1E2 fusogenic rearrangements and HCV infection. Moreover, predicted coevolution networks involving E1 and BL residues, as well as E1 and BL-adjacent residues, were found to modulate virus fusion. Our data shows that coevolution analysis is a powerful and underused approach that can provide significant insights into the functions and structural rearrangements of viral proteins. Importantly, this approach can also provide structural and molecular basis for the design of effective anti-viral drugs, and opens new perspectives to rapidly identify effective antiviral strategies against emerging and re-emerging viral pathogens.

## Introduction

*Flaviviridae* such as Hepatitis C Virus (HCV), Dengue Virus (DENV), Zika Virus (ZIKV) or West Nile Virus (WNV) are cause of several acute and chronic diseases worldwide. The continuous investigation of the molecular processes by which these RNA viruses infect and replicate into their host is critical to develop innovative anti-viral strategies and anticipate viral resistance to pre-existing drugs.

The high potency of viral genomes to mutate is often considered as a major limitation for the development of effective anti-viral strategies. Nevertheless, the high-mutation rate of RNA viruses represents a unique opportunity to decrypt viral protein functions and structures. Highly-evolving viral genomes are shaped by important evolutionary constraints to maintain genetic structure and proper protein folding. Amino-acid coevolution, which refers to mutations of different residues at a similar time frame, mirrors such constraints. Hence, the identification and characterization of coevolution signals imprinted within viral protein sequences can provide unique insights into viral protein functions and conformational changes, and ultimately guide the design of original anti-viral strategies.

Virus entry is a conserved, critical step during the viral life cycle and represents a valuable target for the development of antivirals and vaccines. The entry process of HCV is orchestrated by two envelope glycoproteins, E1 and E2, which are incorporated onto the virion surface. During entry, E1E2 mediate viral particle attachment to cell surface receptors and induce the merging (called fusion) of endosomal and virus membranes at acidic pH, thus leading to release of viral RNA into the cytosol [1]. Flaviviruses such as DENV, ZIKV or WNV harbor two envelope glycoproteins: E and PrM. E is a class-II fusion protein composed of three distinct domains (domain I, II and III; or DI, DII and DIII respectively) and carries both binding and membrane fusion properties [2]. Crystal structure of E at different pH allowed to draw a fusion model during which initial E dimers change conformation and fold back as trimer structures to induce membrane merging [3]. In contrast, how HCV E1 and E2 mediate membrane fusion remains poorly defined. Our understanding of the HCV fusion process is strongly dampened by the absence of a well-defined pre- and post-fusion full-length E1E2 crystal structure. Few studies have attempted to computationally model pre-fusion E1E2 complex [4,5] but their impact remain limited as they have to rely on partial structural and functional information that are often collected in a non-heterodimer context.

The structure of a large region of the E2 ectodomain (E2 core) [6,7] exhibits a globular, non-extended fold divided into two distinct sheets: a front sheet composed of a front layer and a central Ig-fold domain, and a back sheet (or back layer, BL). Although the central Ig-fold domain represents a common structure among class-II fusion proteins, the BL harbors an original structure, thus undermining the possibility that HCV E2 is a classical fusion protein. It has been suggested through the resolution of the bovine viral diarrhea pestivirus (BVDV-1) E2 glycoprotein structure that HCV E1 may represent the HCV fusion protein [8–10]. Several studies have identified a hydrophobic region in E1 (CSALYVGDLC) that could represent the putative HCV fusion peptide [11–16]. Another study also suggested that E1 proteins form trimeric structure at the virus surface [17]. However, the recent crystal structure of the N-terminal domain of HCV E1 ectodomain does not harbor the expected truncated class-II fusion protein fold [18], suggesting that HCV fusion might be a unique process.

Mutagenesis studies have shown that both E1 and E2 domains, as well as E1-E2 dialogs, are involved in the HCV fusion process [13,14,19–21]. Thus, rather than being mediated by a single glycoprotein, HCV fusion appears to be mediated by complex intra- and inter-molecular E1-E2 dialogs that shape structural and conformational rearrangements of the heterodimer complex. Consequently, the characterization of interplays between E1 and E2 is critical to decipher the HCV fusion mechanism.

Here, using HCV fusion as a model of study, we aimed to provide a proof of concept that amino-acid coevolution and protein evolutionary constraints can shed light on viral protein functions and rearrangements. We hypothesized that detection of E1-E2 coevolution patterns can uncover their functional interplays as well as critical features of the HCV fusion mechanism.

We recently developed an original computational method, **B**locks **I**n **S**equences (BIS), that can robustly detect coevolution signals within conserved cellular and viral proteins using a limited number of protein sequences [22–24]. Taking advantage of this methodology, we aim at establishing a map of E1-E2 coevolution patterns and test whether coevolution analysis can be employed to gain mechanistic insight into poorly characterized viral processes such as HCV fusion. BIS was able to accurately predict features of DENV glycoproteins structural organization onto viral particle, as well as E fusogenic conformational changes. When applied to HCV E1E2, BIS suggested that HCV E2 BL is a critical modulator of HCV fusion. Consistently, a soluble form of the E2 BL was able to inhibit HCV fusion. Moreover, coevolution signals between E1 and E2 BL/Stem predicted by BIS were found to regulate virus fusion *in vitro*. Beyond providing novel insights into the HCV fusion mechanism, our work also demonstrates that coevolution analysis can shed light on viral-mediated processes and can open avenues for the accelerated design of innovative anti-viral compounds against challenging human tropic-viruses.

## Results

### A computational coevolution-based method recapitulates DENV glycoproteins structural organization

**Fig 1:**
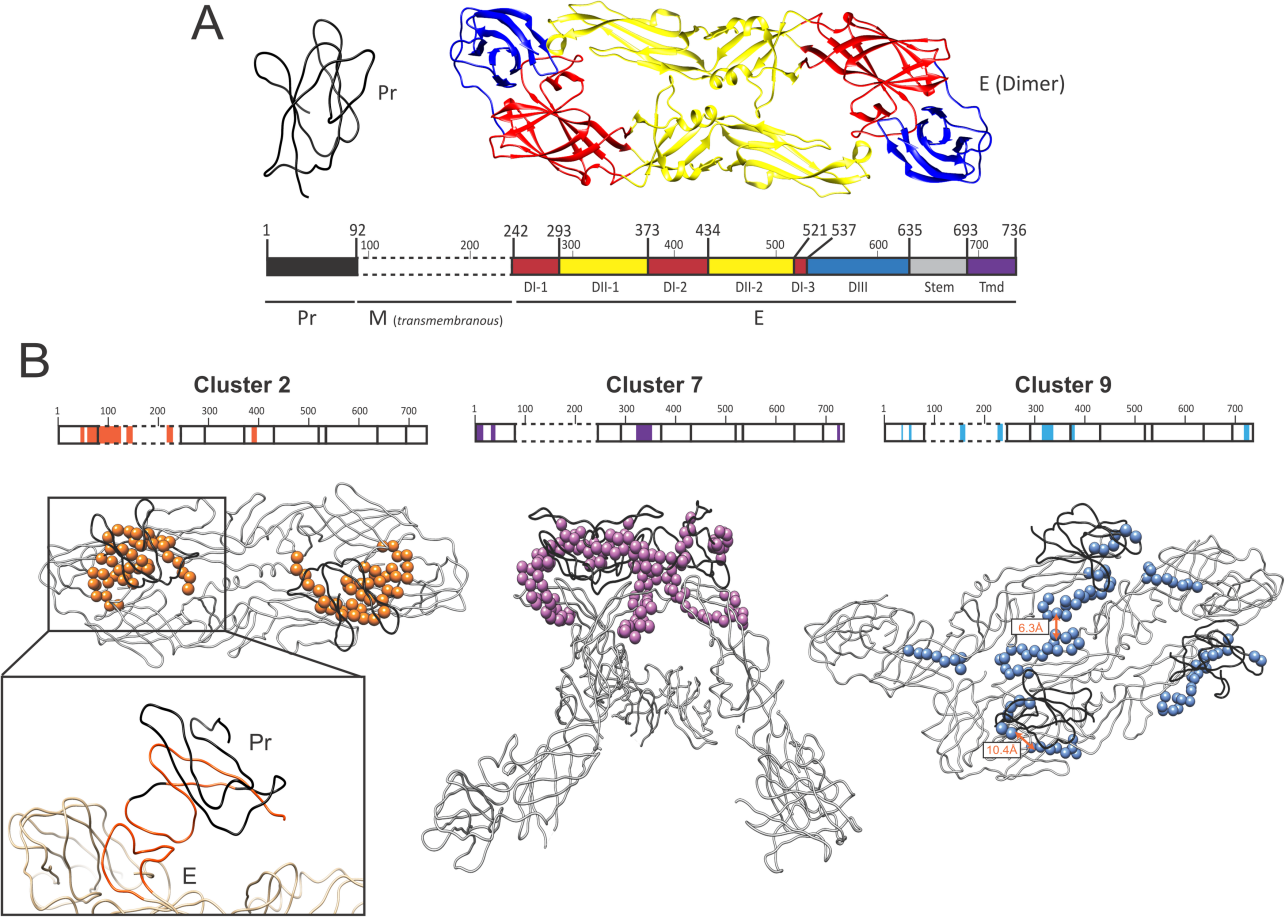
BIS analysis of dengue E-Pr coevolving residues. (**A**) Tridimensional representation of DENV Pr (Black, PDB 3C6R) and E (multi-color, PDB 1K4R). A linear representation of the PrM-E polyprotein is depicted below the protein structures. Starting and ending residue positions of each protein (Pr, M and E) and E domain are indicated. E domains are annotated by distinct colors: DI, domain I (red); DII, domain II (yellow); DIII, domain III (blue); Tmd, transmembrane (black). (**B**) Organization and positions of the PrM-E cluster 2 (orange), 7 (blue) and 9 (pink) on tridimensional representations of the DENV E and Pr proteins. Cluster 2 and 9 are depicted on a dimeric or trimeric E-Pr structure respectively at low pH condition (PDB 3C6R). Cluster 7 is depicted on a trimeric E-Pr structure at neutral pH condition (PDB 3XIY). Linear representation of the two proteins are also depicted on the top of each structure and cluster block location are indicated (precise cluster positions are reported in **S1 Table**). The close proximity between DENV E and Pr cluster 2 blocks is enlarged.

We have previously reported that BIS, a combinatorial-based coevolution analysis method (**S1 Fig**), can accurately detect coevolution signals within a wide range of well-characterized cellular and viral proteins [22–24]. As BIS has not been previously tested for its ability to predict viral envelope glycoproteins structural organization and rearrangements, we first performed a coevolution analysis of the well-characterized DENV envelope glycoproteins, E and PrM [2,3]. During virus maturation, M protein (a mainly transmembranous protein) is associated to Pr, a peptide that protect the E fusion peptide and is cleaved prior viral budding [25]. Briefly, the PrM-E complex protrude as trimer at the surface of immature viral particles in the endoplasmic reticulum, a neutral pH compartment. Immature particle then navigates toward the trans-Golgi network, a more acidic compartment, where PrM-E complex form dimeric structures that lie down onto the surface of the particles. Pr, which initially concealed E fusion peptide, is then cleaved by the Furin. This cleavage achieves the maturation of viral particles that are then released into the extracellular compartment. As M is mostly a transmembrane protein with only very partial structural information available, we aimed to determine whether BIS can recapitulate the diversity of E-Pr structural organization at the surface of immature viral particles. BIS analysis of 17 DENV PrM-E serotype 2 sequences led to the identification of 14 groups of coevolving residues (possibly organized in blocks of consecutive amino-acids), further referred as clusters (**S1 Table**). Among those, three clusters (cluster 2, 7 and 9) displayed a strong statistical significance (with associated p values of 8e-5, 8e-5 and 7e-3 respectively) and involved coevolving blocks between E and Pr. When E is assembled as dimer at low pH condition, cluster 2 coevolving block positions supported the close proximity of E DIII and Pr (**Fig 1A and 1B-left**). Similarly, cluster 7 also recapitulated the close proximity between Pr and E domain DII on a trimeric E-Pr structure that form at neutral pH in the endoplasmic reticulum (**Fig 1A and 1B-center**). On a trimeric Pr-E structure (one dimer + one monomer of PrM-E) found at low pH when particles mature in the trans-Golgi, cluster 9 also supported a close proximity between Pr and E DII, but also between E dimers (**Fig 1A and 1B-right**). Two other clusters (cluster 3 and 8) of strong statistical significance (with respective p values of 1.34e-5 and 4.11e-5) were identified by BIS but did not involve coevolving residues located within E protein (**S1 Table**). Although cluster 8 was composed of two coevolving blocks located within M protein only, clusters 3 involved coevolving residues located within both M and Pr. As cluster 3, cluster 2 and 9 also supported the existence of interactions between Pr and M (**Fig 1A and 1B**). As M and Pr are the two cleavage products of a single PrM protein, these three clusters hence represent additional evidence of BIS ability to recapitulate biologically significant protein interactions. Taken together, our results demonstrate that BIS has the ability to accurately predict the tridimensional assembly of two viral proteins within different conformational states.

### BIS can predict DENV E fusogenic conformational changes

**Fig 2:**
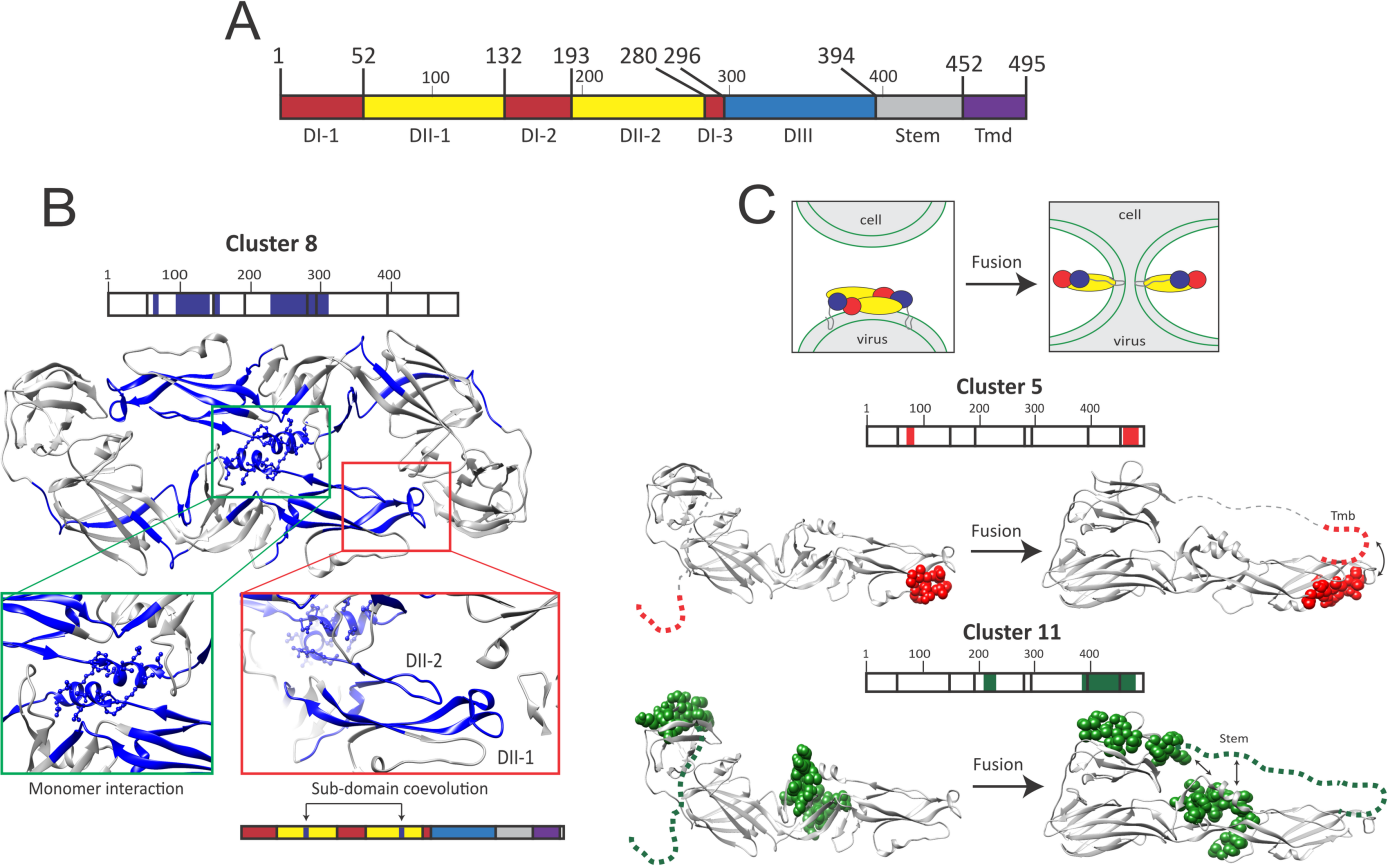
BIS analysis of dengue E coevolving networks during pre- and post-fusion steps. (**A**) Linear representation of Dengue E protein. Starting and ending residue positions of each E domain are indicated. E domains are annotated by distinct colors: DI, domain I (red); DII, domain II (yellow); DIII, domain III (blue); Tmd, transmembrane (black). (**B**) Organization and positions of the DENV E cluster 8 blocks (dark blue) on a mature E dimeric structure (PDB 1K4R). A linear representation of E is depicted and cluster block locations are indicated (precise cluster positions are reported in **S2 Table**). Cluster 8 coevolving residues located in areas where the two E monomers are in close contact are enlarged (green square). Structural proximity of two-coevolving cluster 8 internal loops located into two distinct Domain II (DII) sub-domains on the linear structure is also highlighted (red square). (**C**) Positions of E cluster 5 (red) and 10 (green) blocks on a mature tridimensional E monomer at a pre-fusion state (left, PDB 1K4R) and post-fusion state (right, PDB 1OK8). Stem and transmembrane domains are represented by a grey dotted line. A linear representation of E and cluster block locations are indicated for each cluster (precise cluster positions are reported in **S2 Table**). At the top of the panel, a schematic represents the current experimentally-validated fusion model of DENV, and how DENV E rearranges during this process. DENV E domains are colored into distinct colors (red, domain I; yellow, domain II; blue, domain III) as in **Fig 1A**.

As DENV E has been demonstrated to mediate DENV viral fusion, we then aimed to study the intra-protein coevolution signals within DENV E only (**Fig 2A;** see new E numbering by BIS in comparison to **Fig 1A**), and determined whether coevolution signals can also predict E fusogenic rearrangements. Using 17 different DENV E serotype 2 sequences, BIS identified 12 clusters (**S2 Table**). Among them, nine clusters (clusters 2–8 and 11,12) displayed associated p-values ranging between 7e-3 to 2e-7 and two clusters (clusters 9 and 10) exhibited associated p-values of 0.058 (**S2 Table**). Cluster 1 and 2 were either conserved (p-value = 1) or too large respectively to be considered. Several clusters (3,4,6 and 7) displayed small blocks located within a single region of E both in the linear protein sequence and on the tridimensional structure, suggesting that coevolution signals might contribute to the structural organization of secondary protein sub-domains (such as internal loops) (**S2 Fig**). DENV E DI and DII are composed of two or three sub-domains that are distant on the linear protein sequence but form single structured domains in the protein tertiary structure. Cluster 8 blocks were mostly located within the two sub-domains of DII and were consistent with the tridimensional organization of this protein domain (**Fig 2B**). Cluster 8 blocks located within the second sub-domain of DII (DII-2) of each E monomer were in close contact on the dimeric E structure (especially at the level of the DII α-helix), hence suggesting that coevolution signals can predict point of contacts between E monomers once organized as dimer (**Fig 2B**). Despite lower statistical significance (p<0.06), cluster 9 and 10 coevolving blocks also supported E structural organization as these blocks were distant on the linear structure but close on the E dimer structure (**S2 Fig**). Finally, three clusters (5, 11 and 12) displayed coevolving blocks that were both distant on the linear and tridimensional E structure. During fusion, E DIII folds-over toward DII, and DII becomes at close proximity with the E transmembrane domain [3] (**Fig 2C**). Cluster 5 and 11 coevolving blocks organization were consistent with these structural rearrangements (**Fig 2C**), suggesting that BIS can recapitulate viral glycoprotein fusogenic conformational changes.

### HCV E1 and E2 are strong coevolving partners that likely refold interdependently during fusion

**Fig 3:**
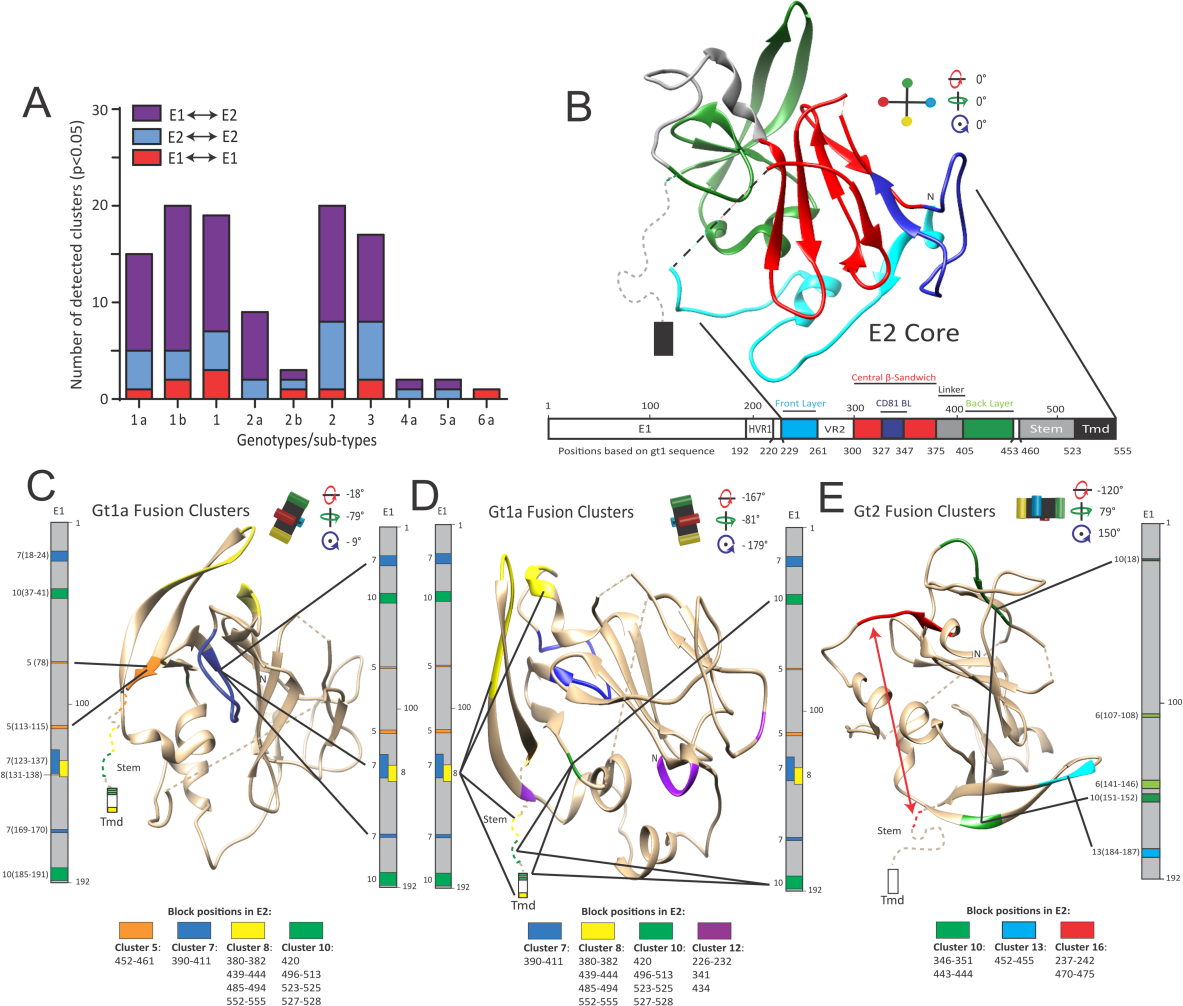
BIS analysis of HCV E1E2 coevolving residues. (**A**) Number of intra-E1 (red), intra-E2 (blue) and inter-E1E2 (purple) clusters detected by BIS following analysis of E1E2 sequences belonging to genotype 1 through 6. (**B**) Linear representation of HCV E1E2 glycoproteins and tridimensional representation of E2core structure (PDB 4MWF). E2 domains and their residue positions are indicated. E2core layers and domains are highlighted by distinct colors (Green, BL; Red, central β-sandwich; Blue, front layer; Dark blue, CD81 BinL/CD81 binding loop; Light grey, central β-sandwich–back layer linker; Black dotted line, VR2/Variable Region 2; Grey dotted line, Stem; Black rectangle, Tmd/Transmembrane). HVR1, Hyper Variable Region 1. Rotation angles of the E2core structure are indicated. Viewing angle of E2core is indicated by a black cross harboring 4 color points at each extremity. (**C-E**) HCV E1E2 gt1a (**C-D**) and gt2 (2a+2b, (**E**)) fusion clusters are plotted on a vertical linear representation of E1 combined to the tridimensional view of E2core (PDB 4MWF). Each cluster is composed of blocks harboring a similar color, according to **S3 Fig** (gt1a) or **S6 Fig** (gt2). Panel **C** shows E1E2 dialogs mediated by gt1a fusion cluster 5 and 7 and panel **D** underlines E1E2 dialogs mediated by gt1a fusion cluster 8, 10 and 12. Panel **E** shows E1E2 dialogs mediated by gt2 fusion cluster 10 and 13. The Stem region (Stem) is represented by a dotted line following the C-terminal part of the BL. The transmembrane domain (Tmd) is represented as a rectangle following the Stem region. Bold lines link E1 and E2 cluster blocks that coevolved. For each cluster, block positions in E1 (at the left of the linear structure) and E2 (below boxes whose color match the color of the corresponding cluster) are indicated. Rotation angles of the E2core structure are indicated and the viewing angle of E2core is indicated by a black cross (as in **Fig 3B**).

Following validation of BIS ability to model viral envelope glycoprotein structural rearrangements, we then applied the BIS methodology to HCV E1E2. We analyzed independently using BIS ten groups of E1E2 sequences from different genotypes (gt) or sub-types (1a, 1b, 1 = 1a+1b, 2a, 2b, 2 = 2a+2b, 3, 4a, 5a and 6a) (**S3 Table**). Interestingly, most of the identified clusters involved residues in both E1 and in E2 suggesting the existence of conserved tight dialogs between E1 and E2 proteins (**Fig 3A**). Only a few number of statistically significant clusters were found among genotype 4a to 6a sequences, due to the low number of sequences available and to their very low genetic divergence.

Unlike Dengue E and PrM, the full panel of HCV E2 conformations still remain unknown and E2core only represent a single of these possible conformations, in a given biochemical context. When BIS coevolution analysis is applied to protein(s) for which only a fraction of its/their conformations have been characterized (which is the case for HCV E1 and E2), BIS coevolution clusters can thus only suggest, but not contradict a given conformational hypothesis, this unless the full panel of a protein conformations is known. Consequently, *in vitro* and/or *in vivo* experiments are then critical to ascertain the functional significance of a given conformational hypothesis.

Given this particular experimental context and in order to identify putative E1E2 rearrangements during HCV fusion, we thus adopted the following approach. First, we aimed to assign to each E1E2 cluster a given function by mapping clusters blocks with residues previously identified in the literature to impact E1E2 folding/heterodimerization, binding or fusion. Second, we sought to identify among BIS clusters classified as “fusion clusters” a putative protein rearrangement supported by several fusion clusters across multiple genotypes, prior experimental challenge through *in vitro* experiments.

We first focused our efforts on analyzing gt1a clusters. Detailed analysis of these clusters (**S4 Table; S3 Fig**; note that BIS numbered E1 and E2 residues by considering the first amino acid of E1 as residue #1) showed E1 as coevolving systematically with all the E2 domains. Plotting the gt1a coevolving blocks onto reference sequences (**S4 Fig**) revealed a strong correlation between blocks and residues previously identified in the literature to be important for heterodimer folding or viral binding site conformation (grouped both under the term “structural”) or fusion. This correlation allowed us to propose functions (structural, fusion, or multifunctional clusters) for most gt1a clusters (**S5 and S6 Tables**). When plotted on the E2core structure (**Fig 3B**), most fusion clusters involved blocks located within the E2 BL (**Fig 3C and 3D)** in contrast to structural or multifunctional clusters for which blocks were broadly distributed across E2 (**S5 Fig**). Interestingly, some of the fusion clusters involved distant blocks in both E1 and E2 (clusters 5,7,10; **Fig 3C and 3D**), highlighting that E1 terminal regions and the E2 BL could be in close proximity during fusogenic rearrangements. In addition, BIS also suggested an association between fusogenic rearrangements and a potential packing of E2 domains (clusters 8,10; **Fig 3C and 3D**). Thus, BIS proposed that interdependent rearrangements of E1 and BL could represent a hallmark of E1E2 fusogenic conformational changes.

Analysis of clusters from another HCV genotype (gt2) provided similar findings as fusion clusters also involved the BL (cluster 10,13) as well as distant blocks on E1 (cluster 6,10) and E2 (cluster 10,16) (**Fig 3E; S6–S8 Figs; S7–S9 Tables**). Statistically significant coevolution networks between E1 and BL were also found among genotype 3 sequences (**S9 Fig**). Similar networks were also found among sequences of genotype 4 to 6, but displayed poor statistical significance for the reason described above. Genotype 3 to 6a cluster positions are available through the webpages indicated in the data availability statement.

HCV E1 and E2 transmembrane were previously shown to be critical for E1E2 heterodimerization and correct E1E2 functions [26–28]. Consistently, BIS was also able to identify several coevolution clusters between the transmembrane of E1 and E2 using sequences of genotype 1 and 2 (**S10 Table**), hence strengthening the functional significance of BIS analysis.

In parallel, BIS also revealed several gt1a and gt2 structural and multifunctional clusters as supportive of the E2core central scaffold structure (**S5 and S8 Figs**), reinforcing BIS as a relevant method to model viral protein conformations. The detailed analysis of all the clusters of gt1a and 2, regardless of their attributed function, can be found in **S1 and S2 Texts** respectively.

**Fig 4:**
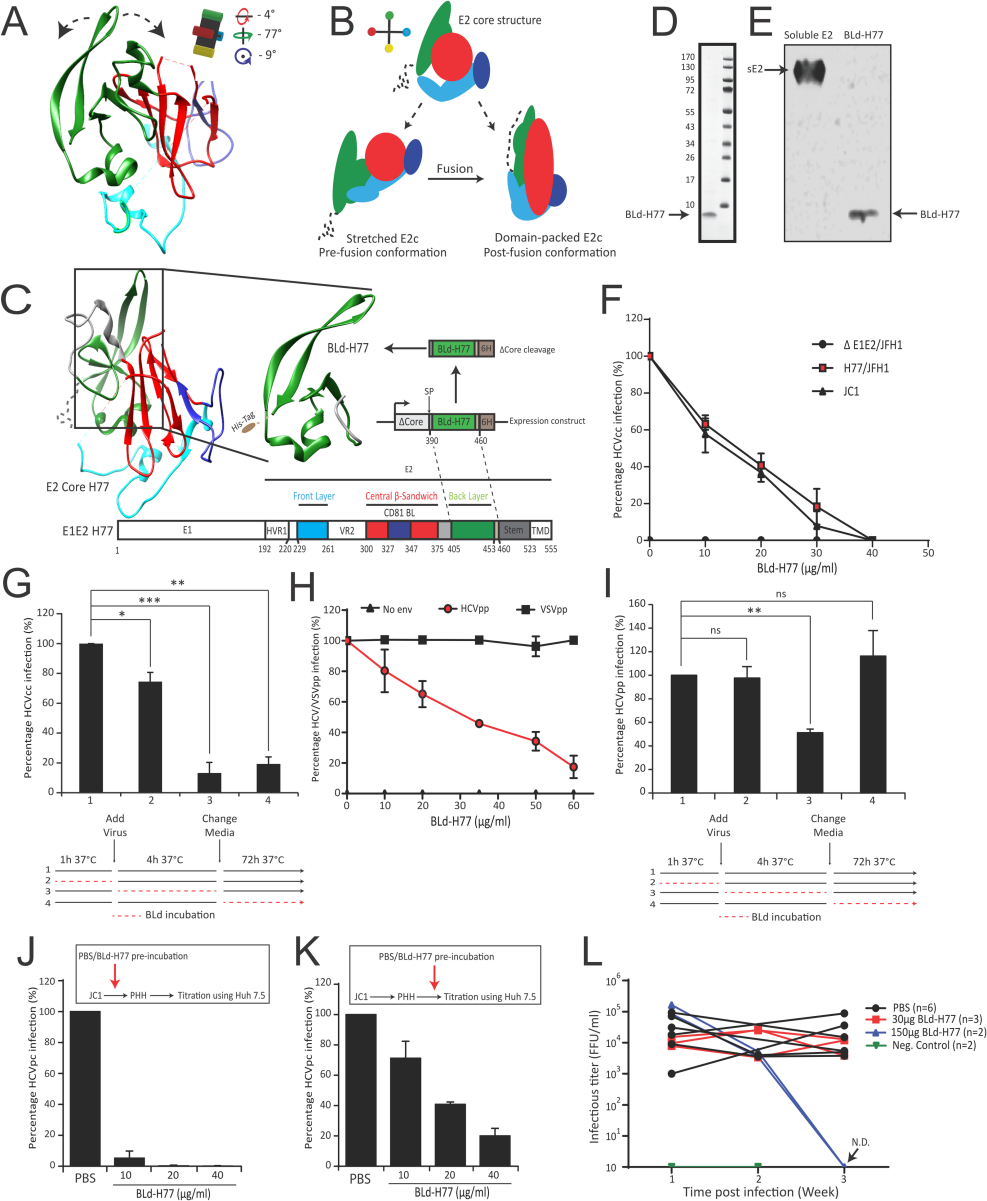
An E2 BLd-derived peptide inhibits HCV infection. (**A**) Putative BL movements that can modulate E2 rearrangements within E1E2 heterodimers. Rotation angles of the E2core structure are indicated (Reference: **Fig 3B**) and illustrated by a black cross harboring 4 color extremities. (**B**) Schematic representation of the E2 core structure and of the two hypothetical E2 conformational states prior (stretched) and following (packed) virus fusion when E1E2 are associated as heterodimer (color code and angle of E2 structures refer to **Fig 3B**). (**C**) Schematic representation of the expression construct encoding for the BLd-H77 peptide derived from the gt1a H77 E2 glycoprotein. SP, signal peptide; 6H, 6His-tag. (**D**) Detection of BLd-H77 in non-reducing condition following SDS-Page electrophoresis and coomassie blue staining. Molecular weight of the reference ladder are indicated on the left (kDa). (**E**) Detection of BLd-H77 peptide by Western Blot using a 6His-tag antibody. A 6His-tagged soluble E2 was used as a positive control. (**F**) Dose-dependent inhibition of HCVcc infection. HCVcc H77/JFH-1 or JC1 were pre-incubated with different doses of BLd-H77 (μg/ml) and used to infect Huh7.5 cells. Percentages of primary infection were calculated according to the viral titers of HCVcc particles incubated without BLd-H77 (mean ± SD; n = 3). (**G**) Kinetic of action of BLd-H77 on HCVcc infection. Huh7.5 cells were incubated 1h with BLd-H77 prior infection (35μg/ml) of HCVcc H77/JFH-1 (2), during the 4h infection (3) or following infection (secondary infection; 4). As control, cells were incubated at each step with equivalent volume of PBS (1). Percentages of primary infection were calculated according to viral titers of control conditions (mean ± SD; n = 3). Statistical significances (*p<0.05, **p<0.01, ***p<0.001) were determined for each experimental condition versus control condition (100%). (**H**) Dose-dependent inhibition of HCVpp harboring H77 envelope (HCVpp-H77) or control pseudoparticles harboring VSV-G (VSVGpp) envelope glycoprotein. Pseudoparticles were pre-incubated with different doses of BLd-H77 (μg/ml) or with PBS and used to infect Huh7.5 cells. Percentage of GFP positive cells was determined and expressed as percentages of infection, according to the viral titers of HCVpp incubated with PBS (mean ± SD; n = 3). (**I**) Kinetic of action of BLd-H77 on HCVpp infection. Huh7.5 cells were incubated 1h with BLd-H77 prior HCVpp H77 infection (50μg/ml) (2), during the 4h infection (3) or following infection (4). As control, cells were incubated at each step with equivalent volume of PBS (1). Percentages of infection according to viral titers of control conditions are reported (mean ± SD; n = 3). Statistical significances (**p<0.01, ns non-significant) were determined for each experimental condition versus control condition (100%). (**J**) Dose-dependent inhibition of HCVcc JC1 virus infection of primary human hepatocytes (PHH). JC1 particles were pre-incubated with different doses of BLd-H77 (μg/ml) or with PBS and then used to infect PHH. PHH cell culture media were harvested four days post-infection and used to infect naïve Huh7.5 cells. Percentages of secondary infection are shown and calculated according to the viral titers of JC1 virus pre-incubated with PBS (mean ± SD; n = 3). (**K**) Dose-dependent inhibition of JC1-derived HCVpc (HCV primary cell culture-derived) of Huh7.5. HCVpc particles were pre-incubated with different doses of BLd-H77 (μg/ml) or with PBS and used to infect naïve Huh7.5 cells. Percentages of primary infection using HCVpc particles are shown and calculated according to the viral titers of JC1 virus pre-incubated with PBS (mean ± SD; n = 3). (**L**). Inhibition of HCV infection *in vivo* in humanized liver mice. JC1 infectious titers (FFU/ml) were obtained from two independent mice cohorts treated with 30μg (red, n = 3) or 150μg (blue, n = 2) of BLd-H77, or with PBS (black; cohort = 6) under a “prophylactic” protocol. BLd-H77 or PBS was injected one day prior virus infection with JC1 HCVcc particles and subsequent injections were performed at day 1, 7 and 14 post-infection. Sera were harvested 7 (Week 1), 14 (Week 2) and 21 days (Week 3) post-infection. HCV viral titers were determined through infection of Huh7.5 cells with mouse sera and quantification of FFU/ml. One non-engrafted liver mice and one non-infected mice were used as negative controls (green, n = 2). N.D., non-detected.

Altogether, BIS coevolution analysis of E1E2 sequences suggested that E2 may adopt a pre-fusion structure distinct from E2core as well as yet unreported molecular rearrangements that could occur during fusion. We hence hypothesized that a movement of the BL (green; **Fig 4A**), through dialogs with E1, could mediate the evolution from a potential stretched E2 pre-fusion structure toward a domain-packed E2 post-fusion structure (**Fig 4B**).

### A soluble form of the back layer inhibits HCV infection

To challenge the potential role of the BL in E1E2 rearrangements, we generated a soluble 9kDa 6His-tagged BL domain (71 aa; named BLd-H77; **Fig 4C; S10A Fig**) from the H77 gt1a strain, detectable through Coomassie blue staining and Western immunoblotting (**Fig 4D and 4E**). Non-reducing SDS-Page electrophoresis and Dynamic Light Scattering (DLS) analysis (**S10B and S10C Fig)** confirmed the homogeneity of the peptide in solution and suggested that BLd-H77 fold as a monomer. The far UV circular dichroism (CD) spectrum of BLd-H77 eluted from size exclusion chromatography displays the molar ellipticity per residue expected for a protein folded mainly in α-helix (**S10D Fig).** We next assessed its effect on HCV infection. Interestingly, BLd-H77 was able to inhibit, in a dose-dependent manner, infection of Huh7.5 cells by replicative hepatoma cell line-derived HCV particles (HCVcc) harboring envelope glycoproteins of gt1a (H77/JFH-1) but also gt2a (JC1) (**Fig 4F; S11A Fig**). Although BLd-H77 was able to slightly inhibit HCVcc infection when pre-incubated with cells prior infection, it showed a strong potency to inhibit infection when present during the first four hours of infection (**Fig 4G**) thus suggesting that BLd-H77 might likely act on early steps of the virus life cycle. Consistently, BLd-H77 was able to efficiently inhibit infection of Huh7.5 by non-replicative retroviral pseudoparticles harboring HCV E1E2 (HCVpp) from different genotypes (**Fig 4H and 4I; S11B Fig**). This inhibition was specific as BLd-H77 was not able to inhibit infection by pseudoparticles harboring VSVG envelope (VSVGpp). Time-course experiments using BLd-H77 (**Fig 4I)** as well as two additional entry inhibitors, Bafilomycin A1 that acts on cell endosome acidification [29]) and an anti-E2 neutralizing antibody (that binds to E1E2 complexes [30]), demonstrated that BLd-H77 is an entry inhibitor that likely acts on viral particles (**S11C Fig**) but not on cells. No effect of BLd-H77 on HCV cell entry receptors expression could be observed (**S11D Fig**). Moreover, BLd-H77 was also able to inhibit cell-to-cell transmission (**S11E Fig**) in addition to cell-free infection (**Fig 4F and 4G**). Altogether, these results highlighted that BLd-H77 likely inhibits a conserved mechanism during HCV entry without affecting cell susceptibility for infection. Importantly, BLd-H77 was able to inhibit HCVcc and primary human hepatocytes-derived HCVcc virus (or HCVpc) infection of primary human hepatocytes (PHH) and Huh7.5 respectively (**Fig 4J and 4K; S11F Fig**). Finally, we assessed the ability of BLd-H77 to inhibit infection *in vivo*. BLd-H77 showed a potency to inhibit HCVcc JC1 infection over time in humanized liver mice treated with 150 μg of BLd-H77 under a prophylactic protocol (**Fig 4L**). Despite their uneven infectivity [31], our results also suggested that BLd-H77 is able to inhibit patient-derived HCV particles infection in humanized mice p = 0.02 for all quantifiable values equal and above the detection limit) as well as in PHH (**S11G and S11H Fig**). BLd-H77 had no impact on human hepatocyte viability in mice as assessed by serum albumin concentration over the course of infection (**S11I Fig**). Altogether, our results indicate that BLd-H77 is able to inhibit entry of different types of HCV particles *in vitro* and *in vivo* and thus target a strongly conserved virus entry mechanism.

### BLd-H77 binds viral particles via E2

**Fig 5:**
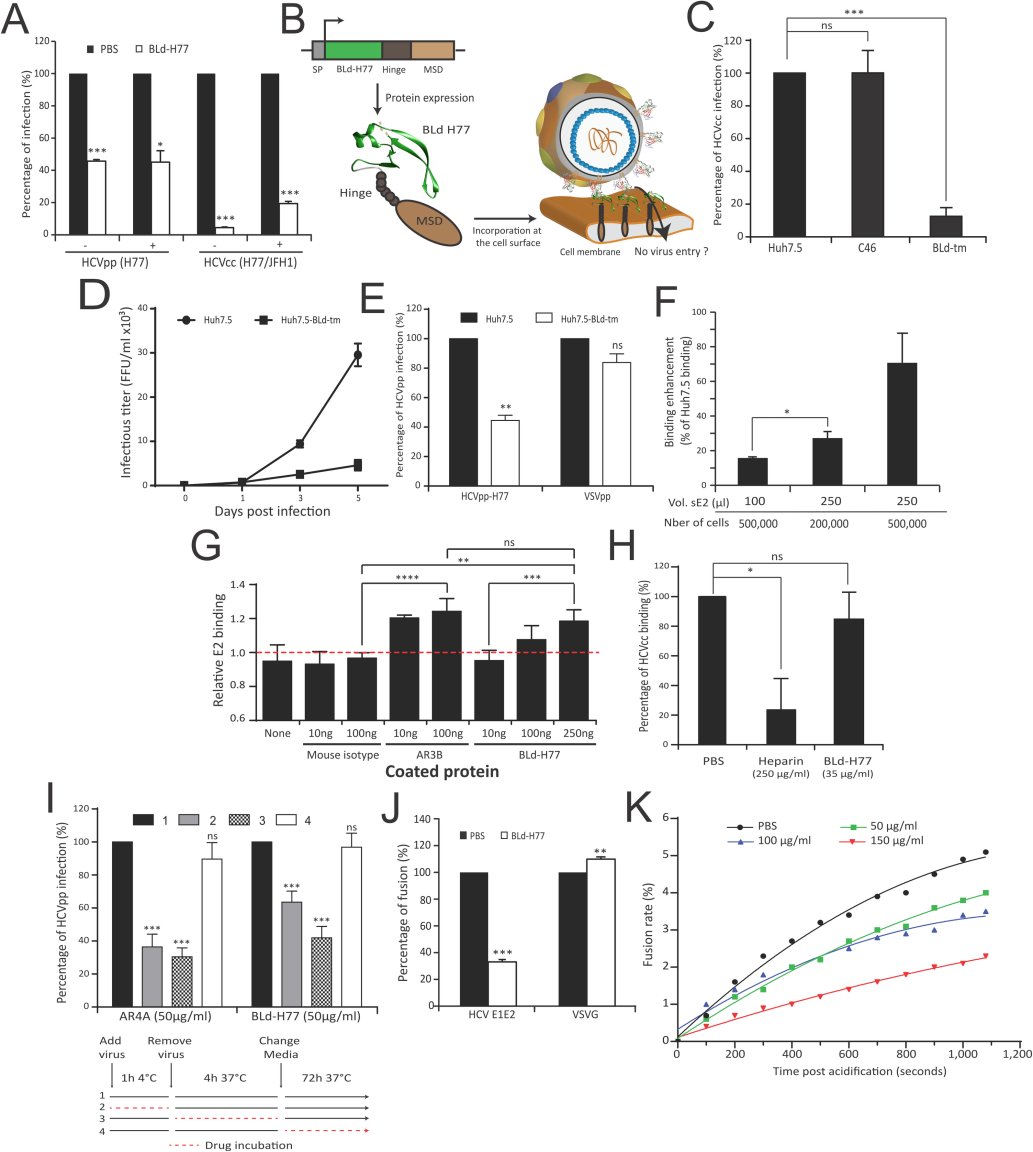
A transmembranous form of BLd-H77 inhibits HCV infection. (**A**) BLd acts onto viral particles. H77 HCVpp or H77/JFH-1 HCVcc particles were pre-incubated with BLd-H77 (50μg/ml or 35μg/ml, respectively) or with PBS. Prior to infection of Huh7.5 cells, HCV particles and BLd-H77 mixes were diluted (1/5; +) or not (-) with cell culture media resulting in a BLd-H77 concentrations inhibiting infectivity by less than 20% (see **Fig 4F** and **Fig 4H**). Viral titers of primary infection were determined 72h (HCVpp) or 4 days (HCVcc) post-infection and expressed as percentages of infection according to PBS control experiments (mean ± SD; n = 3). Statistical significances (*p<0.05, ***p<0.001) were determined for each experimental condition versus control condition (100%). (**B**) Schematic representation of the BLd-H77 transmembrane form (BLd-tm) and its possible mode of action. An IgG2 hinge region (Hinge) and the transmembrane region of the CD34 molecule (MSD) were added to the C-terminal of BLd-H77. SP, signal peptide. (**C**) A BLd-H77 transmembranous form inhibits HCVcc infection. H77/JFH-1 HCVcc were used to infect non-transduced Huh7.5, Huh7.5-C46 (C46) and Huh7.5-BLd-tm (BLd-tm). Four days post-infection, FFU titers were determined for each cell type and percentages of primary infection were normalized according to the viral titers determined following Huh7.5 infection (mean ± SD; n = 3). Statistical significances (***p<0.001, ns non-significant) were determined for each experimental condition versus control condition (100%). (**D**). Propagation of HCVcc viral particles in Huh7.5-BLd-tm cell cultures. Huh7.5 and Huh7.5 BLd-tm cells were infected with HCVcc H77/JFH-1 at a m.o.i. of 0.1. At day, 1, 3 and 5 post-infection, cell culture supernatants were harvested and used to infect naïve Huh7.5 cells. Viral titers of secondary infection were determined by NS5A immunostaining four days post-infection (mean ± SD; n = 3). (**E**) BLd-tm inhibits cell entry. Non-transduced Huh7.5 and Huh7.5-BLd-tm were infected with HCVpp H77 and VSVpp. 72h post infection, amount of GFP positive cells were quantified. Percentage of infection of Huh7.5-BLd-tm is normalized for each type of particle on the percentage of infection of non-transduced Huh7.5 (mean ± SD; n = 3). Statistical significances (**p<0.01, ns non-significant) were determined for each experimental condition versus control condition (100%). (**F**) Soluble E2 binding to Huh7.5-BLd-tm cells. Different doses of soluble E2 were mixed with different concentrations of Huh7.5 and Huh7.5-BLd-tm cells and E2 binding was quantified by flow cytometry. Results represent E2 ability to bind Huh7.5-BLd-tm cells for each condition relatively to the basal E2 ability to bind Huh7.5 cells (determined as 0% binding) for the same condition (mean ± SD; n = 3). *p<0.05. (**G**) Interaction between BLd-H77 and sE2 detected by ELISA. Different amounts of mouse IgG isotype, AR3B and BLd-H77 were coated overnight into 96-well plates. Coated peptides and antibodies were then incubated with 10ng of soluble E2 (sE2) or not. After washing, soluble E2 was detected using the rat anti-E2 antibody 3/11 and an anti-rat HRP antibody. After measurement of the optical density (O.D.) at 450nm, relative E2 binding was determined by calculating the ratio of O.D. between condition with 10ng of sE2 and no sE2, for each coating condition (mean ± SD; n = 3). **p<0.01, ***p<0.001, ****p<0.0001, ns non-significant. (**H**) BLd-H77 does not affect HCVcc binding. JC1 HCVcc particles were pre-incubated with BLd-H77 (35 μg/ml), Heparine (250 μg/ml) or PBS and mixed with Huh7.5 cells for 2h at 4. After washing, amounts of cell-associated viral particles were determined by RT-qPCR (mean ± SD; n = 3). Data are shown as percentage of binding, according to binding of HCVcc particles in control condition (PBS). Statistical significances (*p<0.05, ns non-significant) were determined for each experimental condition versus control condition (100%). (**I**) BLd-H77 can inhibit HCV entry following particle binding. HCVpp particles (HCVpp-H77) were incubated with Huh7.5 in presence of BLd-H77 (50μg/ml) or AR4A (25 μg/ml) during binding (1h at 4°C; 2), entry (4h at 37°C following binding; 3), or following entry (72h at 37°C following media change; 4). GFP levels were quantified 72h post infection. Huh7.5 infected in a similar manner but non-treated with BLd-H77 or AR4A were used as control (1). Percentages of infection were calculated based on viral titer obtained from control conditions. (mean ± SD; n = 3). Statistical significances (***p<0.001, ns non-significant) were determined for each experimental condition versus control condition (100%). (**J**) Effect of BLd-H77 on cell-cell fusion. LTR-luciferase-transduced 293T cells expressing HCV-H77 E1E2 or VSVG glycoproteins were co-cultivated with Tat-expressing Huh7.5 cells. Following pre-incubation with PBS or with 50μg/ml of BLd-H77, co-cultured cells were exposed to an acid shock (pH5) or not (pH7) and luciferase activities were determined 72h post-exposure. Percentage of fusion of HCV and VSVG glycoproteins at pH5 between control (PBS) or BLd-H77 are indicated (mean ± SD; n = 3). Statistical significances (**p<0.01, ***p<0.001) were determined for each experimental condition versus control condition (100%). (**K**) Effect of BLd-H77 on virus-liposome fusion assays. H77 HCVpp particles were pre-incubated with different dose of BLd-H77 (50μg/ml, green; 100 μg/ml, blue; 150 μg/ml, red) or not (PBS, black), and mixed with R18-labelled liposomes. Dequenching of R18 was quantified following sample acidification (pH5). Data are represented as non-linear polynomial fitted curves for each experimental condition and display the evolution of the fusion rate (%) over time. Curves are representative of three independent experiments.

We then sought to elucidate how BLd-H77 blocks HCV entry. By pre-incubating viral particles with BLd-H77 and diluting the mix prior to infection to reach a BLd-H77 concentration below its efficient neutralizing activity (determined in **Fig 4F and 4H**), we showed that BLd-H77 could irreversibly neutralize HCV particles regardless of mix dilution, hence suggesting that BLd-H77 can bind native viral particles prior viral entry (**Fig 5A**). To assess the presence of an interaction between HCV particles and the BLd-H77, we constructed a transmembrane form of BLd-H77 (called BLd-tm) (**Fig 5B**). Following lentiviral transduction, BLd-tm expression was detectable at Huh7.5-BLd-tm surface (**S12A Fig**), and did not impact HCV receptor expression (**S12B Fig**). BLd-tm expression at Huh7.5 surface, but not expression of a similar construct encoding for an anchored HIV-1 fusion inhibitor (namely C46), inhibited HCVcc propagation both in a cell-free and cell-to-cell transmission manner (**Fig 5C and 5D; S12C Fig**). HCVpp entry, but not VSVpp entry, (**Fig 5E**) was inhibited following infection of Huh7.5-BLd-tm, hence highlighting that BLd-H77 specifically inhibits HCV entry likely through binding of E1E2 glycoproteins. Consistently, more HCVpp were detected at Huh7.5-BLd-tm cell surface 4h post infection in comparison to Huh7.5 (**S12D Fig**), suggesting a potential containment of HCV particles by BLd-tm at the cell surface. The ability of recombinant soluble E2 (sE2) to bind more efficiently Huh7.5-BLd-tm cells than Huh7.5 cells in a dose dependent manner (**Fig 5F**) further suggested that virus entry is inhibited through an interaction between E2 and BLd-tm. In order to explore more precisely a putative interaction between E2 and BLd-H77, we designed an ELISA assay to quantify the ability of sE2 to be captured by coated BLd-H77. Our result showed a significant ability of sE2 to bind coated-BLd-H77 and coated-anti-E2 antibody AR3B [32] but not a coated-mouse IgG isotype (**Fig 5G; S12E Fig**). Altogether, these results strong suggest that BLd-H77 inhibit HCV entry by binding to E2 glycoprotein.

### BLd-H77 inhibits virus fusion

Next, we explored which step of HCV entry is inhibited by BLd-H77. BLd-H77 had no significant effect on attachment of HCVcc (HCVcc JC1, **Fig 5H**), HCVpp-H77 particles or soluble E2 (**S13A Fig**) on Huh7.5 cells. Using a highly specific and previously established HCVpp binding assay [21], we confirmed that BLd-H77 does not abrogate HCVpp binding to either human CD81 or SR-BI when used at a highly neutralizing concentration (**S13B–S13D Fig**). Moreover, BLd-H77 neutralizing activity was not competing with the activity of a neutralizing anti-E2 antibody known to inhibit viral particle binding [30], and their use in combination showed a synergistic neutralization effect (**S13E Fig**). Consistently, BLd-H77 could bind viral particles following their binding at the cell surface, and was shown to have its more potent neutralizing activity during post-binding steps (**Fig 5I**). Using a cell-cell fusion assay, we showed that BLd-H77 could strongly inhibit cell-to-cell fusion in comparison to control envelope glycoproteins (**Fig 5J; S14A Fig**). Importantly, cell-to-cell fusion was only inhibited when cells were incubated with BLd-H77 before low pH exposure that activate membrane fusion (**S14B Fig**), underlining BLd-H77 ability to specifically binds E1E2 pre-fusion conformations. Finally, using a HCVpp fusion assay with liposomes, which are devoid of any receptors or cell factors, the BLd-H77 inhibited fusion in a dose-dependent manner (**Fig 5K, S14C Fig**). Altogether, these results suggest that BLd-H77 specifically blocks E1E2 fusogenic rearrangements and the formation of post-fusion structures through binding to E2 protein, in accordance with BIS predictions.

### E1E2-BL dialog is critical for fusion

**Fig 6:**
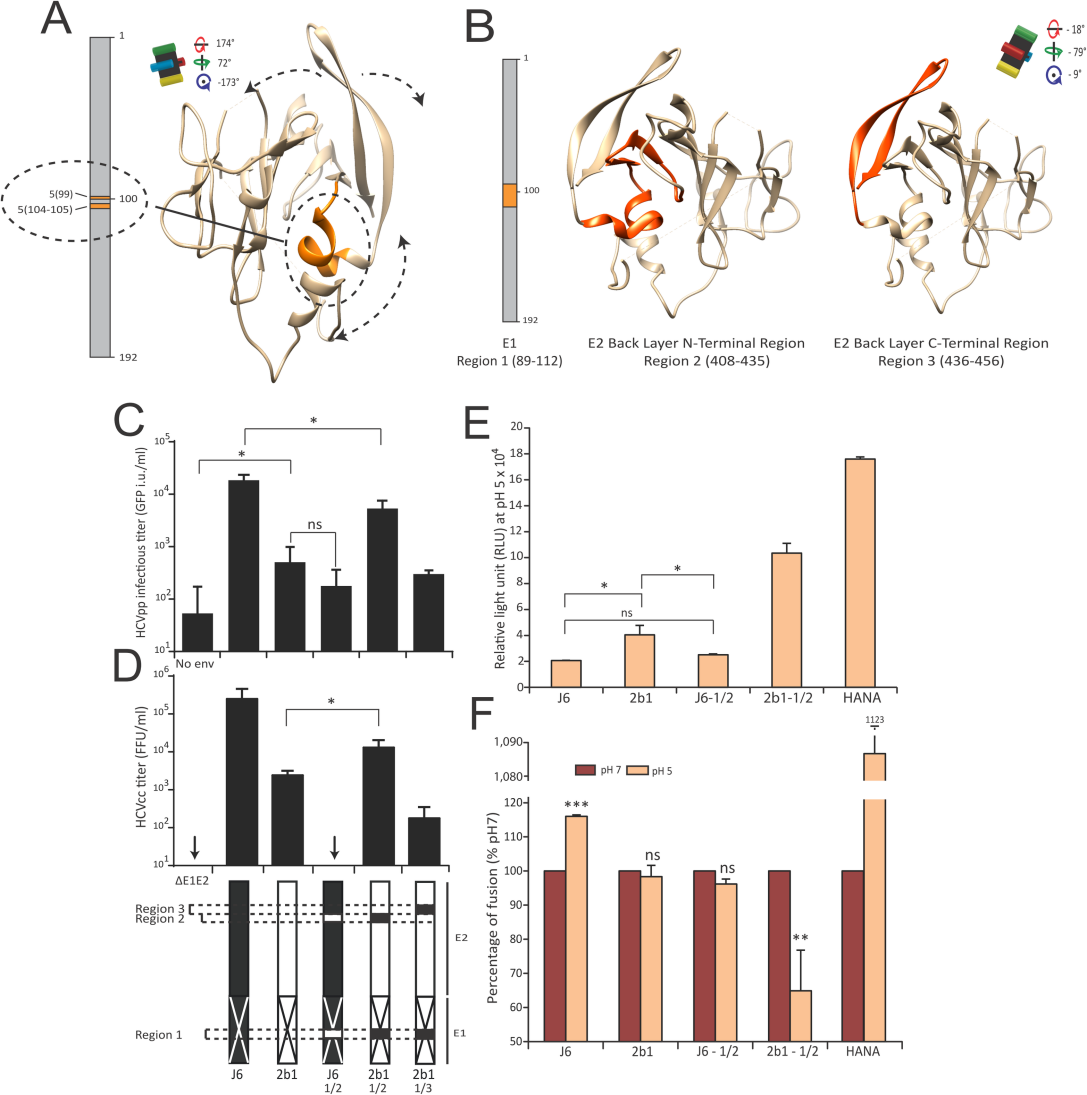
A dialog between E1 residues and the BL modulates virus entry and fusion. (**A**) Representation of the gt2 fusion cluster 5 (orange; dotted circle and linked by a bold line) as a putative mediator of E2 BL rearrangements and fusogenic conformational changes. Putative movements of the E2 BL are indicated by dotted double arrows. Rotation angles of the E2core structure and black cross are indicated as in **Fig 3B**. (**B**) Regions of interest to study the role of the cluster 5 (orange) and design of the gt2 chimera. Three regions were defined as cluster 5 blocks. One is located on E1 (30 aa; Region 1) and two are located in E2 BLd (Region 2: E2 408–436, N terminal region; Region 3: E2 437–456, C terminal region). (**C-D**) A dialog between E1 and the BL domain regulates virus entry. Infectious titers of HCVpp (GFP i.u./ml, **C**) and HCVcc (FFU/ml, **D**) viral particles harboring J6 (black), 2b1 (white) and J6/2b1 E1E2 chimera. Swapped regions (1, 2 or 3) are represented by white (2b1) or black (J6) boxes inserted into J6 or 2b1 linear representations, respectively (bottom). Infectious titers were quantified 72h (HCVpp) or 4 days (HCVcc) post infection of Huh7.5. (mean ± SD; n = 4); *p<0.05.; ns non-significant. (**E-F**) A dialog between E1 and the BL domain regulates membrane fusion. Cell-cell fusion induced by the different E1E2 chimera (as described in **Fig 5J**). HANA (Influenza Hemagglutinin-Neuraminidase) envelope glycoproteins were used as positive control. Data are presented as relative light unit (RLU) at pH5 (**E**) or as percentage of fusion (**F**) where pH7 RLU is considered as 100% fusion rate for each chimera (mean ± SD; n = 3); *p<0.05, **p<0.01, ***p<0.001 ns non-significant. For panel (**F**), statistical significances were determined for each experimental condition versus control condition (100%).

Beside highlighting potential E1E2 rearrangements during fusion, BIS can identify pairs of residues that need to mutate in concert to guarantee structural compensations and proper viral fitness. Indeed, we have previously demonstrated how HCV entry depends on strain-specific dialogs between particular E1 and E2 domains [21]. Thus, we aimed at addressing whether BIS is able to unveil specific dialogs between residues of E1 and the E2 BL that modulate HCV fusion. The BIS predictions identified the multifunctional gt2 cluster 5 (**S7–S9 Tables**) as an interesting candidate for supporting E1 and E2 dialogs, BL movements and transition from E1E2 pre-fusion to post-fusion states. This cluster, similar to gt1a fusion cluster 5, linked two central blocks in E1 (residues 104, 105, and 109) and one block in E2 BL (residues 427 to 436; orange; **Fig 6A**). In order to challenge its potential role during fusion, we used cluster 5 blocks to guide the rational design of E1E2 chimeric constructs. The E1 region containing two cluster 5 blocks (**Fig 6B**; Region 1) and E2 BL regions containing the other cluster 5 block (**Fig 6B**; Region 2) or not (**Fig 6B**; Region 3, non-coevolving cluster 5 block as a control of specificity) were swapped between two E1E2 heterodimers from different gt2 strains, one allowing efficient HCVpp entry (J6) and another one mediating sub-optimal HCVpp entry (2b1) (**S15A and S15B Fig**). All chimeras were similarly expressed and incorporated (**S15C Fig**). Although J6 chimera carrying 2b1 cluster 5 regions (J6-1/2) displayed a poor entry efficiency, similar to 2b1 parental glycoproteins, a 2b1 chimera carrying J6 cluster 5 regions (2b1-1/2) exhibited >10-fold improved entry ability (**Fig 6C**). In contrast, 2b1 chimera carrying J6 Region 1 and 3 (2b1-1/3; **Fig 6C**) were not optimal for entry, hence suggesting that the E1 cluster 5 blocks and the N-terminal half of the BL domain are involved in a dialog regulating virus entry. Production and titration of HCVcc particles harboring these different chimeric envelopes confirmed these results (**Fig 6D, S15D Fig**). Interestingly, 2b1-1/2 also displayed improved ability to mediate cell-to-cell fusion (**Fig 6E**) as well as higher fusion efficiency at neutral pH than at acidic pH (**Fig 6F**), suggesting that this chimera exhibited an E1E2 conformation already primed for fusion at neutral pH. In contrast, J6-1/2 chimera did not increase J6 fusion efficiency and abrogated E1E2 sensitivity to low pH at levels similar to those of 2b1 (**Fig 6E and 6F**). Altogether, consistently with BIS predictions, these results suggest that conserved interplays between the central region of E1 and the N-amino-terminus region of the BL likely govern E1E2 fusogenic conformational states. Our results also support the maintenance of such dialogs through coevolution as they appeared to be mediated by genotype-specific regions of E1 and the BL.

### BIS identify genotype 1 coevolving amino acids in E1 and the Stem region that regulate HCV fusion

**Fig 7:**
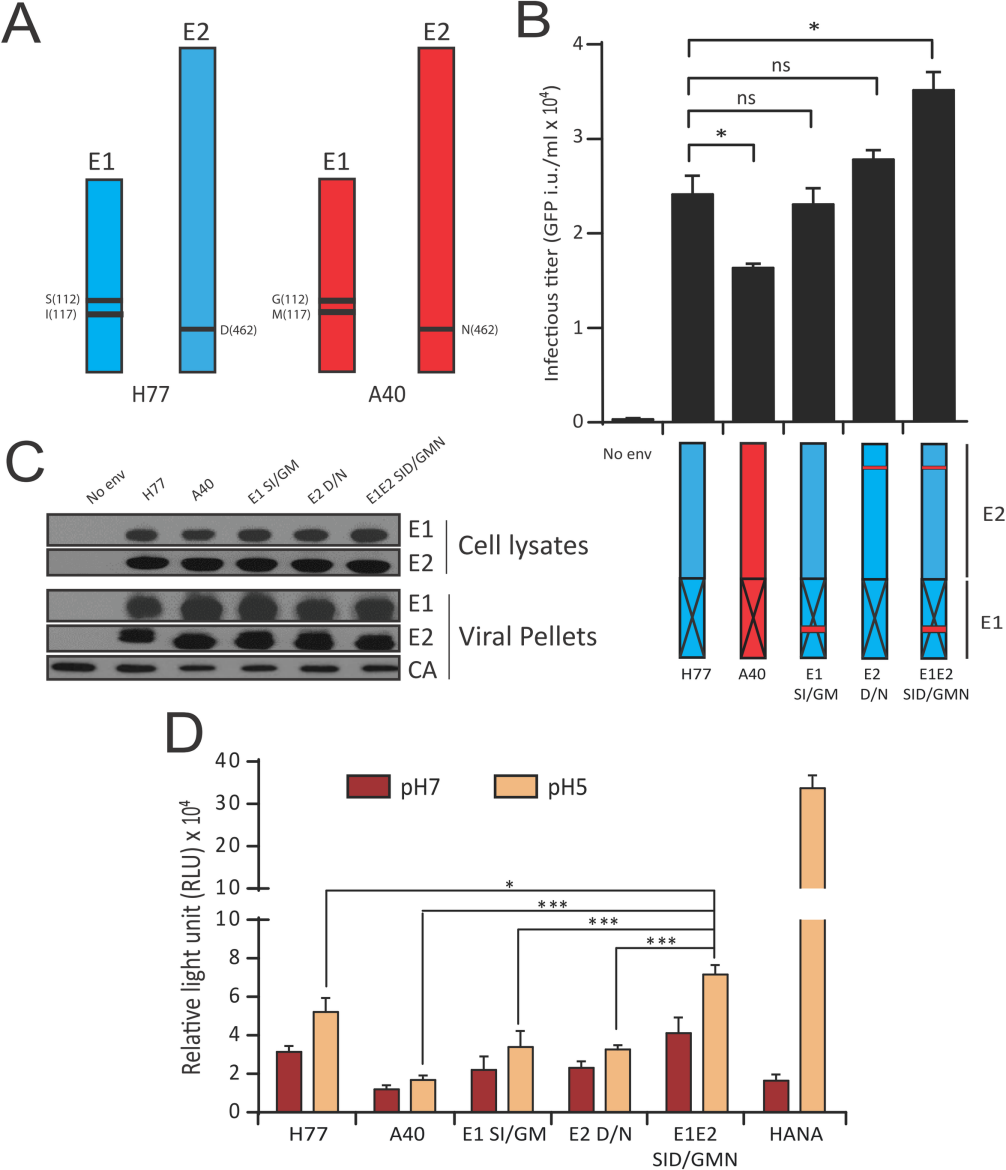
BIS uncovers a coevolving signal between E1 and the Stem region that regulate HCV fusion. (**A**) Position of the three amino acid residues that differs between H77 (blue) and A40 (red) and are hypothesized to coevolve according to BIS prediction (gt1a cluster 5). The three H77 amino acids will be replaced by A40 residues individually or altogether to challenge BIS prediction. (**B**) Impact of the E1/Stem coevolution signal on HCV entry. Infectious titers of HCVpp viral particles harboring H77 (blue), A40 (red) and H77/A40 E1E2 chimera were determined. Two E1 H77 residues (S112, I117), a single H77 E2 residue (D462) or both (S112, I117, D462) were introduced into E1E2 A40. The different envelopes were incorporated at the surface of HCVpp, then used to infect Huh7.5. Infectious titers were quantified 72h post infection by flow cytometry (mean ± SD; n = 3). *p<0.05, ns non-significant. (**C**) H77/A40 E1 and E2 chimera expression and incorporation onto HCVpp. Expression in transfected 293T cells (Cell lysates) and incorporation onto concentrated pseudoparticles (Viral Pellets) of E1 and E2 from the different H77/A40 chimera. Detection of E1 and E2 onto pseudoparticles harboring no envelope glycoproteins was used as negative control. MLV-Capsid (CA) was detected to control equivalent HCVpp production between chimera. (**D**). Impact of the E1/Stem coevolution signal on HCV fusion. LTRhiv-luciferase vector transduced 293T cells expressing the different E1E2 H77/A40 chimeric envelope glycoproteins were co-cultured with Tat-expressing Huh7.5 cells. Co-cultured cells were exposed to an acid shock (pH5, orange) or not (pH7, red) and luciferase activities were determined 72h post-exposure. Results are presented in relative light units (RLU) for each experimental condition (mean ± SD; n = 3). *p<0.05, ***p<0.001.

To extend our transfer between the bioinformatics identification of coevolving amino acid clusters to the functional linkage of these domains, we tested the ability of BIS to pinpoint specific amino acids located in other regions than BL and furthermore, in the context of another genotype than genotype 2 (studied above). For this purpose, we sought to challenge a gt1a fusion cluster identified by BIS (Fusion cluster 5, **S4–S6 Tables**), which involved four residues within E1 central region (position 78 and 113–115) and a domain of 10 amino acids (452–461) within the Stem region. We employed two poorly divergent genotype 1a E1E2 sequences, H77 and A40, that displayed two E1 (SI/GM; position 112 and 117) and one E2 (D/N; position 462) amino acid differences located at the borders of the gt1a fusion cluster 5 blocks (**Fig 7A; S16A Fig**). Unlike J6 and 2b1, the level of functionality of H77 and A40 were relatively close (2.4x10^4^ and 1.6x10^4^ GFP i.u. per ml respectively) despite being significantly different (**Fig 7B**), hence making it challenging to predict the influence of residue swaps on envelope functionality. H77 chimera harboring both swapped E1 and E2 A40 residues significantly impacted HCVpp infectivity, although swapped E1 or E2 residues alone did not impact H77 functionality (**Fig 7B**) despite similar E1E2 expression and incorporation (**Fig 7C**). Importantly, H77 chimeras harboring only the E1 or E2 A40 residues showed defect for cell-cell fusion compared to H77, although fusion ability of the H77 chimera harboring both the E1 and E2 mutations were enhanced (**Fig 7D**). Altogether, consistently with BIS predictions, our results suggest that these E1 and E2 residues −and to larger extend the E1 central region and the E2 stem− are part of a coevolving network that regulates the fusogenic properties of gt1a viral envelope.

## Discussion

*Flaviviridae* are cause of many health concerns worldwide. A better understanding of the molecular processes regulating the life cycle of these viruses is critical for the design of potent anti-viral therapies. By taking advantage of the high-mutation rate of these viruses, coevolution analysis represents a valuable approach to decrypt viral protein functional rearrangements and provide basis for their inhibition. Here, we employed a recent coevolution analysis method, BIS [22–24] to provide a proof of concept of such approach.

**Fig 8:**
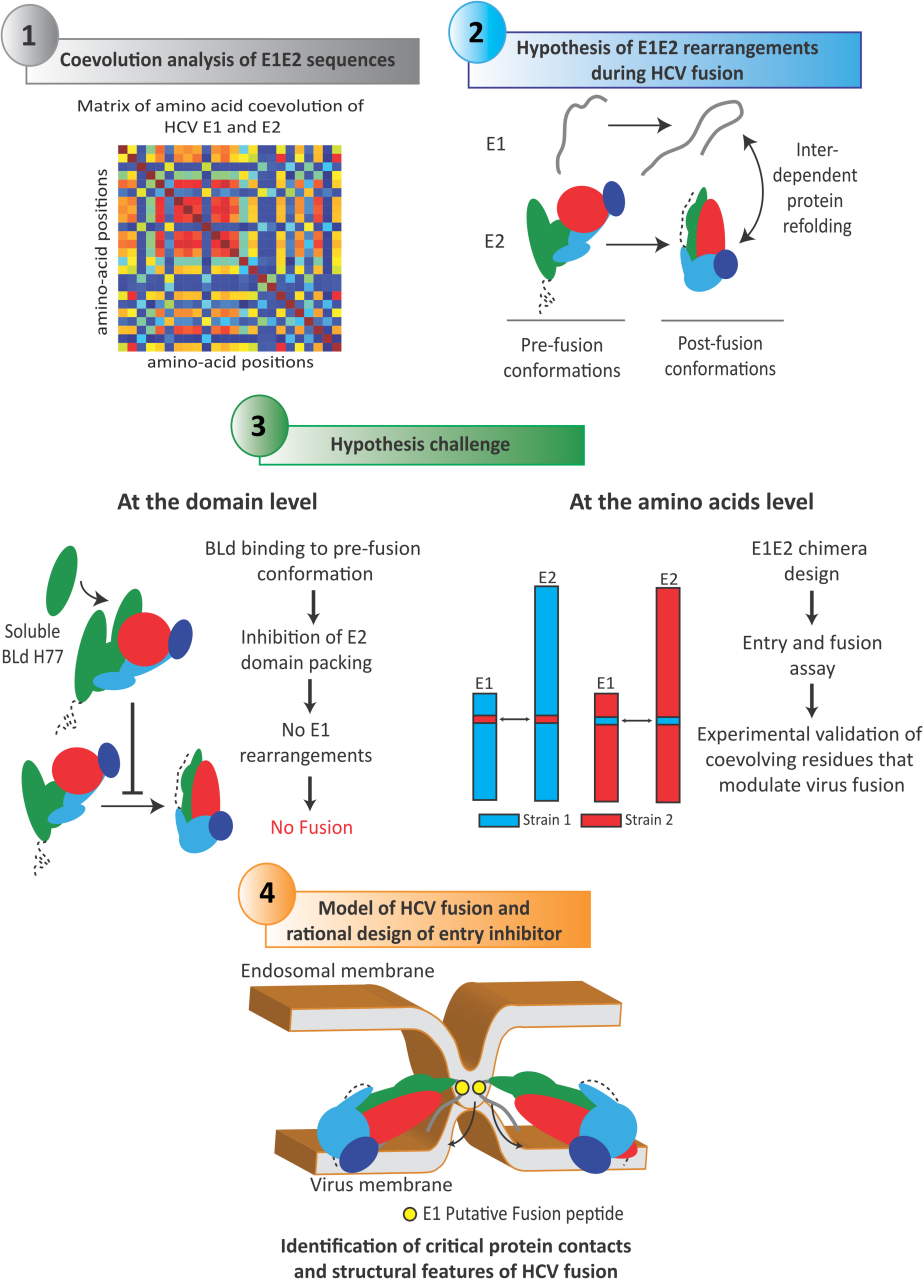
BIS as a methodology to decrypt virus entry mechanisms. Schematic representation of the experimental approach employed in this study, from BIS computational analysis to the design and challenge of a mechanistic model of viral fusion. Following sequence analysis, matrix of E1E2 amino-acid coevolution were generated by BIS for different HCV genotypes. Plotting of matrix coevolution networks onto E2core structure unveiled a potential scenario of E1 and E2 rearrangements during HCV fusion, which involved the BL domain of E2. At the protein domain level, the construction of a soluble form of the BL and the conduction of several experimental assays supported such hypothesis. In parallel, at the amino acid level, the experimental validation of coevolution signals between specific residues of E1 and of the BL highlighted the critical role of E1-BL networks in regulating fusogenic rearrangements (and more generally, the critical role of coevolving networks between E1 and E2 C-terminal regions). Altogether, this approach allows us to propose a HCV fusion model where BL movements and E1 refolding are critical in the induction of E1E2 interdependent, fusogenic rearrangements. By being applicable to other viral proteins and viruses, such approach provides opportunities to uncover undescribed viral-mediated mechanisms and design innovative translational strategies for their inhibition.

Coevolution signals detected within DENV E and Pr recapitulated several structural features of DENV E/E-Pr protein complexes in different conformational states, hence highlighting the structural accuracy of BIS predictions. Coevolution analysis of HCV E1E2 sequences from several genotypes and sub-types led to the identification of several coevolution signals in HCV E1E2 and suggested that E1 and E2 are strong coevolving partners that refold interdependently during fusion (**Fig 8**). Importantly, the E2 BL emerged as a key element of these rearrangements that could mediate the transition of E1E2 complex from a pre-fusion to a post-fusion conformation. We propose that during this transition, the endogenous E2 BL packs with the front sheet of E2. Thus, a recombinant soluble BLd-H77 could compete with the endogenous E2 BL and block HCV fusogenic rearrangements, then leading to inhibition of membrane fusion (**Fig 8**). Such competition is consistent with the idea that E2 may harbor a stretched pre-fusion structure exposing internal epitopes. The existence of other E2 structures is also supported by the ability of neutralizing antibodies to target an epitope that is not exposed in the E2core structure (i.e. aa305-324 [33]). Whether the entire BLd-H77 or specific BL amino acids regions are sufficient to inhibit HCV fusion remain to be determined. However, a recent study aiming to screen E2-derived peptide inhibiting HCV infection did not identify peptides within the BL [34], suggesting that a large fraction of the BL, instead of a specific amino acid region, is likely to be required to inhibit HCV entry. This hypothesis reinforces the idea of a physical, but not functional, competition between the endogenous BL and BLd-H77. Our work also suggests that the E2 BL acts in close collaboration with E1 and that these domains are probably in close proximity in E1E2 heterodimer. Indeed, E2core structure is highly concealed by glycans at the exception of the BL [7]. This, combined with the fact that E1 and BL do not seem to represent preferential targets of anti-HCV neutralizing antibodies, suggests that the BL region and E1 may conceal respective epitopes. The critical interplay between BL and E1 during fusion was also demonstrated at the amino acid level, as BIS was able to accurately identify coevolution signals between specific residues of E1 and the BL that tightly regulate HCV fusion (**Fig 8**).

By employing an original multi-disciplinary approach combining computational analysis and experimental assays, our work sheds light on potentially important structural and functional features of the HCV fusion mechanism. Although the clear roles of E1 and E2 during the HCV fusion process still remain to be better defined through the structural resolution of the E1E2 heterodimer at different pH, our work initiates a path toward an experimentally-supported model for HCV-cell fusion.

In this putative model, E1 would play the role of a fusion protein protruding onto the virus surface whereas E2 would be a receptor binding protein and a fusion chaperone concealing E1 epitopes. Although E2core is a truncated E2 protein and may not exhibit full E2 properties, E2core does not respond to pH variation [6]. This, combined with the fact that E2 does not harbor fusion protein structural features, could suggest that E2 needs to be associated with E1 to undergo conformational changes and to chaperone E1 fusion-promoting rearrangements. Interestingly, non-conserved E1 residues swapped between the J6 and 2b1 envelope (Fig 6) were located at a very close proximity upstream of the putative E1 fusion peptide (CSALYVGDLC) [11–16]. This result may suggest that the interaction of these E1 residues with E2 BL could participate to critical E1-E2 rearrangements leading to the fusion peptide insertion into the endosomal membrane. Following receptor priming [35] and insertion of a putative E1 fusion peptide into the endosomal membrane, HCV fusion could be triggered by a fold-over of the C-terminal domain of E1 toward its N-terminal domain. This rearrangement could be mediated by E2 BL movements, or alternatively, could promote E2 BL movements. Overall, this interdependent refolding would result in the packing of the BL and of the E2 C-terminal region toward the E2 front sheet, and to the fusion of the host and viral membranes (**Fig 8**).

The Ig-fold β-sandwich structure of E2core has been proposed to display similarities with domain III or B from class II fusion proteins [7]. By chaperoning E1 fold-over and membrane fusion, our results support that E2 function could be related to a domain III-like, as recently suggested [36]. Further structural studies aiming to resolve the full structure of the entire HCV heterodimer in pre- and post-fusion conformations will be needed to decipher the integral fusogenic rearrangements. Despite the fact that the structural differences detected for pestivirus E2 vs. HCV E2 glycoproteins makes unlikely that these viruses harbor a similar fusion mechanism, it is possible that their E1 proteins could be derived from a common ancestor and represent so far a potential new class of fusion protein as suggested by others [9,18]. Thus, although the resolution of the E2core structure has undermined the role of E2 as a fusion protein, HCV fusion is likely mediated by two interdependent partners that display original structures and conformational changes. To our knowledge, our data provide unique evidence that a component (in this case, the BL) derived from a receptor-binding protein with no fusion peptide can modulate viral fusion rearrangements, hence highlighting HCV fusion as a unique mechanism among known enveloped viruses.

A primary limitation of current coevolution analysis approaches relies on the availability of a large number of sufficiently divergent evolutionarily-related sequences [37]. Such sets of sequences constitute the bottleneck for today’s coevolution analysis methods. We have shown previously that BIS can overcome these limitations and can address coevolution of conserved sequences such as viral genotype sequences [22–24]. Consistently, we failed to detect coevolution signals within our sets of E1E2 sequences when employing other existing coevolution methods (such as DCA, PSICOV and EVcouplings [38–40]), methods that we previously discussed in [23].

In strong contrast to our previous work [23] that was critical to understand the computational power of coevolution analysis applied to viral sequences, our current study goes beyond the speculative and computational predictions and is fundamental is several ways. First, exploring coevolution signals when no or little structural and functional information are available remain highly challenging and hamper the delineation of undescribed viral protein-mediated processes. As coevolution signals imply structural or functional associations between coevolving residues [22,23], our work shows that the BIS method is able to successfully highlight E1E2 contacts that likely orchestrate structural rearrangements of the heterodimer complex. In this respect, our work constitutes an important demonstration of BIS ability to decode structural features of major conformational change in proteins families characterized by few and conserved sequences. Hence, our work provides evidence that computational analysis of coevolution with BIS can be fruitfully used to find direct (and possibly indirect) contacts between proteins where the three-dimensional structure and rearrangements of protein complexes are not known. Second, it demonstrates that coevolution analysis can highlight the existence of conformational changes in proteins through pairs of coevolving residues that are not in contact in the known crystal structure. Many of the coevolution analysis tools developed in recent years (such as DCA and DCA-like approaches) are detecting “direct contacts” and justify their success by using the 3D crystal as their evidence of true positive predictions. In our study, we show that this idea only represents a part of the truth. Proteins are more complex systems, undergoing different structural conformations during their lifetime, and that evolutionary signals code not only for direct interaction but also for intermediate folding states and alternative structural conformations. Third, we advance in the comprehension of how computational techniques can be used to help revealing protein “contacts” that are biologically interesting, within and among structures. Finally, we provide experimental evidence of the biological significance of the coevolution signals for viral genotype sequences, hence allowing for a rapid identification of critical residue contacts regulating protein functions and conformations.

Beyond uncovering important features of the HCV fusion mechanism, our study provides altogether a proof of concept that coevolution can be successfully harnessed to decrypt viral protein rearrangements and interactions, as well as to expand our knowledge of viral proteins-mediated biological processes. Virus entry mechanisms and envelope glycoproteins conserved epitopes represent valuable targets for the development of drugs and innovative vaccine strategies against challenging human pathogens [41–46]. By unscrambling key protein interactions or rearrangements, our work demonstrates that coevolution predictions can be of considerable value for stimulating a fast-tracked design and screening of innovative translational approaches and antiviral strategies unimpeded by virus plasticity.

## Materials and methods

### Ethics statement

Experiments were performed in accordance with the EU guidelines (Directive 2010/63/EU) on approval of the protocols by the local ethical committee (Authorization Agreement C2EA-15; Ethic commitee: Comité d’Evaluation Commun au Centre Léon Bérard, à l’Animalerie de transit de l’ENS, au PBES et au laboratoire P4 (CECCAPP), Lyon, France.

### Cell lines, reagents and biological materials

Human Huh-7.5 (kind gift of C. Rice, Rockefeller University, NY), BRL3A rat hepatoma (ATCC CRL-1442), and 293T kidney (ATCC CRL-1573) cells were grown in Dulbecco’s modified Eagle’s medium (DMEM) supplemented with 10% fetal calf serum (Invitrogen). Primary human hepatocytes (PHH; BD Biosciences) were centrifuged using a F-12 HAM medium (Sigma) and seeded overnight in collagen-coated 48 well plate (1x10^5^ cells/well) into Gentest seeding medium (BD Biosciences) completed with 5% FCS. The next morning, PHH were washed and cultured with the culture medium for PHH William’s E medium (W4128, Sigma) supplemented with 7,5% BSA, 1% ITS (insulin transferrin selenium, Gibco), 10-7M of Dexamethasone (Sigma), 1% of non-essential amino acids (Gibco), 1% of Glutamax (Gibco) and 1% Penicillin-Streptomycin solution (Gibco). Sera containing HCV particles were obtained from a gt1b infected patient (Hôpitaux Universitaires de Strasbourg, Strasbourg, France) and were subsequently amplified in uPA-SCID humanized liver mice. Viral loads (RNA copies number/ml) were determined by RT-qPCR using a clinical diagnostic kit (Abbot).

### Antibodies

The rat anti-E2 clone 3/11 [47], the mouse anti-E2 AP33 (Clayton et al., 2002) and the conformational mouse anti-HCV E2 H53 [48] are kind gifts from J. McKeating (University of Birmingham, UK), Arvin Patel (MRC—University of Glasgow Centre for Virus Research, Glasgow, UK) and J. Dubuisson (Institut Pasteur de Lille, FR) respectively. AR3B [32] and AR4A [30] antibody are a kind gift from Mansun Law (The Script research institute, San Diego, USA). MLV Capsid was detected by a goat anti-MLV-CA antibody anti-p30 (Viromed). HCVcc foci forming units were stained with a mouse anti-HCV NS5A antibody 9E10 [49], a kind gift of C. Rice (Rockefeller University, NY, USA). Human CD81 were detected with JS81 mAb (BD Biosciences), human SRB1 with CLA-1 mAb (BD Pharmingen), human Claudin-1 with the MAB4618 mAb (R&D Syst.) and human Occludin with an anti-Occludin mAb targeting the C-terminal region of the protein (Laboratories Inc.). A Mouse anti-Human IgG2 antibody targeting human IgG2 hinge region (Novus Biological) was used to quantify BLd-tm expression at cell surface. A rabbit 6His-tag antibody (Pierce antibody) was used to detect soluble E2 and BLd-H77 6His-tag.

### BLd-H77 construction

In order to construct a relevant BLd soluble peptide derived from a genotype 1a sequence, we took into account the BL borders previously suggested (**Fig 3B**, see also reference [7]), but also aimed to refine these borders using the locations of BIS coevolution networks of genotype 1a. Indeed, BIS suggested that a 15 amino acids extension from residue 390 to 405, classified previously as an undefined domain [7] between the central Ig scaffold and the E2 BL, contained blocks (**S4 Fig**, block 7–1) that coevolve with another region of the BL (block 7–2), forming altogether a fusion cluster (cluster 7). Hence, in order to rigorously challenge BIS predictions that suggested a potential redefinition of the BL, we constructed a soluble BLd peptide from residue 390 to 460. Hence, DNA sequence of HCV E2 coding for the residues 390 to 640 was amplified from a genotype 1a envelope H77 (AF009606) cDNA sequence. This sequence was subcloned into a phCMV plasmid to fuse the last 18 amino acids from the C-term part of HCV core that act as a signal peptide, and a CH3-terminal 6 Histidine-tag was added. The resulting peptide of 77 amino acids (71+6) was then named as BLd-H77.

### BLd-H77 production, purification and analysis

BLd-H77 was expressed in 293T following transient transfection and purified from cell culture supernatant (OptiMEM) by fast protein liquid chromatography on a Superdex G-75 gel filtration column (GE Healthcare). BLd-H77 was re-suspended in 1XPBS. The concentration of purified BLd-H77 peptides was determined by absorption at 205 nm. The mass of BLd-H77 peptide was measured by ESI mass spectrometry using a Finnigan LCQ ion trap mass spectrometer (Thermo Electron Corporation). Analysis by SDS-PAGE electrophoresis was performed using a standard Tris–glycine system and 11% acrylamide gels, in reducing or non-reducing condition. Electrophoresis was followed by either coomasie blue staining or western immunoblotting with an anti His tag antibody.

### BLd-tm construction, expression and transduction

An anchored form of BLd-H77 was engineered by adding a hinge region (human IgG2) and the transmembrane domain of the CD34 protein to the E2 BLd. The construct, named BLd-tm, was then inserted into Gae-SFFV-IRES backbone harboring selectable marker gene P140K MGMT. A similar construct, but encoding for a HIV-1 gp41 fusion inhibitor peptide [50], namely C46, was used as a control. Construct details are available upon request. Lentiviral vectors transducing BLd-tm or C46 were produced from 293T cells. Stable expression in Huh-7.5 was obtained by transduction with vector particle-containing supernatants of 293T producer cells, followed by selection of *O*^6^-benzylguanine and BCNU. BLd-tm expression in Huh-7 cells was quantified by FACS analysis using a mouse anti human IgG2 and an anti-mouse APC antibody.

### Proteins expression staining

BRL cells expressing CD81 or SRBI were washed and stained for 1h at 4°C with a mouse anti-human IgG2 antibody (for detection of C46 and BLd-tm), an anti-CD81 (JS81) and with an anti-human SR-BI (CLA-1) respectively. Cells were then washed and incubated with a secondary anti-mouse or rat APC antibody for 1h at 4°C. Cell surface expression levels were then quantified by flow cytometry (FACS CANTO II–BD Biosciences). For HCV receptor detection on Huh7.5 and Huh7.5-BLd-tm cells, cells were fixed with 2% formaldehyde for 20 min at room temperature and washed. For Occludin staining, cells were permeabilized with Perm/Wash Buffer (BD Biosciences) for 15 min at 4°C prior staining. Human CD81 was stained with JS81 mAb, human SRBI with CLA-1 mAb, human Claudin-1 with the MAB4618 and human Occludin with an anti-Occludin mAb targeting the human Occludin C-terminal region. To characterize the effect of BLd-H77 on HCV receptor expression, Huh7.5 were incubated overnight with BLd-H77 (50μg/ml) prior staining.

### Production of HCVpp, infection and neutralization assay

HCVpp were produced as previously described [21,51] from 293T cells cotransfected with a murine leukemia virus (MLV) Gag-Pol packaging construct, an MLV-based transfer vector encoding the green fluorescent protein, and E1E2 envelope expression constructs H77 (AF009606), A40 (unreferenced), UKN1B 12.6 (AY734975), UKN2A 2.4 (AY734979), JFH-1(AB047639), J6 (AF177036), UKN2B 2.8 (AY734983), UKN3A 1.9(AY734985), UKN4 21.16 (AY734987), UKN5 14.4 (AY785283), HK 6A-2.1 (FJ230883) or control envelope HA-NA (CY077420) and VSV-G (AJ318514). All chimeric J6/2b1 E1E2 heterodimers were constructed by molecular cloning, PCR and/or digestion between the genotype 2a envelope J6 (AF177036) and a genotype 2b envelope UKN-2b1 (unreferenced). All chimeric H77/A40 E1E2 heterodimers were constructed using a similar strategy between the genotype 1a envelope H77 (AF009606) and A40 (unreferenced, but previously employed [21]). 72 to 96h following infection, percentage of infected cells was quantified by FACS Canto II or LSRII (BD Biosciences) to quantify GFP expression. For BLd-H77 dose-dependent neutralization assay, HCVpp H77 or VSVGpp were pre-incubated with different concentrations of BLd-H77 or with PBS for 1h at room temperature and were then used to infect Huh7.5. For the time-course neutralization assay, Huh7.5 were infected with HCVpp H77 for 4h prior washing. PBS, BLd-H77 (50 μg/ml), Bafylomycin A1 (20nM) or AR4A (25 μg/ml) were added into cell supernatant for 1h prior infection, during infection or after infection. Percentage of infected cells was determined 72h following infection. For HCVpp co-neutralization assay, HCVpp H77 pseudoparticles were pre-incubated for 1h at room temperature with BLd-H77 alone (35 or 50 μg/ml), AR4A alone (2 or 17 μg/ml) or with both BLd-H77 and AR4A (35 and 2 μg/ml, or 35 and 17 μg/ml). Pre-mixes were then used to infect Huh7.5 and media was changed 6h post infection. GFP intracellular levels were quantified 4 days post infection by flow cytometry. For HCVpp post-binding neutralization assay, HCVpp-H77 pseudoparticles were incubated with Huh7.5 in presence of BLd-H77 (50 μg/ml) or AR4A (25μg/ml) for 1h at 4°C (binding), for 4h at 37°C following binding (entry), or for 72h following entry (post-entry). As control, Huh7.5 were incubated and infected with HCVpp-H77 in a similar manner but not treated with BLd-H77 or AR4A.GFP intracellular levels and related percentage of infection were then quantified by flow cytometry. For HCVpp containment assay on Huh7.5-BLd-tm, Huh7.5 or Huh7.5-BLd-tm were infected or not with HCVpp H77 for 5h. Then, cells were washed and E2 cell surface expression was determined by flow cytometry following staining using the anti-E2 H53 antibody and a secondary anti-mouse APC antibody. Huh7.5 and Huh7.5-BLd-tm were infected similarly and GFP expression of infected cells was analyzed 72h post-infection.

### Expression and incorporation of E1E2 glycoproteins onto HCVpp

Transfected 293T cells were lysed and nuclei were removed by centrifugation at 12 000 rpm for 10 min. HCVpps were purified and concentrated from the cell culture medium by ultracentrifugation at 82,000xg for 1h 45 min through a 20% sucrose cushion. Cell lysates and viral pellets were subjected to western blot analysis using 3/11 anti-E2 antibody and an anti-MLV-CA antibody as described previously [21].

### HCVcc production and infection

Plasmid pFK H77/JFH1/HQL (kind gift of R. Bartenschlager), termed as H77/JFH-1, displaying HCV genome with adaptive mutations (Y835H in NS2, K1402Q in NS3, and V2440L in NS5A) and harboring H77 sequence derived from the BLd-H77peptide, as well as plasmid pFKi389-Venus-Jc1, termed as Jc1, (an intra-genotypic recombinant between J6-CF sequence (AF177036) and JFH1 sequence) were used to produce and electroporate into Huh7.5 the respective H77/JFH-1 and JC1 viral RNAs as described previously [21]. Huh7.5 cells, Huh7.5-BLd-tm or Huh7.5-C46 were infected with different dilutions of culture supernatants harvested at 24h, 48h and 72h post electroporation. Four days post-infection, cells were fixed with EtOH 100% and foci forming units (FFUs) were visualized after NS5A immunostaining as described previously [21]. For BLd-H77 dose-dependent neutralization assay, HCVcc particles were pre-incubated with different concentrations of BLd-H77 or with PBS for 1h at room temperature and were then used to infect Huh7.5. For time-course neutralization assay, Huh7.5 were infected with HCVcc for 4h prior washing. PBS or BLd-H77 (35 μg/ml) were added into cell supernatant for 1h prior infection, during infection or after infection. FFU/ ml were determined 4 days post-infection. To construct HCVcc particles harboring 2b1, J6-1/2, 2b1-1/2 or 2b1-1/3 envelope, we inserted by molecular cloning the related envelope into the pFKi389-Venus-Jc1 molecular clone, that initially encodes for J6 envelope. Viral RNAs were electroporated into Huh7.5 as described above. At 72h post electroporation, cell culture supernatants were tittered and used to infect naïve Huh7.5. Number of foci forming units per ml were determined 4 days post infection as described above. In parallel, viral RNA were extracted from electroporated cell culture supernatants at 72h post (ZR viral RNA kit, Zymo). HCV viral RNA copy number was quantified by one-step reverse transcription-PCR (RT-PCR) using *MultiCode*-RTx Real-Time PCR (Luminex) according to manufacturer’instructions and run on a Step One Plus quantitative PCR machine (Life Technologies). Data were analyzed using the MultiCode Analysis Software v1.6.5 (Luminex). The following primers were used for the detection of HCV RNA: GCTCACGGACCTTTCA (sense) and GGCTCCATCTTAGCCC (antisense).

### Huh7.5-BLd-tm infection assay

H77/JFH1 virus was used to infect Huh7.5 and Huh7.5-BLd-tm (m.o.i. 0,1). At day 1, 3 and 5 post infection, cells were fixed with 2% formaldehyde for 20 min at room temperature, washed and permeabilized with Perm/Wash Buffer (BD Biosciences) for 15 min at 4°C. NS5A expression levels were then quantified using anti-NS5A antibody 9E10 by flow cytometry (FACS CANTO II–BD Biosciences). In parallel, cell supernatants were harvested at each time point, filtered and used to infect naïve Huh7.5. 72h post infections, infectious titers (FFU/ml) of cell supernatant were determined as described above.

### Infection-dilution assay

HCVpp H77 and H77/JFH-1 HCVcc particles were preincubated for 1h at room temperature with PBS or BLd-H77 (50 μg/ml or 35 μg/ml respectively). Then, BLd-H77 and concentrated viral particles were diluted (1/5) with cell culture media or not prior infection of Huh7.5. 4 days post infection, percentages of GFP-positive cells were determined by flow cytometry and FFUs/ml were determined by NS5A immunostaining as described above.

### Cell-to-cell transmission assay

H77/JFH-1 were used to infect Huh7.5 for 4h at 37°C (m.o.i. 0,05). After washing, cells were incubated with anti-E2 antibody AP33 (25μg/ml) alone, or mixed with BLd-H77 (35μg/ml), or with PBS. 72h post infection, cells were fixed and numbers of cell per foci for each condition were quantified through NS5A immunostaining as described above.

### PHH infection and neutralization assay

PHH were washed and infected by JC1 HCVcc virus at different m.o.i. (0,005; 0,001; 0,05; 0,1). 4 days post infection, cell culture supernatants were harvested and used to infect naïve Huh7.5. Infectious titers were revealed through NS5A immunostaining 4 days post infection as described above. Indirect titrations were performed as infected PHH were poorly detectable through NS5A immunostaining, making the indirect titration the only accurate method to quantify the amount of infectious viral particles (and not physical viral particles) in a PHH-cell culture supernatant. For neutralization assay, JC1 virus or JC1-derived HCVpc were pre-incubated with BLd-H77 (10, 20 and 40 μg/ml) or with PBS for 1h prior PHH (m.o.i. 0,05) or Huh7.5 infection respectively (m.o.i. 0,01 and 0,02). 4 days post infection, infectious titers of PHH cell culture supernatants were determined by infecting Huh7.5 as described above. In parallel, infectious titers of JC1-derived HCVpc following Huh7.5 infection were quantified as described above. Sera containing HCV particles were used to infect PHH at a m.o.i. of 0,1 following BLd-H77 (30 μg/ml) or PBS preincubation for 1h at room temperature. 4 days post infection, cell culture supernatants were harvested and viral loads (RNA copies number/ml) were determined by RT-qPCR using a clinical diagnostic kit (Abbot Real Time™ HCV assay) with a limit of quantification (LOQ) of 12 IU/mL (i.e. 51.6 HCV RNA copies/ml). Given a serum dilution of 1:100 in PBS, LOQ = 5160 HCV RNA copies/ml.

### Establishment of BRL cell lines expressing human CD81 and human SR-BI

Retroviral vectors expressing human CD81 (NM_004356) and SR-BI (Z22555) were described previously [52]. Retroviral vectors containing these cDNAs were produced from 293T cells as VSV-G pseudotyped particles as described previously [53,54]. Stable expression of either receptor in BRL cells was obtained as described previously [52].

### E2 and HCVpp binding assay

Binding assays were performed as described previously [21]. Briefly, 50μl of concentrated virus (100x) or 100 ul of concentrated soluble E2 (100x) were pre-incubated with BLd-H77 (50 μg/ml) or with PBS for 1h at room temperature. Then, pseudoparticles or soluble E2 were mixed with Huh7.5, BRL, BRL-CD81 or BRL-human SR-BI in presence of 0.1% sodium azide for 1h at 37°C. Cells were then washed with PBFA (PBS, 2% fetal bovine serum, and 0.1% sodium azide). Bound viruses were detected using the mouse H53 anti-HCV E2 antibody and soluble E2 was detected using either the H53 antibody or a rabbit 6His-tag antibody for 1h at 4°C. After washing, primary antibodies were quantified by flow cytometry (FACS Canto II, BD Biosciences) using APC goat anti-mouse immunoglobulin-G. In parallel, concentrated HCVpp were pre-incubated with 50 μg/ml of BLd-H77 and used to infect Huh7.5 in order to verify the neutralizing effect of BLd-H77 on the entry of concentrated HCVpp. For BLd-H77 binding assay, BLd-H77 (50 μg/ml) or PBS were mixed with Huh7.5 for 1h at 37°C prior 6His-tag staining using a rabbit 6His-tag antibody.

### Binding enhancement assay

Concentrated soluble E2 (100x; 100 or 250 μl) were mixed with equivalent number of Huh7.5 or Huh7.5-BLd-tm (2x10^5^ or 5x10^5^ cells) in presence of 0.1% sodium azide for 1h at 37°C. After washing, bound soluble E2 were detected using anti-E2 H53 antibody as described above. Levels of binding enhancement were determined relatively to the basal E2 binding on naïve Huh7.5. For HCVpp binding enhancement assay, 1x10^5^ Huh7.5 cells or Huh7.5-BLd-tm were infected with HCVpp for 4 hours. Cells were then trypsinized and washed with PBFA (PBS, 2% fetal bovine serum, and 0.1% sodium azide). Bound viruses were quantified by flow cytometry (FACS Canto II, BD Biosciences) following cell staining with the mouse H53 anti-HCV E2 and an APC goat anti-mouse immunoglobulin-G.

### HCVcc binding assay

JC1 HCVcc particles (5x10^4^ i.u.) were pre-incubated with BLd-H77 (35 μg/ml), Heparine (250 μg/ml) or with PBS for one hour at 37°C. Viral particles were then mixed with 1x10^5^ Huh7.5 cells for 2h at 4°C. After 3 washings, cells were lysed using the RLT Buffer (QIAGEN) complemented with β-mercaptoethanol. Total RNAs were then extracted using the RNeasy mini kit (QIAGEN) as recommended by the manufacturer. Extracted RNAs were reverse transcribed using the iScript cDNA synthesis kit (Bio-Rad) and HCV viral RNA (5-CTTCACGCAGAAAGCGCCTA and 5-CAAGCGCCCTATCAGGCAGT) and human GAPDH (5- GAAGGTGAAGGTCGGAGTC and 5- GAAGATGGTGATGGGATTTC) were then quantified by qPCR using the FastStart Universal SYBR Green Master kit (Roche Applied Science) on an Step One Plus quantitative PCR machine (Life Technologies). Cell-associated viral RNA copies were normalized on human GAPDH expression for each sample.

### ELISA assay

96-well plates (Corning) were coated overnight with different amounts of a mouse IgG isotype (10ng and 100ng; Abcam), anti-E2 antibody AR3B [32] (10 and 100ng), and BLd-H77 (10, 100 and 250ng). The next day, following a one-hour incubation step with a SuperBlock blocking buffer (Thermo Scientific) to prevent non-specific binding, each coating condition was incubated with 10ng of sE2 or not. Interactions were revealed using a rat anti-E2 antibody 3/11 [47] or a rat IgG isotype, then followed by an incubation with an anti-rat HRP antibody (Biorad). Optical density signals at 450 nm were then assessed using a TriStar Multimode Microplate reader (Berthold).

### Mice and primary human hepatocytes (PHH) transplantation

FRG (Fah^–/–^Rag2^–/–^Il2rg^–/–^) mice (mixed background: C57BL/6 and 129Sv) were housed in our animal facility (Plateau de Biologie Experimentale de la Souris, PBES, Lyon, France). Because of their lethal phenotype, mice are maintained on 8 mg/l of NTBC (nitro-4-trifluoro-methylbenzoylcyclohexanedione) in the drinking water. 48h prior the engraftment, adult (6–10 weeks old) mice were injected intravenously with 2x10^9^ p.f.u. of an adenoviral vector encoding the uPA transgene. 7x10^5^ to 1x10^6^ PHH (BD Biosciences) were injected intrasplenically as previously described [55]. Immediately after engraftment, the NTBC was progressively withdrawn as follow: 2 days at 10% of colony maintenance concentration, then 2 days at 5%, 2 days at 2.5%, then the NTBC was completely removed. During the phase without NTBC, mice were weighted every two days. After 2–6 weeks, mice with clinical symptoms (lethargy, hunched posture) or severe weight loss (>15%) were put again on NTBC for 3 days before second withdrawal (cycling). Cycling was repeated until clinical symptoms resolved. In order to prevent the development of a murine hepatocellular carcinoma, highly reconstituted mice selected for infectious experiments were subjected to further NTBC treatment (3–4 weeks w/o NTBC and 3 days with 100%).

### FRG mice serological analyses

Blood from transplanted mice and controls were collected every 2–4 weeks after engraftment by retro orbital puncture. Sera were sent to a diagnostic laboratory for quantification of human Albumin (Cobas C501 analyzer, ROCHE).

### HCV infection of humanized liver FRG mice

Highly reconstituted mice (HSA >15mg/ml) were infected with JC1 HCVcc particles inocula (10^5^ i.u.) or with patient-derived HCV particles (5x10^4^ i.u.) via intraperitoneal route. Mice sera were collected at day 7 and day 14 post-infection by retro-orbital bleeding. At day 21, mice were sacrificed, sera were collected and levels of human albumin were determined (Cobas C501 analyzer, ROCHE). JC1 infectious titers in mice sera were determined through infection of Huh7.5 with different dilutions of sera as described above. In parallel, for HCV particle containing sera, viral load was determined by RT-qPCR using a clinical diagnostic kit (Abbot Real Time™ HCV assay) with a limit of quantification (LOQ) of 12 IU/mL (i.e 51.6 HCV RNA copies/ml). Given a serum dilution of 1:100 in PBS, LOQ = 5160 HCV RNA copies/ml.

### *In vivo* HCV inhibition assay

A first cohort of 11 mice was attributed for an *in vivo* inhibition assay of JC1 infection (9 mice + 2 negative controls; one non-infected and one non-engrafted). 7 mice were treated under a prophylactic protocol with PBS (4 mice) or with 30 μg (3 mice) of BLd-H77. Mice were treated via intra-peritoneal route one day prior infection, and at day 1, 7 and/or 14 post-infection infection. For all the mice, sera were harvested and viral titers were quantified at day 7, 14 and during sacrifice 21 post infection. A second independent cohort of 7 mice was attributed for another *in vivo* inhibition assay of JC1 infection (5 mice + 2 negative control). Here, JC1 infection was challenged with a dose of 150μg of BLd-H77 (2 mice) or with PBS (3 mice) under a prophylactic protocol similar as described above. A cohort of 14 mice (12 mice + 2 negative control) was attributed for an *in vivo* inhibition assay of serum-derived HCV particles infection, challenged with one dose of BLd-H77 (150 μg, 5 mice) or with PBS (7 mice) under a prophylactic protocol similarly to what has been described above. Serum-derived HCV particles viral load were determined as described above. Following sacrifice, the level of human Albumin was quantified for each untreated and treated mice in order to ensure that HCV infection inhibition was not due to a decrease of liver humanization caused by BLd-H77.

### Cell-cell fusion assay

Cell-cell fusion assays were performed as described previously [13,19]. Briefly, HEK-293T cells (2.5x10^5^ cells/well seeded in six-well tissue culture dishes 24 h before transfection) were co-transfected using calcium phosphate reagent with a HCV (H77, J6, 2b1 or J6/2b1 chimera) or VSV-G envelope encoding-plasmids and with an HIV-1 LTR (long terminal repeat) luciferase reporter plasmid. After 12h, transfected HEK-293T cells were detached with versene (0.53 mM EDTA; Invitrogen) and co-cultured (5x10^4^ cells/well) with Huh-7-Tat indicator cells (5.10^4^cells/well) in a 24-well plate. After 24 h, the cells were washed with serum free DMEM, incubated for 3 min in either pH 7 or pH 5 buffer (130 mM NaCl, 15 mM sodium citrate, 10 mM Mes and 5 mM Hepes) and then washed three times with serum free DMEM. The luciferase activity was measured 72 h later using a luciferase assay kit according to the manufacturer’s instructions (Promega). For fusion neutralization assay, coculture were washed, pre-incubated with BLd-H77 (50μg/ml) or with PBS for 1h at 37°C prior a second washing and exposure to pH buffer. Alternatively, co-cultured cells pre-treated by BLd-H77 or PBS were incubated for an extension of 24h following pH shock by BLd-H77 (50μg/ml) or PBS.

### Liposomes fusion assays

HCVpp/liposome lipid mixing was performed as previously described [21,56]. R18-labeled liposomes were obtained by mixing octa-decyl rhodamine B chloride (R18; Molecular Probes) and lipids (phosphatidylcholine and cholesterol; Aventi) and mixed with 40 μl of concentrated HCV pseudoparticles (non-enveloped HCVpp, HCVpp-H77 and HCVpp harboring the fusion defective envelope E140/E2H77 previously characterized [21]) or retroviral particles pseudotyped with Influenza Hemagglutinin-Neuraminidase (HANApp), all diluted in PBS (pH 7.4) within a 37°C thermostable 96-well plate. After pH decrease to 5 (acidification), dequenching of R18 due to lipid mixing between HCVpp and liposomes were recorded on a micro-plate fluorometer (InfiniteM1000 Tecan Group Ltd) for a period of 5 to 20min with an excitation wavelength (λexc) at 560 nm and an emission wavelength (λem) at 590 nm. Maximal R18 quenching was measured after the disruption of liposomes by the addition of 0.1% TritonX-100. For fusion-neutralization assays, pseudoparticles were pre-incubated with different doses of BLd-H77 or with PBS for 1h prior incubation with liposomes and acidification.

### Structural analysis

Structural analysis of Dengue E pre-fusion structure (PDB 1K4R), E-Pr complex structure (3C6E), E post-fusion structure (1OK8) and E2core structure (4MWF) were realized using Chimera software [57] (UCSF).

### Statistical analysis

GraphPad Prism software (version 6) was used for statistical analysis. Statistics were calculated using Student’ t test and/or two-way ANOVA when appropriate. (*p<0.05, **p<0.01, ***p<0.001).

### Protein sequence alignment

Protein amino acid sequence alignments were realized using ClusterX2.1 and rendered as Post-script format.

### Computational coevolution analysis using BIS

The BIS methodology and related-coevolution signal analysis are described in details in reference [22], [23] and [24]. Applicability of BIS for detecting coevolution signals in viral sequences is specifically described in reference [23].

### Attribution of putative functions to E1E2 clusters in S4 Table and S7 Table

Literature describing amino acids mutations within E1E2 sequences impacting E1E2 folding/heterodimerization, E1E2 binding to cellular receptors or E1E2 fusion was used as references to attribute functions to clusters mapping specific regions within E1E2 sequences. The references used were the following: Folding/Heterodimerization [19,27,28,58–60], Viral binding site conformation [19,20,26,60–66] and Fusion [11,13,19–21,27,67].

## Supporting information

S1 Text Detailed analysis of genotype 1a HCV E1E2 clusters. (DOCX)

S2 Text Detailed analysis of genotype 2 HCV E1E2 clusters. (DOCX)

S1 Table Clusters of coevolving residues identified by BIS in DENV envelope glycoprotein E and PrM sequences of serotype 2. 17 amino acids sequences of DENV E and PrM serotype 2 were aligned and 14 Clusters were identified by BIS. Clusters are computed with the BIS coevolution analysis method [22–24] and they correspond to maximum scores (symmetricity and environmental scores are set to 1, and the number of admissible exceptions to 0 or 1). For each cluster, the positions of the different coevolving residues or blocks (the initial and final position of each block is reported), and corresponding p-value, are indicated. BIS considered the first amino-acid of PrM as position 1 for all the analyzed sequences. For each cluster, the frequency of its most conserved residues is given (“conservation score”). It should be noted that in BIS, when scores are maximal (that is, set to 1 as for this analysis), all blocks/residues in a cluster display the same amino-acid distribution (See the identical distribution of residues for the two coevolving positions in the alignment of **S1 Fig** as an example).
(DOCX)

S2 Table Clusters of coevolving residues identified by BIS in DENV envelope glycoprotein E sequences of serotype 2. Clusters are computed with the BIS coevolution analysis method [22–24] and they correspond to maximum scores (symmetricity and environmental scores are set to 1, and the number of admissible exceptions to 0 or 1). For each cluster, the positions of the different coevolving residues or blocks (the initial and final position of each block is reported) and the corresponding p-value are indicated. BIS considered the first amino-acid of E as position 1 for all the analyzed sequences. For each cluster, the frequency of its most conserved residues is given (“conservation score”). It should be noted that in BIS, when scores are maximal (that is, set to 1 as for this analysis), all blocks/residues in a cluster display the same amino-acid distribution (See the identical distribution of residues for the two coevolving positions in the alignment of **S1 Fig** as an example).
(DOCX)

S3 Table BIS coevolution analysis of HCV E1E2 sequences. Ten groups of sequences were assembled and analyzed independently with the BIS method. Groups were constituted of E1E2 sequences from HCV types and sub-types from genotype 1a to 6a. Groups of sequences from genotypes 1 and 2 were constituted by pools of sequences from subtypes 1a and 1b (50 sequences) and sequences of genotypes 2a and 2b (30 sequences). Total numbers of detected clusters for each genotype and sub-type is reported, as well as the number of statistically significant clusters among them (when p<0.05). For each group of sequence, we also report the number of statistically significant clusters only involving E1 positions (“intra-E1”), the number of clusters only involving E2 positions (“intra-E2”), and the number of clusters across E1 and E2 (“inter-E1-E2”). The assignment of a given cluster block to E1 or E2 was determined by mapping the reference genome sequence of genotype 1b (accession: AJ238799) to the multiple sequence alignment, for each genotype. E1 and E2 were identified on AJ238799 at positions 192–383 and 384–746, respectively. Note however that residue positions displayed in **S4 Table**, **S7 Table** and in the related HCV webserver (http://www.lcqb.upmc.fr/HCVenv/HCVenv.html) are specific to each genotype and set of patient sequences analyzed.
(DOCX)

S4 Table Clusters of coevolving residues identified by BIS in HCV E1E2 sequences of genotype 1a. Clusters are computed with the BIS analysis method similarly to **S1 Table**. Note that residue positions displayed in this table are specific to the set of patient sequences analyzed. Hence, nucleotide gaps generated during the analysis of the patient sequences by BIS were taken into account when plotting gt1a clusters into a gt1a reference E1E2 reference (H77, AF009606; (**S4 Fig**) and into gt1a E2core structure (**S5 Fig**).
(DOCX)

S5 Table List of Genotype 1a cluster blocks mapped on E1E2 references sequences (H77, AF009606). For each block, the initial and final position of the block predicted by BIS and the name of the cluster it belongs to are given. Blocks from each cluster are numerated from 1 to x (Block N°) to easily identify their position on the E1E2 sequences in **S4 Fig**, where each block is referenced as follow: “Cluster ID-Block N°”.
(DOCX)

S6 Table Putative functions of genotype 1a E1E2 coevolution clusters. We aligned 25 E1E2 amino acids sequences of HCV genotype 1a and identified using BIS method 16 clusters (**S3 Fig; S4 Table**). Genotype 1a clusters harboring blocks that mapped residues previously reported in the literature to have a specific function (**S4 Fig**) are classified. The known role(s) are indicated: Folding or heterodimerization (blue), viral binding site conformation (green) or fusion mechanism (red). According to these roles, clusters are categorized into three different categories: structural (Folding, heterodimerization and viral binding site conformation), fusion or multifunctional cluster. Clusters harboring blocks that did not map any residues with a previously reported function were classified as clusters with “undefined role.
(DOCX)

S7 Table Clusters of coevolving residues identified by BIS in HCV E1E2 sequences of genotype 2. Clusters are computed with the BIS analysis method similarly to **S1 Table**. Note that residue positions displayed in this table are specific to the set of patient sequences analyzed. Hence, nucleotide gaps generated during the analysis of the patient sequences by BIS, as well as gaps between gt1a and gt2 sequences, were taken into account when plotting gt2 clusters into a gt2 reference E1E2 reference (JFH-1, AB047639; **S7 Fig**) and into gt1a E2core structure (**S8 Fig**).
(DOCX)

S8 Table List of genotype 2 cluster blocks mapped on the E1E2 reference sequences (JFH-1; AB047639). For each block, the initial and final position of the block predicted by BIS and the name of the cluster it belongs to are given. Blocks from each cluster are numerated from 1 to x (Block N°) to easily identify their position on the E1E2 sequences in **S7 Fig**, where each block is referenced as follow: “Cluster ID-Block N°”.
(DOCX)

S9 Table Putative functions of genotype 2 E1E2 coevolution clusters. We aligned 30 E1E2 amino acid sequences of HCV genotype 2 (2a and 2b) and, using the BIS method, we identified 21 clusters (**S6 Fig; S3, S7 Table**). Genotype 2 clusters harboring blocks that mapped residues previously reported in the literature to have a specific function are classified. The known role(s) of the residues in each cluster are indicated: Folding or heterodimerization (blue), viral binding site conformation (green) or fusion mechanism (red). According to these roles, clusters are categorized into three different categories: structural (Folding, heterodimerization and viral binding site conformation), fusion or multifunctional cluster. Clusters harboring blocks that did not map any residues with a previously reported function were classified as clusters with “undefined role”.
(DOCX)

S10 Table BIS analysis of the coevolution of the transmembranes of E1 and E2. List of clusters identified by BIS that connect the transmembrane of E1 and E2. Six clusters were identified across the two major HCV genotypes. Location of the transmembrane domains within E1 and E2 are indicated for each genotype and sub-types. Position of the coevolving blocks located within the transmembrane of E1 and E2 are indicated for each cluster. TMD, Transmembrane Domain. Only significant clusters are reported (p value≤0.05).
(DOCX)

S1 Fig BIS methodology for coevolution analysis. Coevolution analyses require the identification of sequence variability and they are usually realized on families of homologous protein sequences, typically very divergent and represented by a large number of sequences. In contrast, studying viral sequences requires methods that can analyze serotypes and genotypes, usually constituted by a limited number of conserved sequences. BIS allows to track small parallel changes likely corresponding to compensatory patterns preserving the structure and function of the protein. Given a sequence alignment, the coevolution analysis method Blocks In Sequences (BIS) [22–24] identifies groups of residues where mutations are present, at the same time, in the same sequences. BIS allows to track small parallel changes likely corresponding to compensatory patterns preserving the structure and function of the protein. BIS first detects coevolution among each pair of alignment positions (left) and associates a coevolution score to the pairs. At different colors match different coevolution scores (from red for high coevolution score to blue for none). A coevolution score matrix between all alignment positions is constructed (top right) and clustered in such a way that groups of positions displaying the same coevolution scores with all other positions in the alignment are identified (bottom right). The schema illustrates two clusters made of four blocks (cluster A and B). Each cluster is composed of blocks displaying a similar pattern of coevolution scores (here represented by a similar color pattern). Cluster A is constituted by six positions organized in four blocks, three of them corresponding to single residue positions and one of them made of three consecutive residue positions. Cluster B is made of four positions organized in four blocks, one of them corresponding to single residue positions and three of them to two or three consecutive residue positions. For both clusters, blocks belong to either E1 or E2 protein. An illustration of how these blocks can be displayed on a linear sequence (E1) or on a protein structure (E2core) is shown. This figure has been adapted from the figure 2 of the reference [23].
(TIF)

S2 Fig Structural mapping of DENV E clusters. Mapping of the DENV E clusters 3 to 12 (illustrated by distinct colors) on the E dimeric structure (PDB 1K4R). For each cluster, positions of the coevolving blocks in E sequence are displayed within “strips” located above each structure (See **S2 Table** for cluster positions). Each cluster is identified by a distinct color. Cluster 1 and 2 were too large to be considered. A linear representation of Dengue E protein is also shown at the top of the figure. Starting and ending residue positions of each E domain are indicated. E domains are annotated by distinct colors: DI, domain I (red); DII, domain II (yellow); DIII, domain III (blue); Tmd, transmembrane (black).
(TIF)

S3 Fig Coevolution analysis of HCV E1E2 genotype 1a sequences. The 16 gt1a clusters (illustrated by distinct colors) are displayed within “strips” representing the E1E2 sequence. For each cluster, positions of the coevolving blocks in the E1E2 sequence are indicated within the corresponding “strip” (see **S4 Table** for cluster positions). On the top of each coevolving block is indicated the corresponding protein or E2 domain the block belongs to: HVR1, Hyper Variable Region 1; FL, Front Layer; VR2, Variable Region 2; βS, β-Sandwich; BinL, CD81 Binding Loop; BL, Back Layer; Stem; Tmd, Transmembrane domain). Two small linear representations of HCV E1E2 are located at the top of each “strip” column, and can be used as reference for determining the position of each coevolving block within the E1E1 sequence. At the top of the figure is shown an enlarged linear representation of HCV E1E2, where the starting and ending residue positions of each protein and domain are indicated. E2 domains are highlighted by distinct colors (Green, BL; Red, central β-sandwich; Blue, front layer; Dark blue, CD81 BinL/CD81 binding loop; Light grey, central β-sandwich–back layer linker; Black dotted line, VR2/Variable Region 2; Grey dotted line, Stem; Black rectangle, Tmd/Transmembrane).
(TIF)

S4 Fig Mapping of genotype 1a cluster blocks on E1E2 reference sequences of genotype 1a (H77; AF009606) and genotype 2a (JFH-1; AB047639). Genotype 1a cluster blocks are represented as red horizontal bars positioned above the E1 and E2 H77 or JFH-1 aligned sequences. Each horizontal bar is numerated as follow: “Cluster ID-Block N°”. Respective positions of these blocks are referenced in **S5 Table**. Residues (boxes) or domains (horizontal arrows) previously identified in the literature to have a function are indicated (see Materials and Methods for references). A color code, reported below the sequence alignment, links residues (boxes) or domains (arrow) to specific functions according to the literature: blue for folding/heterodimerization, green for binding and red for fusion. Multi-colored code boxes represent residues with two identified functions. In E2, the AR3A antibody epitope is shown as three orange arrows. The putative function determined for each cluster is summarized in **S6 Table**. The addition of one amino acid on the left and on the right of each block is considered to take into account potential structural adjustments after mutation that are not considered in BIS calculation. Cluster blocks positions are numbered according to H77 gt1a (AF009606; **S4 Table**).
(TIF)

S5 Fig Structural analysis of genotype 1a HCV E1E2 clusters. HCV E1E2 gt1a multifunctional clusters 4 (blue) and 16 (orange) (**A**), structural clusters 11 (orange), 2 (green) and 6 (red) (**B**) and undefined role clusters 3 (pink), 9 (emerald green), 13 (brown), 14 (blue) and 15 (yellow) (**C**) are plotted on a vertical linear representation of E1 and on a tridimensional view of E2 core (PDB 4MWF). Each cluster is composed of blocks harboring a similar color, according to **S3 Fig**. The Stem region (Stem) is represented by a dotted line after the C-terminal part of the BL. The transmembrane domain (Tmd) is represented as a rectangle following the Stem region. In **B** and **C**, bold lines link E1 and E2 blocks that coevolve. For each cluster, block positions in E1 (at the left of the linear structure) and E2 (below boxes whose color match the color of the corresponding cluster) are indicated. In **A**, the E2 central scaffold is enlarged and red circles highlight regions where cluster 4 blocks are in close proximity. Rotation angles of the E2core structure are indicated. Viewing angle of E2core is indicated by a black cross (Reference: **Fig 3B**). FL, Front Layer; BinL, CD81 Binding Loop; VR2, Variable Region 2; BL, Back Layer; βS, β-Sandwich.
(TIF)

S6 Fig Genotype 2 HCV E1E2 coevolution networks. The 21 gt2 clusters (illustrated by distinct colors) are displayed within “strips” representing the E1E2 sequence. For each cluster, positions of the coevolving blocks in the E1E2 sequence are indicated within the corresponding “strip” (see **S7 Table** for cluster positions). On the top of each coevolving block is indicated the corresponding protein or E2 domain the block belongs to: HVR1, Hyper Variable Region 1; FL, Front Layer; VR2, Variable Region 2; βS, β-Sandwich; BinL, CD81 Binding Loop; BL, Back Layer; Stem; Tmd, Transmembrane domain). Two small linear representations of HCV E1E2 are located at the top of each “strip” column, and can be used as reference for determining the position of each coevolving block within the E1E1 sequence. At the top of the figure is shown an enlarged linear representation of HCV E1E2, where the starting and ending residue positions of each protein and domain are indicated. E2 domains are highlighted by distinct colors (Green, BL; Red, central β-sandwich; Blue, front layer; Dark blue, CD81 BinL/CD81 binding loop; Light grey, central β-sandwich–back layer linker; Black dotted line, VR2/Variable Region 2; Grey dotted line, Stem; Black rectangle, Tmd/Transmembrane).
(TIF)

S7 Fig Mapping of genotype 2 cluster blocks on E1E2 reference sequences of genotype 1a (H77; AF009606) and genotype 2a (JFH-1; AB047639). Genotype 2 cluster blocks are represented as blue horizontal bars positioned above the E1 and E2 H77 or JFH-1 aligned sequences. Each horizontal bar is numerated as follow: “Cluster number-Block N°”. Respective positions of these blocks are referenced in **S8 Table**. Residues (boxes) or domains (horizontal arrows) previously identified in the literature to have a function are indicated (see Online Methods for references). A color code, reported below the sequence alignment, links residues (boxes) or domains (arrow) to specific functions according to the literature: blue for folding/heterodimerization, green for binding and red for fusion. Multi-colored code boxes represent residues with two identified functions. In E2, the AR3A antibody epitope is shown as three orange arrows. The putative function determined for each cluster is summarized in **S9 Table**. The addition of one amino acid on the left and on the right of each block is considered to take into account potential structural adjustments after mutation that are not considered in BIS calculation. Cluster blocks positions are numbered according to JFH-1 gt2 (AB047639; **S7 Table**).
(TIF)

S8 Fig Structural analysis of the genotype 2 HCV E1E2 clusters. (**A**) Plot of the gt2 structural cluster 19 on a tridimensional view of the E2core structure (PDB 4MWF). Gt2 structural (**B**), multifunctional (**C**), structural and multifunction (**D**), fusion (**E**) and undefined (**F**) clusters were plotted both on a vertical linear representation of E1 and onto the E2 core structure. Each cluster is composed of blocks harboring a similar color, according to **S6 Fig**. The Stem region (Stem) is represented by a dotted line after the C-terminal part of the BL. The transmembrane domain (Tmd) is represented as a rectangle following the Stem region. In (**B**) and (**C,E,F**), bold lines link E1 and E2 blocks that coevolved. For each cluster, block positions in E1 (at the left of the linear structure) and E2 (below boxes whose color match the color of the corresponding cluster) are indicated. Inter-E1E2 interactions mediated by gt2 structural clusters 7, 8, 11 and intra-E2 interactions mediated by gt2 structural cluster 18 and 21 are represented in **B,** while multifunctional clusters 5 and 12 are represented in (**C**). Areas of close proximity between blocks in cluster 19 (**A**) and between blocks in cluster 11 (**D**) and 12 (**D**) are enlarged and highlighted by red circles. Panel **E** shows gt2 fusion clusters 6, 16 and 17. Panel **F** shows gt2 undefined role clusters 3, 4, 9, 14, 15 and 20. Rotation angles of the E2core structure are indicated. Viewing angle of E2core is indicated by a black cross (Reference: **Fig 3B**). HVR1, Hyper Variable Region 1, FL, Front Layer; BinL, CD81 Binding Loop; BL, Back Layer, βS, β-sandwich.
(TIF)

S9 Fig Structural analysis of genotype 3 clusters involving E1 and the BL. Genotype 3 cluster 4 (blue), cluster 8 (red) and cluster 9 (green) are plotted on a tridimensional view of the E2core structure (PDB 4MWF) and on a vertical linear representation of E1. Each cluster is composed of blocks harboring a similar color. The Stem region (Stem) is represented by a dotted line after the C-terminal part of the BL. The transmembrane domain (Tmd) is represented as a rectangle following the Stem region. The BL region is highlighted with a dotted circle on the E2core structure and enlarged alone at the right of the E1 linear representation. Bold lines link E1 and E2 blocks that coevolved. For each cluster, block positions in E1 (at the left of the linear structure) and E2 (below boxes whose color match the color of the corresponding cluster) are indicated. Rotation angles of the E2core structure are indicated. Viewing angle of E2core is indicated by a black cross (Reference: **Fig 3B**). Genotype 3 cluster positions are available through the following webpage: http://www.lcqb.upmc.fr/HCVenv/HCVenv.html/.
(TIF)

S10 Fig Biophysical properties and ELISA detection of soluble BLd-H77. (**A**) Amino acid sequence of the soluble BLd-H77. Dotted lines represent internal disulfide bridges that might be involved in the functional folding of the soluble BLd-H77. (**B**) Detection of BLd-H77 in reducing and non-reducing condition following SDS-Page electrophoresis and coomassie blue staining. (**C**) Size distribution profiles as a function of the intensity and volume of soluble BLd-H77, by Dynamic Light Scattering. The graph shows the superposition of three successive measurements from 12 runs at each concentration, which are representative for at least three independent experiments. (**D**) Far UV circular dichroism spectrum of soluble BLd-H77 after purification by size exclusion chromatography.
(TIF)

S11 Fig BLd-H77 inhibits HCV infection. (**A**) Quantification of HCVcc infectious titers following dose-dependent neutralization of HCVcc H77/JFH-1 by BLd-H77. Four days following BLd-H77 dose-dependent neutralization of HCVcc H77/JFH-1 infection (see **Fig 4F**), Huh7.5 cell culture supernatants were harvested and used to infect naïve Huh7.5 cells. Four days post-secondary infection, viral titers were determined by NS5A immunostaining (mean ± SD; n = 3). (**B**) Average IC50 of BLd-H77 inhibition (μg/ml) for HCVpp or HCVcc particles of different genotypes or sub-types. ND, not determined. (**C**) Bafilomycin A1 and AR4A mediated-inhibition of HCV entry. Huh7.5 cells (cells) or HCVpp (HCVpp-H77, virus) were incubated 1h with Bafilomycin A1 (20nM) or AR4A (25μg/ml) prior infection (2), during the 4h infection (3) or following infection (4). As control, cells were incubated at each step with equivalent volume of PBS (1). Percentages of infection were calculated 72 post infection based on viral titer obtained from control conditions. (mean ± SD; n = 3). Statistical significances (*p<0.05, ***p<0.001, ns non-significant) were determined for each experimental condition versus control condition (100%). N.A., non applicable, as significant cell death was observed for this experimental condition. (**D**) HCV receptors expression is not impaired by BLd-H77. CD81, SR-BI, Claudin-1 and Occludin expression in Huh7.5 cells pre-incubated overnight with PBS (upper line) or BLd-H77 (50μg/ml; bottom line). Following PBS or BLd-H77 pre-incubation, cells were fixed, permeabilized (only for Occludin staining) and stained for the different HCV receptors with (white areas) or without (grey areas) receptor-specific antibody. Expression levels were then determined by flow cytometry. Data are representative of two independent experiments. (**E**) BLd-H77 inhibition of cell-to-cell transmission. After HCVcc H77/JFH-1 infection, cells were pre-incubated with PBS (control), with AP33 antibody alone (25μg/ml) or with AP33 mixed with BLd-H77 (35μg/ml). Average numbers of cells per foci were determined by immunostaining for each condition 4 days post infection. **p<0.01, ns non-significant. (**F**) Titration of primary human hepatocytes (PHH) cell culture media following HCVcc JC1 virus infection. PHH were infected with HCVcc JC1 virus at different m.o.i. 4 days post-infection, PHH cell culture supernatants were used to infect Huh7.5 cells. Viral titers were determined 4 days post-secondary infection via NS5A immunostaining (mean ± SD; n = 3). (**G**) BLd-H77 mediated-inhibition of patient-derived HCV particles *in vivo*. Patient-derived HCV particles were pre-incubated with PBS or BLd-H77 (30μg/ml) and were used to infect primary human hepatocytes (m.o.i. 0.1). Four days post-infection, cell culture supernatants were harvested and viral load from primary infection were determined using a clinical diagnostic kit. Limit of detection (l.o.d.) of the kit is indicated by a dotted line (n = 1 due to the limited amount of patient sera available). (**H**) Serum-derived HCV viral loads from a mouse cohort treated with 150μg of BLd-H77 (blue square, n = 5) or with PBS (red circle, n = 7) under a “prophylactic” protocol. BLd-H77 or PBS was injected one day prior virus infection with patient serum as conducted in **Fig 4L**. Serum-derived HCV particle viral loads were determined as described in online methods. The limit of detection (l.o.d.) is indicated within the graph (dotted line). Viral loads below the dotted line represent non-quantifiable viral loads. One non-engrafted liver mice and one non-infected mice were used as negative controls (green, n = 2). A statistical analysis was realized on all quantifiable viral loads for all time points (p = 0.02). (**I**) Serum albumin concentration in humanized liver FRG mice prior infection and (Pre-infection) at the time of sacrifice (Week 3). Albumin concentration were determined in the context of HCVcc JC1 (JC1) and serum-derived HCV infection (Serum), and with BLd-H77 treatment (BLd) or not (PBS). Medians are shown for each experimental group. (5–7 mice per group). ns non-significant.
(TIF)

S12 Fig BLd-H77 transmembranous form inhibits HCV infection. (**A**) Cell surface staining of C46 and BLd-tm following transduction of Huh7.5. Expression of C46 and BLd-tm (white) was measured by flow cytometry using an anti-hinge human IgG2 antibody and compared to the level of expression within non-transduced Huh7.5 (Grey). (**B**) CD81, SR-BI, Claudin-1 and Occludin expression within Huh7.5 (left column) and Huh7.5 expressing the transmembranous form of the BLd-H77 (Huh7.5-BLd-tm) (right column) cells. Cells were fixed, permeabilized (only for Occludin staining) and stained for the different HCV receptors with (white areas) or without (grey areas) receptor-specific antibody. Expression levels were then determined by flow cytometry. Data are representative of two independent experiments. (**C**) Effect of BLd-tm expression on cell-to-cell viral spread. Huh7.5, Huh7.5-C46, Huh7.5-BLd-tm cells were infected by H77/JFH-1 HCVcc. Four days post-infection, average number of cells per foci were determined for each cell type (mean ± SD; n = 3). ***p<0.001, ns non-significant. (**D**) Retention of HCVpp particles at the Huh7.5-BLd-tm cell surface. Huh7.5 (left) and Huh7.5-BLd-tm (right) were infected with H77 HCVpp particles. Presence of HCV E2 at the cell surface was quantified by flow cytometry using anti-E2 H53 antibody. Gating were set up on non-infected Huh7.5 (left) and Huh7.5-BLd-tm (right). Data are representative of two independent experiments. (**E**) Interaction between BLd-H77 and sE2 detected by ELISA. Different amounts of mouse IgG isotype, AR3B and BLd-H77 were coated overnight into 96-well plates. Coated peptides and antibodies were then incubated with 10ng of soluble E2 (sE2). After washing, soluble E2 was detected using the anti-E2 antibody 3/11 or a rat isotype IgG antibody. Following incubation with an anti-rat HRP antibody, 3/11 binding specificity to E2 was determined by calculating the ratio of O.D. between condition using 3/11 antibody and the rat isotype IgG antibody, for each coating condition (mean ± SD; n = 3). ****p<0.0001, ns non-significant.
(TIF)

S13 Fig Effect of BLd-H77 on E2 and HCV pseudoparticles binding. (**A**) BLd-H77 effect on E2 and HCVpp binding to Huh7.5 cells (Left). Huh7.5 cells, pre-incubated with PBS (top panels) or BLd-H77 (50μg/ml) (bottom panels), were mixed (white) or not (grey), at 37°C for 1h with soluble E2 or with concentrated H77-HCVpp. Bound soluble E2 or HCVpp were then stained with an anti-6his tag antibody or an anti-E2 antibody, respectively. Binding was then quantified by flow cytometry following incubation with appropriate APC antibody. Data are representative of two independent experiments. Ability of BLd-H77 to bind to Huh7.5 cell membrane (Right). BLd-H77 (50μg/ml, white) or PBS (grey) was incubated for 1h at 37°C with Huh7.5 cells. Absence of bound BLd-H77 at Huh7.5 cell surface was verified by flow cytometry following staining using an anti-6his antibody or an anti-E2 antibody as control. (**B**) Expression of CD81 and SR-BI at BRL cell surface and HCVpp binding. BRL cell lines were transduced by vectors encoding human CD81 or SR-BI (hSR-BI). After selection, naïve BRL (grey) or transduced-BRL (white) cells were stained by anti-CD81 or anti-hSR-BI and by the appropriate secondary APC antibody. Quantification of CD81 and hSR-BI expression was determined by flow cytometry. (**C**) BLd-H77 effect on E2 and HCVpp binding to CD81 and hSR-BI. Naïve BRL (grey) or HCV-receptor expressing BRL (white) cells were mixed with concentrated H77-HCVpp pre-incubated with PBS (top panels) or BLd-H77 (50μg/ml) (bottom panels). Bound HCVpp were then stained with an anti-E2 antibody and with an appropriate APC antibody. Binding was then quantified by flow cytometry following incubation. Data are representative of three independent experiments. (**D**) Relative quantification of HCVpp binding to BRL cells expressing exogenous hCD81 or hSR-BI. Binding of HCVpp is presented as percentage of binding, relatively to the binding efficiency of HCVpp pre-incubated with PBS. (mean ± SD; n = 3). Statistical significances (*p<0.05, ns non-significant) were determined for each experimental condition versus control condition (100%). (**E**) Competitive entry inhibition assay using BLd-H77 and a neutralizing anti-E1E2 antibody. HCVpp-H77 were incubated for 1h at room temperature with BLd-tm (35 or 50 μg/ml), AR4A antibody (2 or 17 μg/ml) or with both protein (35 and 2 μg/ml or 35 and 17 μg/ml), and used to infect Huh7.5. Percentage of infection were quantified 72h post infection and calculated according to viral titer obtained for control conditions (mean ± SD; n = 3). *p<0.05, **p<0.01.
(TIF)

S14 Fig Effect of BLd-H77 on cell-cell fusion. (**A**) LTRhiv-luciferase vector transduced 293T cells expressing H77 HCV (**left panel**) or VSV (**right panel**) envelope glycoproteins were co-cultured with Tat-expressing Huh7.5 cells. Co-culture were pre-incubated for 1h with 50μg/ml of BLd-H77 or PBS, washed and incubated for 3 min with a pH7 (red) or pH5 (orange) buffer. Luciferase activities were determined 72h post-exposure. Results are presented in relative light units (RLU) for each experimental condition (mean ± SD; n = 3). *p<0.05, **p<0.01. Statistical significances represent the significant differences between pH5 and pH7 condition, for each viral envelope and treatment (PBS or BLd-H77). (**B**) Inhibitory effect of the BLd-H77 prior and following pH shock. Co-cultures were either pre-incubated with BLd-H77 (50μg/ml, 2) for 1h prior pH shock (pH5), for 24h following pH shock (50μg/ml, 3) or both (4). As a control, co-cultures were pre-incubated with PBS for 1h prior pH shock, followed by an additional 24h incubation (1). 24h later, cells were washed and incubated for 48h. Relative fusion efficiency at pH5 between control (pre- and post-treated by PBS) and the different BLd-H77 incubation periods are indicated (mean ± SD; n = 3). Statistical significances (*p<0.05, ***p<0.001, ns non-significant) were determined for each experimental condition versus control condition (100%). (**C**) Specificity of HCVpp-liposome fusion assay. No enveloped HCVpp, H77 HCVpp, fusion mutant E1E2 HCVpp particles and HANApp particles (Retroviral particles pseudotyped with Influenza Hemagglutinin-Neuraminidase) were mixed with R18-labelled liposomes. Dequenching of R18 was quantified following sample acidification (pH5) over 5 minutes (300 seconds). Data are represented as non-linear polynomial fitted curves and display the evolution of the fusion rate from the time of acidification, relatively to the HCVpp-H77 fusion rate which has been normalized at 100% at 300s post acidification. Curves are representative of three independent experiments.
(TIF)

S15 Fig Functional linkage of E1 and BL coevolving residues identified by BIS. (**A**) Protein sequence alignment of E1E2 J6 and 2b1. Level of conservation for each amino acid position is indicated. Position of the coevolving blocks that belong to gt2 fusion cluster 5 are indicated by red rectangles. Position of the interchanged amino acid regions between J6 and 2b1 for the construction of the J6/2b1 chimeric envelope are shown (Region 1, 2 and 3). (**B**) Structural location of the E2 gt2 cluster 5 block (orange) and of the five J6/2b1 amino acids differences (blue, 1 to 5) within the E2 BL. A dotted line symbolizes the rational experimental dissection of the BL into two distinct sub-domains, noted region 2 (non-β sheet region) and region 3 (β sheet region), based on the distinct rearrangement of these two sub-domains. The distinct structural contexts of the mutation 1–2 (region 2, left) and of the mutation 3–5 (region 3, right) are highlighted. (**C**) J6/2b1 E2 chimera expression and incorporation onto HCVpp. Expression in transfected 293T cells (Cell lysates) and incorporation onto concentrated pseudoparticles (Viral Pellets) of E2 from J6, 2b1 and J6/2b1 chimera. Detection of E2 on pseudoparticles harboring no envelope glycoproteins was used as negative control. MLV-Capsid (CA) was detected to control equivalent HCVpp production between chimera. (**D**) Quantification of HCV viral RNA in the supernatant of Huh7.5 cell electroporated with viral RNA coding for HCVcc harboring different J6/2b1 envelope chimera. Cell culture supernatant were harvested at 48h and 72h post electroporation and viral RNA copy numbers per ml were determined by RT-qPCR for each viral strain (mean ± SD; n = 4). *p<0.05, **p<0.01.
(TIF)

S16 Fig Construction of E1-E2 genotype 1a chimera to challenge BIS predictions. Protein sequence alignment of E1E2 H77 and A40. Level of conservation for each amino acid position is indicated. Position of the E1 and E2 block of interest belonging to the gt1a cluster 5 are indicated by red asterisks. Position of the three H77 amino acid residues that will be replaced by A40 residues for the construction of H77/A40 chimeric envelopes are indicated by red rectangles (two in E1 and one in E2).
(TIF)
