# Supplementary material for: A protein coevolution method uncovers critical features of the Hepatitis C Virus fusion mechanism
Source: PLoS Pathog. 2018 Mar 5;14(3):e1006908. doi: 10.1371/journal.ppat.1006908 (PMC5854445; doi:10.1371/journal.ppat.1006908)
Supplement: S1 Text — (DOCX) [file ppat.1006908.s001.docx]

**Detailed analysis of genotype 1a HCV E1E2 clusters**

Multifunctional cluster 4 was strongly supportive of the E2core structure (**S5A Fig**) as several blocks located within central β-sandwich region were in close proximity. Some blocks in cluster 4 were present within E1, suggesting close structural and functional interactions between E1 and E2 through these blocks. As both E1 and E2 transmembrane domains harbored blocks in cluster 4, these blocks may be involved in the heterodimerization process. Although structural cluster 11 (orange; **S5B Fig**) could also support some regions of the E2core central scaffold, structural clusters 2 and 6 suggested that particular regions of the E2core N-terminal domain (green and red blocks within E2 front layer; **S5B Fig**) could be at close proximity of the E2 C-terminal regions and of E1 central domains. Interestingly, fusion clusters (**Fig.3C,D**) poorly supported E2core as being involved *per se* in membrane fusion. Indeed, fusion clusters either displayed distant E2 blocks, or single E2 blocks coevolving with several E1 blocks, which rather suggested that fusion may involve important E1 and E2 interactions and, possibly, rearrangements. Moreover, the fusion clusters involving E1 and E2 blocks may also suggest that E1E2 rearrangements may be interdependent during fusion (**Fig.3C,D**).

Interestingly, although structural and multifunctional clusters mostly involved blocks located within the front layer and central beta-sandwich of the E2 protein (**S5A,B Fig**), most of the fusion-specific clusters appeared concentrated within the BL (clusters 5,7,8,10,12 in **Fig.3C,D**). By being classified as a multifunctional cluster, cluster 4 not only suggested E2core pre-fusion structural interactions (as structural cluster) but also that several E1-E2 dialogs mediate, in an interdependent manner, fusogenic structural rearrangements (**S5A Fig**). E1 division into three large sub-domains (N-term, central and C-term) by cluster 4 (see the distribution of cluster 4 blocks in E1 in **S5A Fig**) is interpreted as representing the scaffold of a rigid structure of E1 or, alternatively, as a pattern of E1 rearrangement. Indeed, cluster 4, 7 and 10 also suggested that the N- and C-terminal domains of E1 may rearrange to become at close proximity during fusion (**Fig.3C,D; S5A Fig)**. Altogether, this may imply that the general structure of E1 could undergo a fold-over during membrane fusion to mediate the formation of a hairpin structure similar to fusion proteins. Moreover, clusters 7 and 10 also suggest that this putative fold-over could be tightly linked to the packing of E2 C-terminal regions (Stem and Tmd) with the E2 BL. Position of cluster 8 and 12 blocks were consistent with this hypothesis (**Fig.3C,D**). Consistently, similar dialogs were found by BIS during analysis of gt1 (1a+1b) sequences. Intra-E2 fusion cluster 12 organization is also consistent with previous findings showing CD81 binding is a critical fusion-dependent priming step [35] as this fusion cluster harbors a block located within the CD81 binding loop (purple; **Fig.3C,D**). Altogether, fusion clusters suggested that fusion rearrangements may involve a strong proximity of the E1 N- and C-terminal regions, as well as a packing of E2 domains that could gather the E2 C-terminal region close to the E2 central scaffold. Consequently, the E2 C-terminal domain could get spatially close to the E1 N- and C-terminal regions, through rearrangements that are also supported by “undefined role” clusters 9, 13 and 15 (**S5C Fig**). Importantly, the E2 BL appeared as a recurrent component among all the fusion clusters, thus suggesting a role in fusogenic rearrangements.
