# Supplementary material for: A protein coevolution method uncovers critical features of the Hepatitis C Virus fusion mechanism
Source: PLoS Pathog. 2018 Mar 5;14(3):e1006908. doi: 10.1371/journal.ppat.1006908 (PMC5854445; doi:10.1371/journal.ppat.1006908)
Supplement: S2 Text — (DOCX) [file ppat.1006908.s002.docx]

**Detailed analysis of genotype 2 HCV E1E2 clusters**

Interestingly, the organization of the structural clusters 19 (**S8A Fig**), of the structural cluster 11 (**S8B,D Fig**) and of the multifunctional cluster 12 (**S8C,D Fig**) supported the E2core structure as shown by the close proximity of their blocks, which are distant on the linear E2 sequence. When E1E2 are incorporated as heterodimers onto viral particles, gt2 structural clusters suggested that particular regions of the E2 variable region 2 (VR2) may be in close proximity of E1 N-terminal domain (cluster 11) or E1 central domain (cluster 7) (**S8B Fig**). Through its similarity with cluster 7, the undefined cluster 9 could also be involved in such structural organization (**S8F Fig**). Interestingly, the structural cluster 8 pointed out a proximity of the BL with the front layer and with the C-terminal end of E1 (**S8B Fig**), suggesting that in the context of E1E2 heterodimers incorporated onto viral particles, particular E2 C-terminal regions could be in close proximity with the E2 front layer. This hypothesis appears to be consistent with gt1a structural cluster 6 organization (**S5B Fig**). The undefined cluster 20 (**S8F Fig**) also displayed a similarity with the structural cluster 21 (**S8B Fig**) as they both involved a coevolution between a block within the C-terminal end of the BL and a block within the Stem region, thus suggesting an interdependent folding of the Stem and the BL C-terminal regions.

As for gt1a clusters, fusion-specific co-evolving blocks of gt2 did not support E2core structure (**Fig.3E; S8E Fig**). Such organization was consistent with the idea that HCV fusion requires drastic E1E2 rearrangements and re-folding of several protein(s) domains. As proposed for gt1a fusion clusters, the gt2 E2 BL seemed to play an important role in these rearrangements as this domain was found in many clusters (clusters 5,10,13; **Fig.3E, S8C Fig**). Fusion cluster 10 (dark green; **Fig.3E**) suggested that both the β-sandwich and the E2 BL) could rearrange during fusion and get at close proximity, thus allowing a strong packing of the E2 sub-domains. Such assumption was reinforced by the organization of the intra-E2 fusion cluster 16 (red domains and red double arrow, **Fig.3E**) and of the undefined cluster 4 (blue; **S8F Fig**) that suggested similar E2 rearrangements.

Similarly, consistently with gt1a fusion clusters (clusters 4,7,10; **Fig.3C,D; S5A Fig**), the cluster 10, and to a lesser extent the cluster 6, also involved distant E1 blocks within the E1 linear sequence. This suggests that strong intra-E1 rearrangements and a potential E1 fold-over could mediate during fusion the formation of a hairpin structures similar to other fusion proteins (**Fig.3E; S8E Fig**). Finally, cluster 10 (dark green; **Fig.3E**) harbored a block located within the CD81 binding loop similarly to gt1a fusion cluster 12 (purple; **Fig.3D**), which is consistent with the fact that the CD81 binding step is tightly linked to virus fusion [35]. Multifunctional cluster 5 involved a coevolution between a N-terminal region of the BL with a central domain of E1 (**S8C Fig**). Interestingly, the potential fusogenic involvement of this cluster presented important similarities with gt1a fusion-specific cluster 5 (**Fig.3C**).

Altogether, the organization of gt2 fusion clusters allowed to predict fusogenic rearrangements that were consistent with that predicted by gt1a fusion clusters. Although E2 BL could be in close proximity of the E2 front layer in a pre-fusion conformation, E1E2 post-fusion conformation could exhibit a close proximity between the E1 N- and C-terminal regions, the E2 central scaffold and the E2 C terminal regions. Undefined cluster 14 and fusion cluster 17 were supportive of this possible hypothesis (**S8E,F Fig**). Altogether, consistently with the E1E2 fusogenic rearrangement predictions made with sequences of gt1a, the gt2 E2 BL also appears to be an important mediator of E1E2 fusogenic rearrangements.
