# Supplementary material for: A protein coevolution method uncovers critical features of the Hepatitis C Virus fusion mechanism
Source: PLoS Pathog. 2018 Mar 5;14(3):e1006908. doi: 10.1371/journal.ppat.1006908 (PMC5854445; doi:10.1371/journal.ppat.1006908)
Supplement: S1 Table — 17 amino acids sequences of DENV E and PrM serotype 2 were aligned and 14 Clusters were identified by BIS. Clusters are computed with the BIS coevolution analysis method [22–24] and they correspond to maximum scores (symmetricity and environmental scores are set to 1, and the number of admissible exceptions to 0 or 1). For each cluster, the positions of the different coevolving residues or blocks (the initial and final position of each block is reported), and corresponding p-value, are indicated. BIS considered the first amino-acid of PrM as position 1 for all the analyzed sequences. For each cluster, the frequency of its most conserved residues is given (“conservation score”). It should be noted that in BIS, when scores are maximal (that is, set to 1 as for this analysis), all blocks/residues in a cluster display the same amino-acid distribution (See the identical distribution of residues for the two coevolving positions in the alignment of S1 Fig as an example). (DOCX) [file ppat.1006908.s003.docx]

| **Cluster ID** | **Blocks** | **p-value** |
| --- | --- | --- |
| cluster1 | 1-14 17-25 27 30 33-38 41-48 50-51 53-54 56 58-81 83-119 121-126 128-133 136-147 149-151 153-160 163-165 167-194 196-201 203-208 211-222 224-226 228-235 238-240 242-283 285-292 295-301 303-311 313-321 323-331 333-352 354-364 366 368-369 371 373-379 381 383-389 391-395 397-400 402 404 406-410 412-443 445-466 468-509 511-517 519-548 550-562 564-571 573-580 582-599 602-605 607-618 620 622-630 632-643 645-702 704-718 720-724 726-731 734 736 | 1 |
| cluster2 | 50-54 58-119 136-151 211-226 383-395 | 8.080155e−05 |
| cluster3 | 83-126 167-201 | 1.346693e−05 |
| cluster4 | 121-133 196-208 | 0.05882353 |
| cluster5 | 128-134 203-209 371-379 397-402 | 0.05882353 |
| cluster6 | 135-147 210-222 468-517 607-620 733-734 | 0.05882353 |
| cluster7 | 1-15 33-39 323-352 726-732 | 8.080155e−05 |
| cluster8 | 149-160 224-235 | 4.113534e−05 |
| cluster9 | 30-31 53-56 153-161 228-236 313-331 373-381 720-731 | 0.007352941 |
| cluster10 | 162-194 237-283 294-311 | 0.05882353 |
| cluster11 | 16-25 381-389 445-509 | 0.05882353 |
| cluster12 | 17-27 32-38 40-48 242-292 333-369 511-548 601-605 632-702 | 0.05882353 |
| cluster13 | 391-400 582-600 | 0.05882353 |
| cluster14 | 564-580 620-630 | 0.05882353 |

**S1 Table.** **Clusters of coevolving residues identified by BIS in DENV envelope glycoprotein E and PrM sequences of serotype 2.** 17 amino acids sequences of DENV E and PrM serotype 2 were aligned and 14 Clusters were identified by BIS**.** Clusters are computed with the BIS coevolution analysis method [22-24] and they correspond to maximum scores (symmetricity and environmental scores are set to 1, and the number of admissible exceptions to 0 or 1). For each cluster, the positions of the different coevolving residues or blocks (the initial and final position of each block is reported), and corresponding p-value, are indicated. BIS considered the first amino-acid of PrM as position 1 for all the analyzed sequences. For each cluster, the frequency of its most conserved residues is given (“conservation score”). It should be noted that in BIS, when scores are maximal (that is, set to 1 as for this analysis), all blocks/residues in a cluster display the same amino-acid distribution (See the identical distribution of residues for the two coevolving positions in the alignment of **S1** **Fig** as an example).
