# Supplementary material for: A protein coevolution method uncovers critical features of the Hepatitis C Virus fusion mechanism
Source: PLoS Pathog. 2018 Mar 5;14(3):e1006908. doi: 10.1371/journal.ppat.1006908 (PMC5854445; doi:10.1371/journal.ppat.1006908)
Supplement: S2 Table — Clusters are computed with the BIS coevolution analysis method [22–24] and they correspond to maximum scores (symmetricity and environmental scores are set to 1, and the number of admissible exceptions to 0 or 1). For each cluster, the positions of the different coevolving residues or blocks (the initial and final position of each block is reported) and the corresponding p-value are indicated. BIS considered the first amino-acid of E as position 1 for all the analyzed sequences. For each cluster, the frequency of its most conserved residues is given (“conservation score”). It should be noted that in BIS, when scores are maximal (that is, set to 1 as for this analysis), all blocks/residues in a cluster display the same amino-acid distribution (See the identical distribution of residues for the two coevolving positions in the alignment of S1 Fig as an example). (DOCX) [file ppat.1006908.s004.docx]

| **Cluster ID** | **Blocks** | **p-value** |
| --- | --- | --- |
| cluster1 | 28 36-42 54-58 60-62 64 66-67 69-70 72-79 82 84-87 92-94 97-111 114-117 119 121 126-128 130 133-135 137-138 142 144 146 151-152 165-167 172-173 175 178-180 184-196 198-199 201 204-206 208-213 215-220 223-224 231 234-235 237-242 244-248 250-271 273 275 279 281-287 289-292 294-299 301-302 306 310-311 314-316 318-320 324 326 328 332-336 30-34 341 349-353 355-356 361 364-374 376 380-381 387-388 391 399 401-403 405 407-409 411-424 426-428 431 433-434 437-438 440-441 444 446 44-45 448-453 455-457 459-461 464-467 469-470 473-474 476-476 478 483 47-49 486-490 493-495 | 1 |
| cluster2 | 28-34 36-49 51 53-62 64-67 72-80 84-90 92-123 125-128 133-140 142-148 150-152 160-161 163-163 165-168 170 175-202 204-220 222-225 227 229-232 234-271 273-302 304 306-307 309-316 318-321 323-336 338-339 341-345 347-357 360-377 380-384 386-389 391-453 455-461 463-480 482-483 | 0.00042 |
| cluster3 | 2-3 5 8-14 16-17 20-22 24-26 | 0.0073 |
| cluster4 | 160-163 172-202 | 0.00014 |
| cluster5 | 69-80 455-480 | 2.44e−07 |
| cluster6 | 156 157 | 0.00016 |
| cluster7 | 4 6 7 15 18 19 23 | 5.38e−06 |
| cluster8 | 64-70 92-128 132-140 229-302 304-307 | 0.00010 |
| cluster9 | 27-28 130-131 | 0.058 |
| cluster10 | 30-42 151-153 155 159 | 0.058 |
| cluster11 | 204-225 380-389 391-461 463-483 | 0.00010 |
| cluster12 | 227-232 341-357 | 3.23e−05 |

**S2 Table.** **Clusters of coevolving residues identified by BIS in DENV envelope glycoprotein E sequences of serotype 2.** Clusters are computed with the BIS coevolution analysis method [22-24] and they correspond to maximum scores (symmetricity and environmental scores are set to 1, and the number of admissible exceptions to 0 or 1). For each cluster, the positions of the different coevolving residues or blocks (the initial and final position of each block is reported) and the corresponding p-value are indicated. BIS considered the first amino-acid of E as position 1 for all the analyzed sequences. For each cluster, the frequency of its most conserved residues is given (“conservation score”). It should be noted that in BIS, when scores are maximal (that is, set to 1 as for this analysis), all blocks/residues in a cluster display the same amino-acid distribution (See the identical distribution of residues for the two coevolving positions in the alignment of **S1 Fig** as an example).
