# Supplementary material for: A protein coevolution method uncovers critical features of the Hepatitis C Virus fusion mechanism
Source: PLoS Pathog. 2018 Mar 5;14(3):e1006908. doi: 10.1371/journal.ppat.1006908 (PMC5854445; doi:10.1371/journal.ppat.1006908)
Supplement: S3 Table — Ten groups of sequences were assembled and analyzed independently with the BIS method. Groups were constituted of E1E2 sequences from HCV types and sub-types from genotype 1a to 6a. Groups of sequences from genotypes 1 and 2 were constituted by pools of sequences from subtypes 1a and 1b (50 sequences) and sequences of genotypes 2a and 2b (30 sequences). Total numbers of detected clusters for each genotype and sub-type is reported, as well as the number of statistically significant clusters among them (when p<0.05). For each group of sequence, we also report the number of statistically significant clusters only involving E1 positions (“intra-E1”), the number of clusters only involving E2 positions (“intra-E2”), and the number of clusters across E1 and E2 (“inter-E1-E2”). The assignment of a given cluster block to E1 or E2 was determined by mapping the reference genome sequence of genotype 1b (accession: AJ238799) to the multiple sequence alignment, for each genotype. E1 and E2 were identified on AJ238799 at positions 192–383 and 384–746, respectively. Note however that residue positions displayed in S4 Table, S7 Table and in the related HCV webserver (http://www.lcqb.upmc.fr/HCVenv/HCVenv.html) are specific to each genotype and set of patient sequences analyzed. (DOCX) [file ppat.1006908.s005.docx]

|  | | **Genotypes and sub-types** | | | | | | | | | |
| --- | --- | --- | --- | --- | --- | --- | --- | --- | --- | --- | --- |
|  |  | **1a** | **1b** | **1** | **2a** | **2b** | **2** | **3** | **4a** | **5a** | **6a** |
| **Number of sequences processed** | | 25 | 25 | 50 | 15 | 15 | 30 | 15 | 15 | 10 | 15 |
| **Number of clusters detected** | Total detected | 16 | 21 | 20 | 20 | 15 | 21 | 18 | 14 | 11 | 13 |
|  | Total (p<0.05) | **15** | **20** | **19** | **9** | **3** | **20** | **17** | **2** | **2** | **1** |
| **Clusters location** | intra-E1 (p<0.05) | 1 | 2 | 3 | 0 | 1 | 1 | 2 | 0 | 0 | 1 |
|  | intra-E2 (p<0.05) | 4 | 3 | 4 | 2 | 1 | 7 | 6 | 1 | 1 | 0 |
|  | inter-E1-E2 (p<0.05) | **10** | **15** | **12** | **7** | **1** | **12** | **9** | **1** | **1** | **0** |

**S3 Table.** **BIS coevolution analysis of HCV E1E2 sequences**. Ten groups of sequences were assembled and analyzed independently with the BIS method. Groups were constituted of E1E2 sequences from HCV types and sub-types from genotype 1a to 6a. Groups of sequences from genotypes 1 and 2 were constituted by pools of sequences from subtypes 1a and 1b (50 sequences) and sequences of genotypes 2a and 2b (30 sequences). Total numbers of detected clusters for each genotype and sub-type is reported, as well as the number of statistically significant clusters among them (when p<0.05). For each group of sequence, we also report the number of statistically significant clusters only involving E1 positions (“intra-E1”), the number of clusters only involving E2 positions (“intra-E2”), and the number of clusters across E1 and E2 (“inter-E1-E2”). The assignment of a given cluster block to E1 or E2 was determined by mapping the reference genome sequence of genotype 1b (accession: AJ238799) to the multiple sequence alignment, for each genotype. E1 and E2 were identified on AJ238799 at positions 192-383 and 384-746, respectively. Note however that residue positions displayed in **S4 Table**, **S7 Table** and in the related HCV webserver (http://www.lcqb.upmc.fr/HCVenv/HCVenv.html) are specific to each genotype and set of patient sequences analyzed.
