# Supplementary material for: A protein coevolution method uncovers critical features of the Hepatitis C Virus fusion mechanism
Source: PLoS Pathog. 2018 Mar 5;14(3):e1006908. doi: 10.1371/journal.ppat.1006908 (PMC5854445; doi:10.1371/journal.ppat.1006908)
Supplement: S4 Table — Clusters are computed with the BIS analysis method similarly to S1 Table. Note that residue positions displayed in this table are specific to the set of patient sequences analyzed. Hence, nucleotide gaps generated during the analysis of the patient sequences by BIS were taken into account when plotting gt1a clusters into a gt1a reference E1E2 reference (H77, AF009606; (S4 Fig) and into gt1a E2core structure (S5 Fig). (DOCX) [file ppat.1006908.s006.docx]

| **Cluster ID** | **Blocks** | **p-value** |
| --- | --- | --- |
| cluster1 | 56 63 65 68-69  72-76 8 81-83 85 87-93 95 98-105 107 111 113 115 118-121 123-129 131-137 140-141 145 147-148 150-152 155-156 10-16 158-167 170 172-173 175-181 183 185-188 190-191 194 198-199 18-20 215 218 222 226-230 232 234-239 22-24 245 248 250 252 256-257 259 59-61 263-264 267-268 27 274 276-277 279-281 29 292-298 3-5 300 302-303 306-308 31 311-315 317-323 325-329 332 335 338-339 343-345 33-35 348-354 356-365 368 370-371 373-374 376-378 380 382 384-385 387-388  390-397 399 37-40 401-403 406-410 413-416 418 422-430  432 436-437 439-441 443-444 446-449 45452-455 457-461 463 465-4762 478-479 481-482 485-489 47-49 492-494  406-504 507-513 515-516 520 524 527 530 532-533 535-539 53-54 541 543-546 549-550 552 554-555 | 1 |
| cluster2 | 59 245-246 | 0.0033333 |
| cluster3 | 241 242 | 0.0033333 |
| cluster4 | 3-6 10-20 29-31 56-57 60 62-63 67-69 71-76 79-85 87-95 107-111 144-145 150-153 155-167 170-173  183-188 190-192 271 276-281 302-304 348-365 370-378 384-388 405-410 478-482 507-517 520-521 532-539 549-552 | 0.04 |
| cluster5 | 78 113-115 452-461 | 0.04 |
| cluster6 | 115-116 244-245 522 | 0.04 |
| cluster7 | 18-24 123-137 169-170 390-399 406-411 | 0.04 |
| cluster8 | 131-138 380-382 439-444 485-494 552-555 | 0.04 |
| cluster9 | 8-16 436-441 | 0.04 |
| cluster10 | 37-41 185-191 420 496-513 524-525 527-528 | 0.04 |
| cluster11 | 221-222 256-261 267-269 311-323 | 0.04 |
| cluster12 | 226-232 341 434 | 0.04 |
| cluster13 | 33-40 465-479 | 0.04 |
| cluster14 | 399-403 484-489 | 0.04 |
| cluster15 | 43 530-533 548-550 | 0.04 |
| cluster16 | 65-69 71-85 | 0.0033333 |

**S4 Table.** **Clusters of coevolving residues identified by BIS in HCV E1E2 sequences of genotype 1a.** Clusters are computed with the BIS analysis method similarly to **S1 Table**. Note that residue positions displayed in this table are specific to the set of patient sequences analyzed. Hence, nucleotide gaps generated during the analysis of the patient sequences by BIS were taken into account when plotting gt1a clusters into a gt1a reference E1E2 reference (H77, AF009606; **S4 Fig**) and into gt1a E2core structure (**S5 Fig**).
