# Supplementary material for: A protein coevolution method uncovers critical features of the Hepatitis C Virus fusion mechanism
Source: PLoS Pathog. 2018 Mar 5;14(3):e1006908. doi: 10.1371/journal.ppat.1006908 (PMC5854445; doi:10.1371/journal.ppat.1006908)
Supplement: S5 Table — For each block, the initial and final position of the block predicted by BIS and the name of the cluster it belongs to are given. Blocks from each cluster are numerated from 1 to x (Block N°) to easily identify their position on the E1E2 sequences in S4 Fig, where each block is referenced as follow: “Cluster ID-Block N°”. (DOCX) [file ppat.1006908.s007.docx]

| **E1 - Gt 1a** | | | **E2 - Gt 1a** | | |
| --- | --- | --- | --- | --- | --- |
| Clusters | Block N° | Block Position | Clusters | Block N° | Block Position |
| 4 | 1 | 29-31 | 11 | 1 | 221-222 |
| 4 | 2 | 67-69 | 12 | 1 | 226-232 |
| 16 | 1 | 65-69 | 6 | 1 | 244-245 |
| 4 | 3 | 71-76 | 2 | 1 | 245-246 |
| 16 | 2 | 71-85 | 11 | 2 | 256-261 |
| 5 | 1 | 78 | 11 | 3 | 311-323 |
| 4 | 4 | 79-85 | 12 | 2 | 341 |
| 4 | 5 | 87-95 | 7 | 1 | 390-399 |
| 4 | 6 | 107-111 | 7 | 2 | 406-411 |
| 6 | 1 | 115-116 | 10 | 1 | 420 |
| 8 | 1 | 131-138 | 8 | 1 | 485-494 |
| 4 | 7 | 144-145 | 10 | 2 | 496-513 |
| 4 | 8 | 150-153 | 4 | 1 | 532-539 |
| 4 | 9 | 155-167 |  |  |  |
| 7 | 1 | 169-170 |  |  |  |
| 4 | 10 | 170-173 |  |  |  |
| 4 | 11 | 183-188 |  |  |  |

**S5 Table. List of Genotype 1a cluster blocks mapped on E1E2 references sequences (H77, AF009606).** For each block, the initial and final position of the block predicted by BIS and the name of the cluster it belongs to are given. Blocks from each cluster are numerated from 1 to x (Block N°) to easily identify their position on the E1E2 sequences in **S4 Fig**, where each block is referenced as follow: “Cluster ID-Block N°”.
