# Supplementary material for: A protein coevolution method uncovers critical features of the Hepatitis C Virus fusion mechanism
Source: PLoS Pathog. 2018 Mar 5;14(3):e1006908. doi: 10.1371/journal.ppat.1006908 (PMC5854445; doi:10.1371/journal.ppat.1006908)
Supplement: S6 Table — We aligned 25 E1E2 amino acids sequences of HCV genotype 1a and identified using BIS method 16 clusters (S3 Fig; S4 Table). Genotype 1a clusters harboring blocks that mapped residues previously reported in the literature to have a specific function (S4 Fig) are classified. The known role(s) are indicated: Folding or heterodimerization (blue), viral binding site conformation (green) or fusion mechanism (red). According to these roles, clusters are categorized into three different categories: structural (Folding, heterodimerization and viral binding site conformation), fusion or multifunctional cluster. Clusters harboring blocks that did not map any residues with a previously reported function were classified as clusters with “undefined role. (DOCX) [file ppat.1006908.s008.docx]

| **Genotype 1a clusters** | |  |  |  |
| --- | --- | --- | --- | --- |
| Structural clusters | 2 |  | X |  |
|  | 6 | X | X |  |
|  | 11 |  | X |  |
| Fusion clusters | 5 |  |  | X |
|  | 7 | X |  | X |
|  | 8 |  |  | X |
|  | 10 | X |  | X |
|  | 12 |  | X | X |
| Multifunctional Clusters | 4 | X | X | X |
|  | 16 | X | X | X |
| Undefined role | 3 | ? | | |
|  | 9 | ? | | |
|  | 13 | ? | | |
|  | 14 | ? | | |
|  | 15 | ? | | |

**S6 Table.** **Putative functions of genotype 1a E1E2 coevolution clusters.** We aligned 25 E1E2 amino acids sequences of HCV genotype 1a and identified using BIS method 16 clusters (**S3 Fig; S4 Table**). Genotype 1a clusters harboring blocks that mapped residues previously reported in the literature to have a specific function (**S4 Fig**) are classified. The known role(s) are indicated: Folding or heterodimerization (blue), viral binding site conformation (green) or fusion mechanism (red). According to these roles, clusters are categorized into three different categories: structural (Folding, heterodimerization and viral binding site conformation), fusion or multifunctional cluster. Clusters harboring blocks that did not map any residues with a previously reported function were classified as clusters with “undefined role.
