# Supplementary material for: A protein coevolution method uncovers critical features of the Hepatitis C Virus fusion mechanism
Source: PLoS Pathog. 2018 Mar 5;14(3):e1006908. doi: 10.1371/journal.ppat.1006908 (PMC5854445; doi:10.1371/journal.ppat.1006908)
Supplement: S7 Table — Clusters are computed with the BIS analysis method similarly to S1 Table. Note that residue positions displayed in this table are specific to the set of patient sequences analyzed. Hence, nucleotide gaps generated during the analysis of the patient sequences by BIS, as well as gaps between gt1a and gt2 sequences, were taken into account when plotting gt2 clusters into a gt2 reference E1E2 reference (JFH-1, AB047639; S7 Fig) and into gt1a E2core structure (S8 Fig). (DOCX) [file ppat.1006908.s009.docx]

| **Cluster ID** | **Blocks** | **p-value** |
| --- | --- | --- |
| cluster1 | 5 14-16 20-24 28-31 33-39 48-49 52-52 54-55 57-58 62-65 68-69 7 71-71 73 76 79-80 82-89 91-92 95 10 105-105 110 112 114-117 119 121 125 128-133 136-139 141-142 144-146 149-149 151 160 162-165 167-169 171-181 184-186 190-193 218 225 229-235 237-241 244-245 247-249 254-255 260 262-264 267 271-272 277-278 280-282 284 286 290-292 296-301 303-305 308-309 311 316 318-319 321-322 324-325 327 329-332 334 339-341 343-344 346 348-351 354-359 361 364-369 373 375-384 386 388 390 394-406 410 413-414 416-419 421 423 427-431 435-436 439-439 444 446-448 450-450 453-455 458-460 464-465 468-468 470-470 473-475 477 480-488 492-493 496 498-505 507-514 516 519-521 523-524 526 530 532-535 538-546 548-550 552-555 557 559-562 | 1 |
| cluster2 | 10-11 28-39 46-46 51-55 75-76 71-73 78-80 91-93 110-112 128-134 148-149 160-165 274 490 518-521 552-557 | 1.289345e−08 |
| cluster3 | 12 98 | 6.876507e−09 |
| cluster4 | 307-309 439-440 | 8.350044e-09 |
| cluster5 | 99 104-105 427-436 | 0.033333 |
| cluster6 | 107-108 141-146 480-489 | 0.033333 |
| cluster7 | 109-112 274-275 284-286 | 4.297817e−10 |
| cluster8 | 127-133 259-260 450-451 | 0.033333 |
| cluster9 | 135-139 294 | 0.033333 |
| cluster10 | 18 151-152 221 346-351 443-444 | 0.033333 |
| cluster11 | 13-16 296-305 409-410 | 0.033333 |
| cluster12 | 167-181 229-241 316-319 337 | 0.033333 |
| cluster13 | 184-187 452-455 | 0.033333 |
| cluster14 | 189-193 286-287 | 0.033333 |
| cluster15 | 201 348-352 | 0.033333 |
| cluster16 | 237-242 470-475 | 0.033333 |
| cluster17 | 20-25 496-505 | 0.033333 |
| cluster18 | 253-255 324-327 464-466 | 0.033333 |
| cluster19 | 313 318-325 329-334 353-359 363-370 435-437 458-461 557-562 | 0.033333 |
| cluster20 | 462 491-493 | 0.033333 |
| cluster21 | 463-465 507-516 | 0.033333 |

**S7 Table.** **Clusters of coevolving residues identified by BIS in HCV E1E2 sequences of genotype 2.** Clusters are computed with the BIS analysis method similarly to **S1 Table**. Note that residue positions displayed in this table are specific to the set of patient sequences analyzed. Hence, nucleotide gaps generated during the analysis of the patient sequences by BIS, as well as gaps between gt1a and gt2 sequences, were taken into account when plotting gt2 clusters into a gt2 reference E1E2 reference (JFH-1, AB047639; **S7 Fig**) and into gt1a E2core structure (**S8 Fig**).
