# Supplementary material for: A protein coevolution method uncovers critical features of the Hepatitis C Virus fusion mechanism
Source: PLoS Pathog. 2018 Mar 5;14(3):e1006908. doi: 10.1371/journal.ppat.1006908 (PMC5854445; doi:10.1371/journal.ppat.1006908)
Supplement: S9 Table — We aligned 30 E1E2 amino acid sequences of HCV genotype 2 (2a and 2b) and, using the BIS method, we identified 21 clusters (S6 Fig; S3, S7 Table). Genotype 2 clusters harboring blocks that mapped residues previously reported in the literature to have a specific function are classified. The known role(s) of the residues in each cluster are indicated: Folding or heterodimerization (blue), viral binding site conformation (green) or fusion mechanism (red). According to these roles, clusters are categorized into three different categories: structural (Folding, heterodimerization and viral binding site conformation), fusion or multifunctional cluster. Clusters harboring blocks that did not map any residues with a previously reported function were classified as clusters with “undefined role”. (DOCX) [file ppat.1006908.s011.docx]

| **Genotype 2 Clusters** | |  |  |  |
| --- | --- | --- | --- | --- |
| Structural Clusters | 7 | X |  |  |
|  | 8 | X | X |  |
|  | 11 | X |  |  |
|  | 18 | X | X |  |
|  | 19 |  | X |  |
|  | 21 | X |  |  |
| Fusion Clusters | 6 |  |  | X |
|  | 10 |  | X | X |
|  | 13 | X |  | X |
|  | 16 |  |  | X |
|  | 17 |  |  | X |
| Multifunctional Clusters | 2 | X | X | X |
|  | 5 | X | X | X |
|  | 12 | X | X | X |
| Undefined role | 3 | ? | | |
|  | 4 | ? | | |
|  | 9 | ? | | |
|  | 14 | ? | | |
|  | 15 | ? | | |
|  | 20 | ? | | |

**S9 Table.** **Putative functions of genotype 2 E1E2 coevolution clusters.** We aligned 30 E1E2 amino acid sequences of HCV genotype 2 (2a and 2b) and, using the BIS method, we identified 21 clusters (**S6 Fig; S3,7 Table**). Genotype 2 clusters harboring blocks that mapped residues previously reported in the literature to have a specific function are classified. The known role(s) of the residues in each cluster are indicated: Folding or heterodimerization (blue), viral binding site conformation (green) or fusion mechanism (red). According to these roles, clusters are categorized into three different categories: structural (Folding, heterodimerization and viral binding site conformation), fusion or multifunctional cluster. Clusters harboring blocks that did not map any residues with a previously reported function were classified as clusters with “undefined role”.
