# Supplementary material for: A protein coevolution method uncovers critical features of the Hepatitis C Virus fusion mechanism
Source: PLoS Pathog. 2018 Mar 5;14(3):e1006908. doi: 10.1371/journal.ppat.1006908 (PMC5854445; doi:10.1371/journal.ppat.1006908)
Supplement: S10 Table — List of clusters identified by BIS that connect the transmembrane of E1 and E2. Six clusters were identified across the two major HCV genotypes. Location of the transmembrane domains within E1 and E2 are indicated for each genotype and sub-types. Position of the coevolving blocks located within the transmembrane of E1 and E2 are indicated for each cluster. TMD, Transmembrane Domain. Only significant clusters are reported (p value≤0.05). (DOCX) [file ppat.1006908.s012.docx]

| **Sub/Genotype** | **TMD E1** | **TMD E2** | **Cluster ID** | **Block position in E1** | **Block position in E2** |
| --- | --- | --- | --- | --- | --- |
| 1a | 162-192 | 526-555 | 4 | 155-167 170-173 183-188 190-192 | 520-521 532-539 549-552 |
| 1a | 162-192 | 526-555 | 10 | 185-191 | 524-525 527-528 |
| 1b | 162-192 | 529-558 | 11 | 170-173 | 541-542 |
| 1 | 162-192 | 529-558 | 8 | 170-171 190-192 | 554-555 |
| 1 | 162-192 | 529-558 | 10 | 172-173 | 541-542 |
| 2 | 163-193 | 533-562 | 2 | 160-165 | 552-557 |

**S10 Table. BIS analysis of the coevolution of the transmembranes of E1 and E2.** List of clusters identified by BIS that connect the transmembrane of E1 and E2. Six clusters were identified across the two major HCV genotypes. Location of the transmembrane domains within E1 and E2 are indicated for each genotype and sub-types. Position of the coevolving blocks located within the transmembrane of E1 and E2 are indicated for each cluster. TMD, Transmembrane Domain. Only significant clusters are reported (p value≤0.05).
