# Supplementary figures and images for: A protein coevolution method uncovers critical features of the Hepatitis C Virus fusion mechanism
Source: PLoS Pathog. 2018 Mar 5;14(3):e1006908. doi: 10.1371/journal.ppat.1006908 (PMC5854445; doi:10.1371/journal.ppat.1006908)

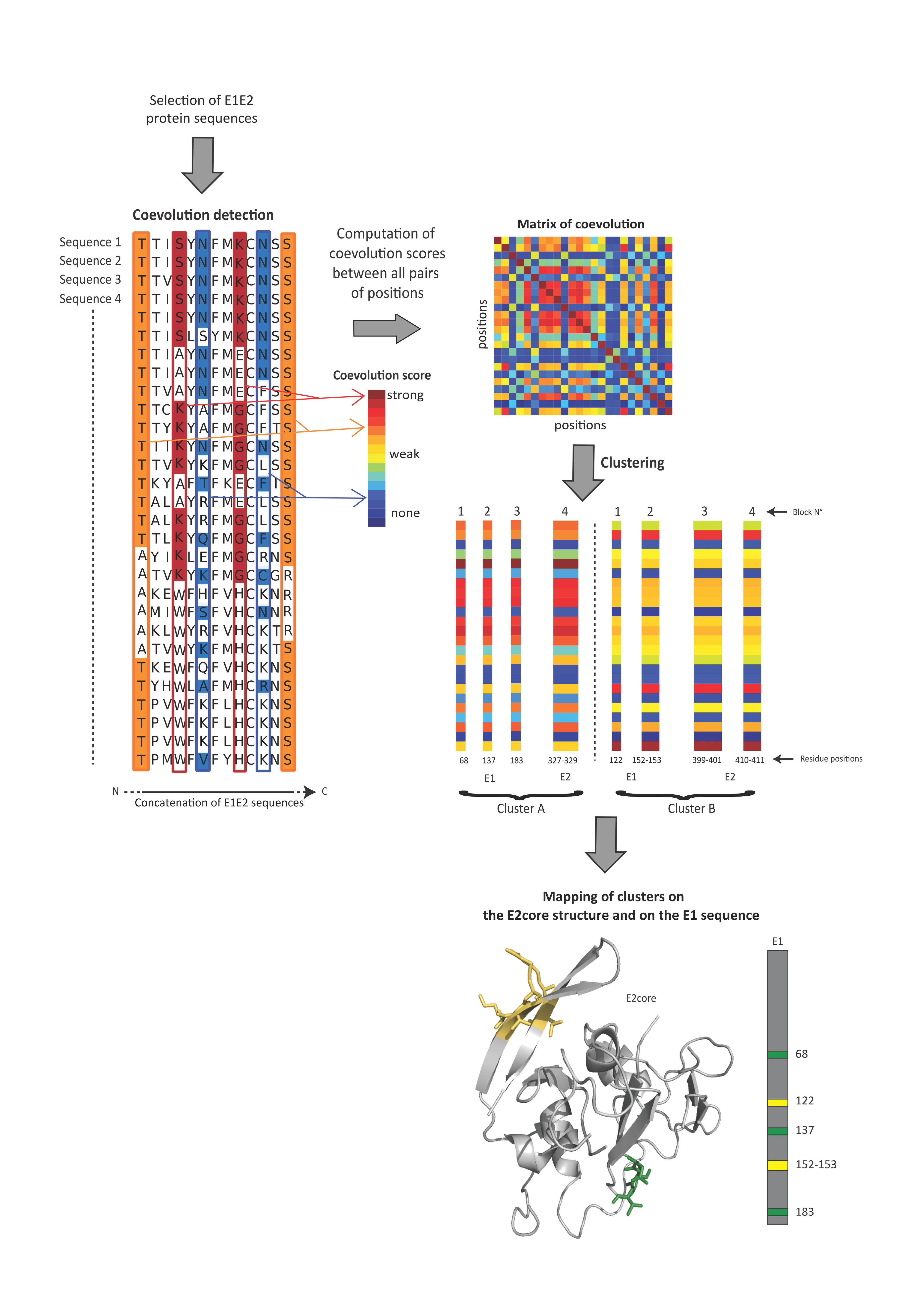

Supplement: S1 Fig — Coevolution analyses require the identification of sequence variability and they are usually realized on families of homologous protein sequences, typically very divergent and represented by a large number of sequences. In contrast, studying viral sequences requires methods that can analyze serotypes and genotypes, usually constituted by a limited number of conserved sequences. BIS allows to track small parallel changes likely corresponding to compensatory patterns preserving the structure and function of the protein. Given a sequence alignment, the coevolution analysis method Blocks In Sequences (BIS) [22–24] identifies groups of residues where mutations are present, at the same time, in the same sequences. BIS allows to track small parallel changes likely corresponding to compensatory patterns preserving the structure and function of the protein. BIS first detects coevolution among each pair of alignment positions (left) and associates a coevolution score to the pairs. At different colors match different coevolution scores (from red for high coevolution score to blue for none). A coevolution score matrix between all alignment positions is constructed (top right) and clustered in such a way that groups of positions displaying the same coevolution scores with all other positions in the alignment are identified (bottom right). The schema illustrates two clusters made of four blocks (cluster A and B). Each cluster is composed of blocks displaying a similar pattern of coevolution scores (here represented by a similar color pattern). Cluster A is constituted by six positions organized in four blocks, three of them corresponding to single residue positions and one of them made of three consecutive residue positions. Cluster B is made of four positions organized in four blocks, one of them corresponding to single residue positions and three of them to two or three consecutive residue positions. For both clusters, blocks belong to either E1 or E2 protein. An illustration o [file ppat.1006908.s013.tif]

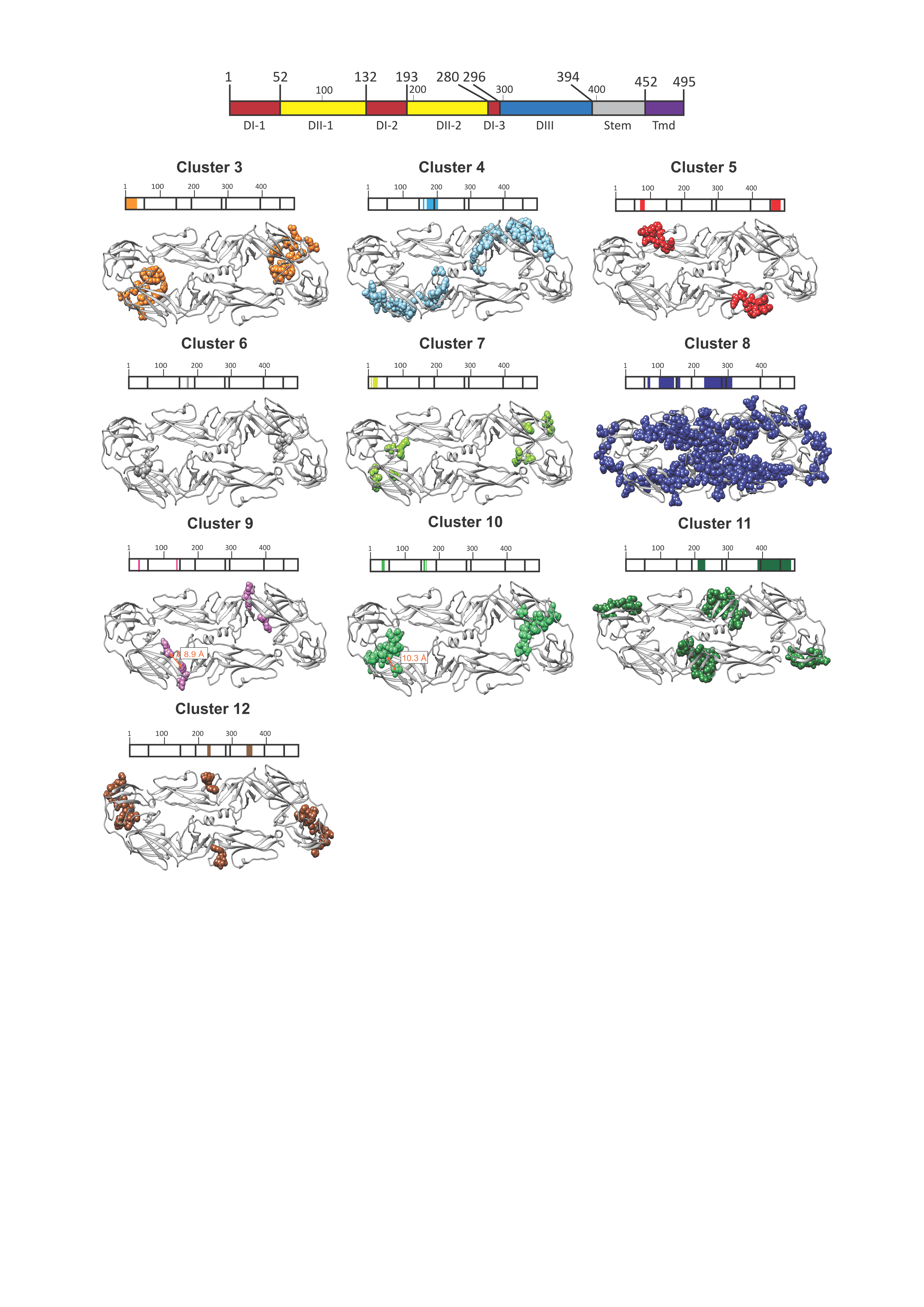

Supplement: S2 Fig — Mapping of the DENV E clusters 3 to 12 (illustrated by distinct colors) on the E dimeric structure (PDB 1K4R). For each cluster, positions of the coevolving blocks in E sequence are displayed within “strips” located above each structure (See S2 Table for cluster positions). Each cluster is identified by a distinct color. Cluster 1 and 2 were too large to be considered. A linear representation of Dengue E protein is also shown at the top of the figure. Starting and ending residue positions of each E domain are indicated. E domains are annotated by distinct colors: DI, domain I (red); DII, domain II (yellow); DIII, domain III (blue); Tmd, transmembrane (black). (TIF) [file ppat.1006908.s014.tif]

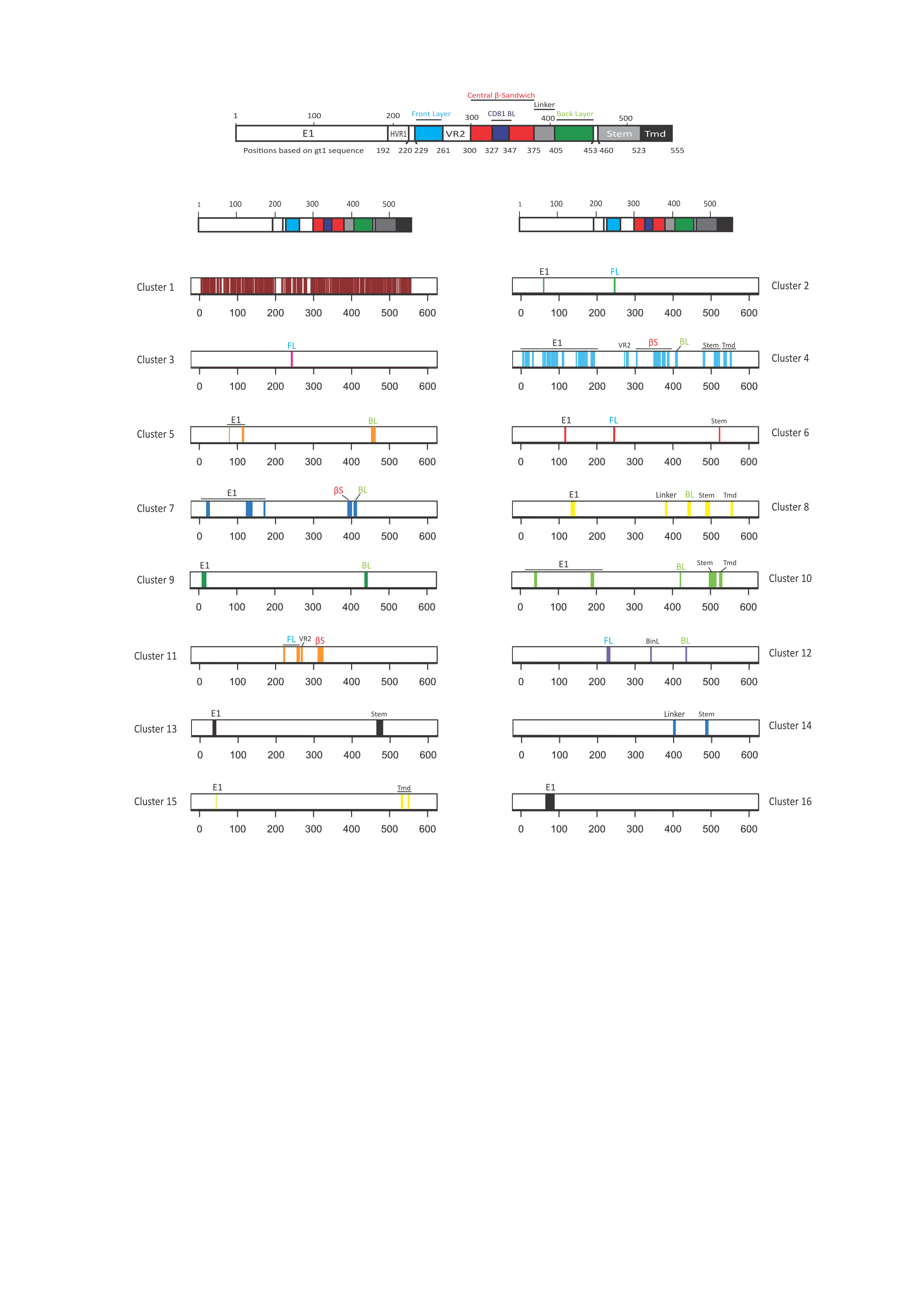

Supplement: S3 Fig — The 16 gt1a clusters (illustrated by distinct colors) are displayed within “strips” representing the E1E2 sequence. For each cluster, positions of the coevolving blocks in the E1E2 sequence are indicated within the corresponding “strip” (see S4 Table for cluster positions). On the top of each coevolving block is indicated the corresponding protein or E2 domain the block belongs to: HVR1, Hyper Variable Region 1; FL, Front Layer; VR2, Variable Region 2; βS, β-Sandwich; BinL, CD81 Binding Loop; BL, Back Layer; Stem; Tmd, Transmembrane domain). Two small linear representations of HCV E1E2 are located at the top of each “strip” column, and can be used as reference for determining the position of each coevolving block within the E1E1 sequence. At the top of the figure is shown an enlarged linear representation of HCV E1E2, where the starting and ending residue positions of each protein and domain are indicated. E2 domains are highlighted by distinct colors (Green, BL; Red, central β-sandwich; Blue, front layer; Dark blue, CD81 BinL/CD81 binding loop; Light grey, central β-sandwich–back layer linker; Black dotted line, VR2/Variable Region 2; Grey dotted line, Stem; Black rectangle, Tmd/Transmembrane). (TIF) [file ppat.1006908.s015.tif]

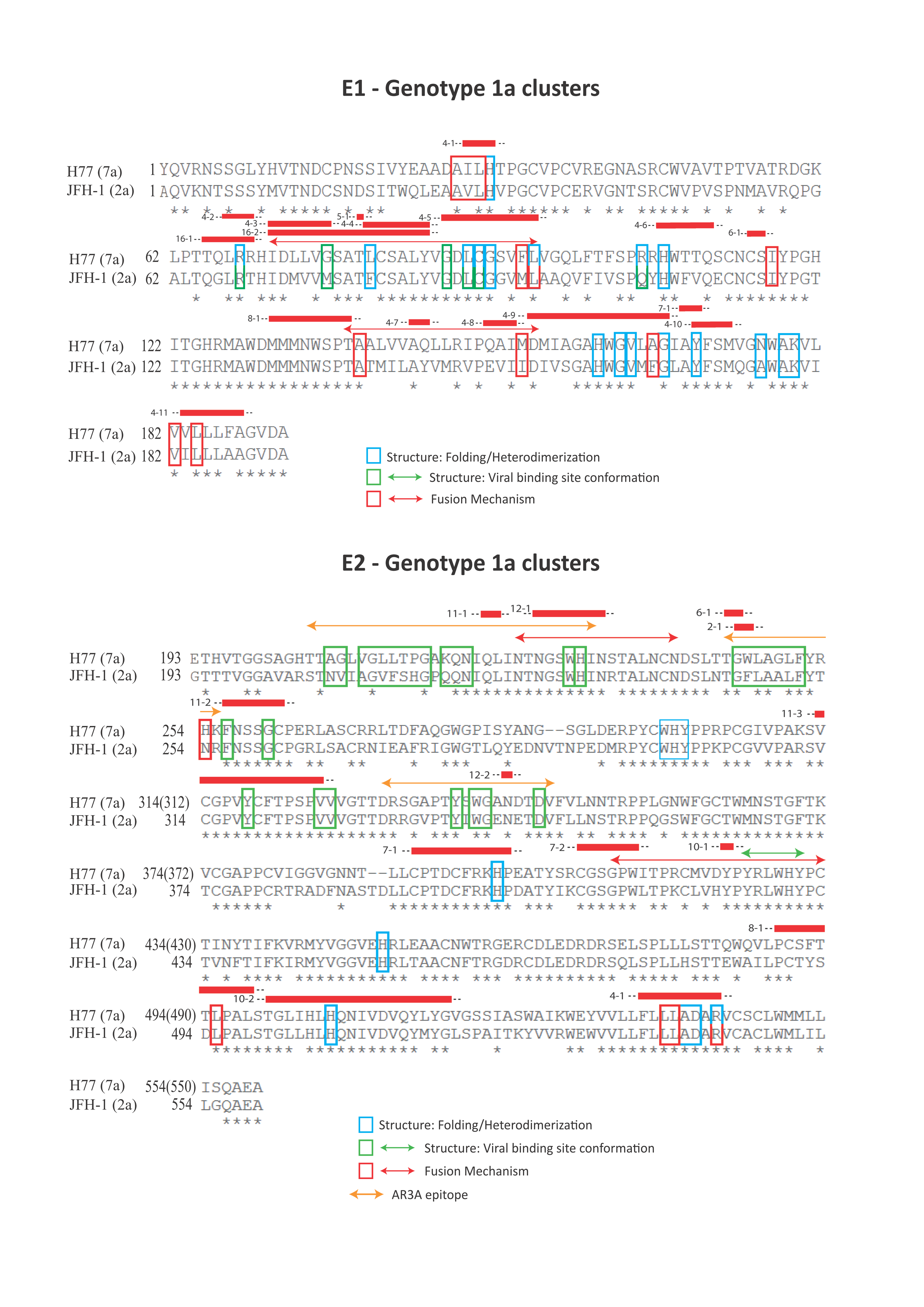

Supplement: S4 Fig — Genotype 1a cluster blocks are represented as red horizontal bars positioned above the E1 and E2 H77 or JFH-1 aligned sequences. Each horizontal bar is numerated as follow: “Cluster ID-Block N°”. Respective positions of these blocks are referenced in S5 Table. Residues (boxes) or domains (horizontal arrows) previously identified in the literature to have a function are indicated (see Materials and Methods for references). A color code, reported below the sequence alignment, links residues (boxes) or domains (arrow) to specific functions according to the literature: blue for folding/heterodimerization, green for binding and red for fusion. Multi-colored code boxes represent residues with two identified functions. In E2, the AR3A antibody epitope is shown as three orange arrows. The putative function determined for each cluster is summarized in S6 Table. The addition of one amino acid on the left and on the right of each block is considered to take into account potential structural adjustments after mutation that are not considered in BIS calculation. Cluster blocks positions are numbered according to H77 gt1a (AF009606; S4 Table). (TIF) [file ppat.1006908.s016.tif]

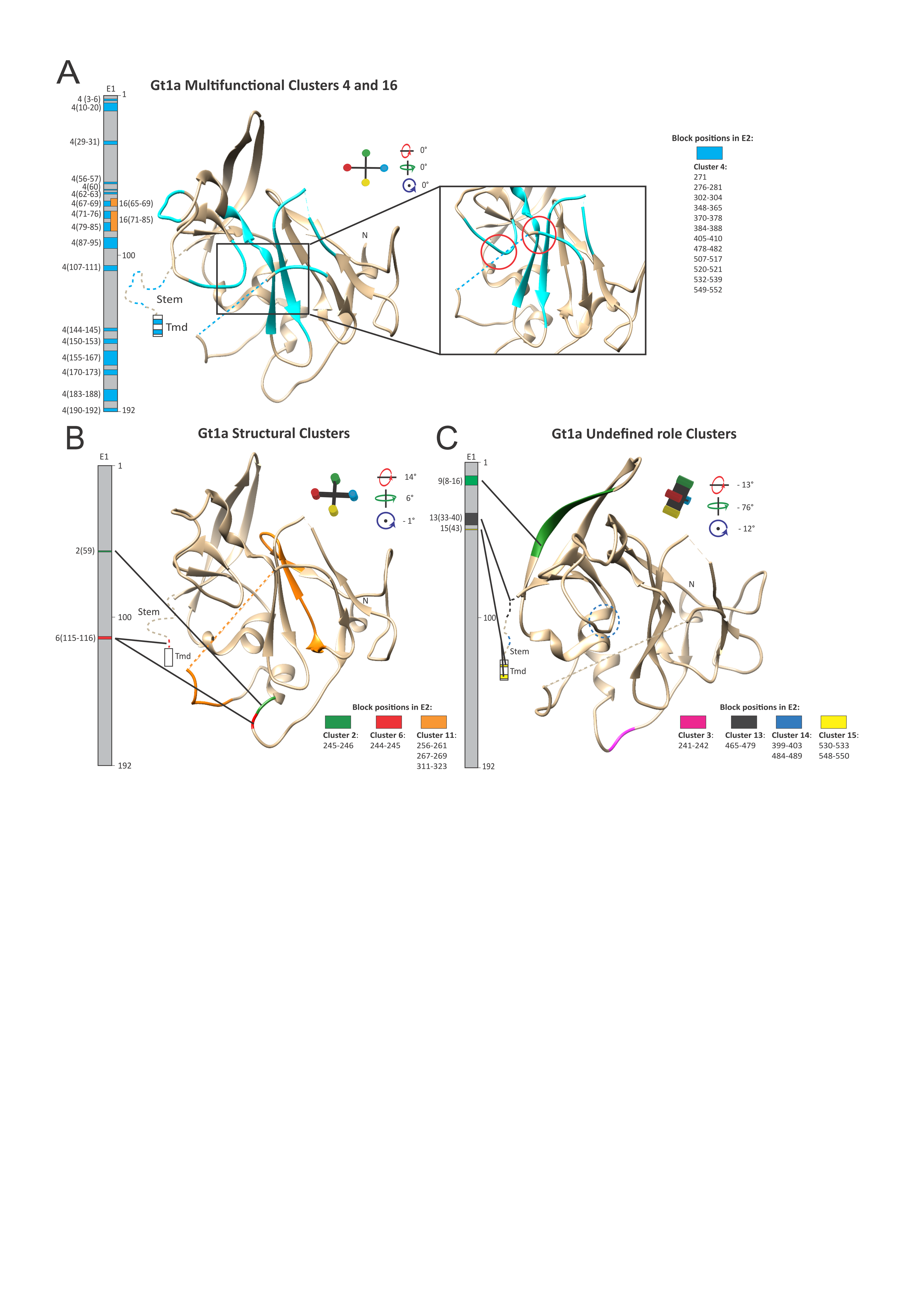

Supplement: S5 Fig — HCV E1E2 gt1a multifunctional clusters 4 (blue) and 16 (orange) (A), structural clusters 11 (orange), 2 (green) and 6 (red) (B) and undefined role clusters 3 (pink), 9 (emerald green), 13 (brown), 14 (blue) and 15 (yellow) (C) are plotted on a vertical linear representation of E1 and on a tridimensional view of E2 core (PDB 4MWF). Each cluster is composed of blocks harboring a similar color, according to S3 Fig. The Stem region (Stem) is represented by a dotted line after the C-terminal part of the BL. The transmembrane domain (Tmd) is represented as a rectangle following the Stem region. In B and C, bold lines link E1 and E2 blocks that coevolve. For each cluster, block positions in E1 (at the left of the linear structure) and E2 (below boxes whose color match the color of the corresponding cluster) are indicated. In A, the E2 central scaffold is enlarged and red circles highlight regions where cluster 4 blocks are in close proximity. Rotation angles of the E2core structure are indicated. Viewing angle of E2core is indicated by a black cross (Reference: Fig 3B). FL, Front Layer; BinL, CD81 Binding Loop; VR2, Variable Region 2; BL, Back Layer; βS, β-Sandwich. (TIF) [file ppat.1006908.s017.tif]

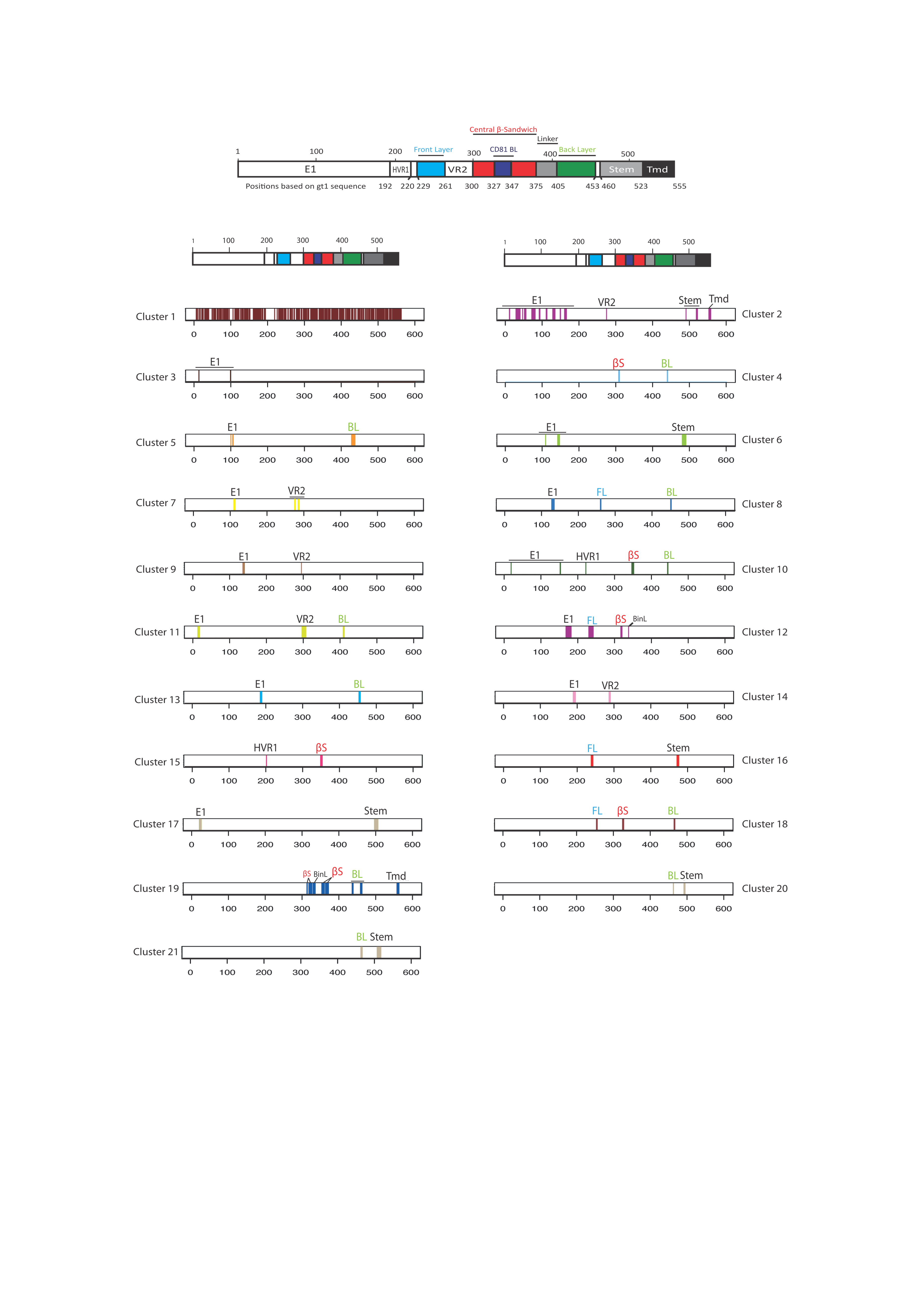

Supplement: S6 Fig — The 21 gt2 clusters (illustrated by distinct colors) are displayed within “strips” representing the E1E2 sequence. For each cluster, positions of the coevolving blocks in the E1E2 sequence are indicated within the corresponding “strip” (see S7 Table for cluster positions). On the top of each coevolving block is indicated the corresponding protein or E2 domain the block belongs to: HVR1, Hyper Variable Region 1; FL, Front Layer; VR2, Variable Region 2; βS, β-Sandwich; BinL, CD81 Binding Loop; BL, Back Layer; Stem; Tmd, Transmembrane domain). Two small linear representations of HCV E1E2 are located at the top of each “strip” column, and can be used as reference for determining the position of each coevolving block within the E1E1 sequence. At the top of the figure is shown an enlarged linear representation of HCV E1E2, where the starting and ending residue positions of each protein and domain are indicated. E2 domains are highlighted by distinct colors (Green, BL; Red, central β-sandwich; Blue, front layer; Dark blue, CD81 BinL/CD81 binding loop; Light grey, central β-sandwich–back layer linker; Black dotted line, VR2/Variable Region 2; Grey dotted line, Stem; Black rectangle, Tmd/Transmembrane). (TIF) [file ppat.1006908.s018.tif]

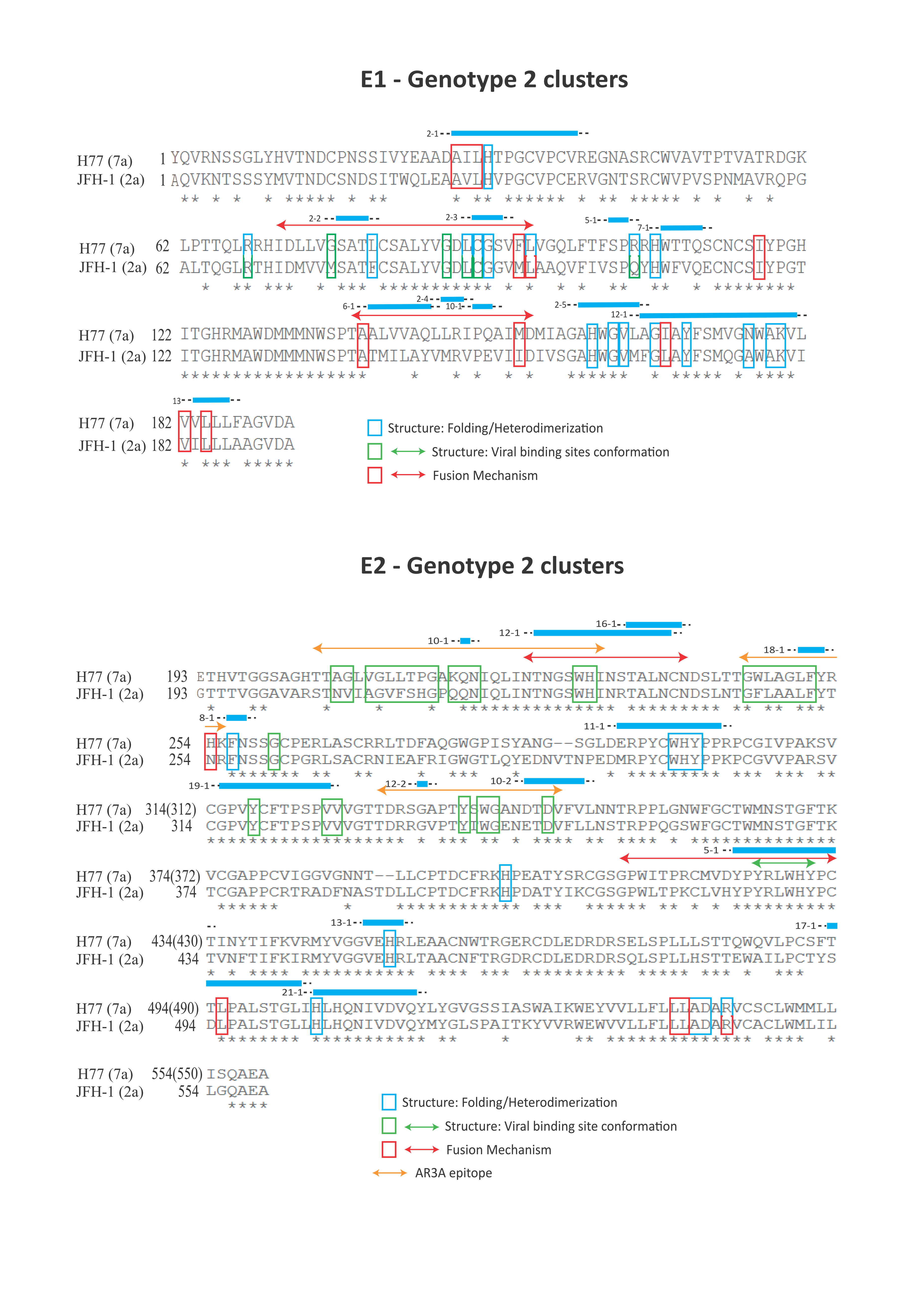

Supplement: S7 Fig — Genotype 2 cluster blocks are represented as blue horizontal bars positioned above the E1 and E2 H77 or JFH-1 aligned sequences. Each horizontal bar is numerated as follow: “Cluster number-Block N°”. Respective positions of these blocks are referenced in S8 Table. Residues (boxes) or domains (horizontal arrows) previously identified in the literature to have a function are indicated (see Online Methods for references). A color code, reported below the sequence alignment, links residues (boxes) or domains (arrow) to specific functions according to the literature: blue for folding/heterodimerization, green for binding and red for fusion. Multi-colored code boxes represent residues with two identified functions. In E2, the AR3A antibody epitope is shown as three orange arrows. The putative function determined for each cluster is summarized in S9 Table. The addition of one amino acid on the left and on the right of each block is considered to take into account potential structural adjustments after mutation that are not considered in BIS calculation. Cluster blocks positions are numbered according to JFH-1 gt2 (AB047639; S7 Table). (TIF) [file ppat.1006908.s019.tif]

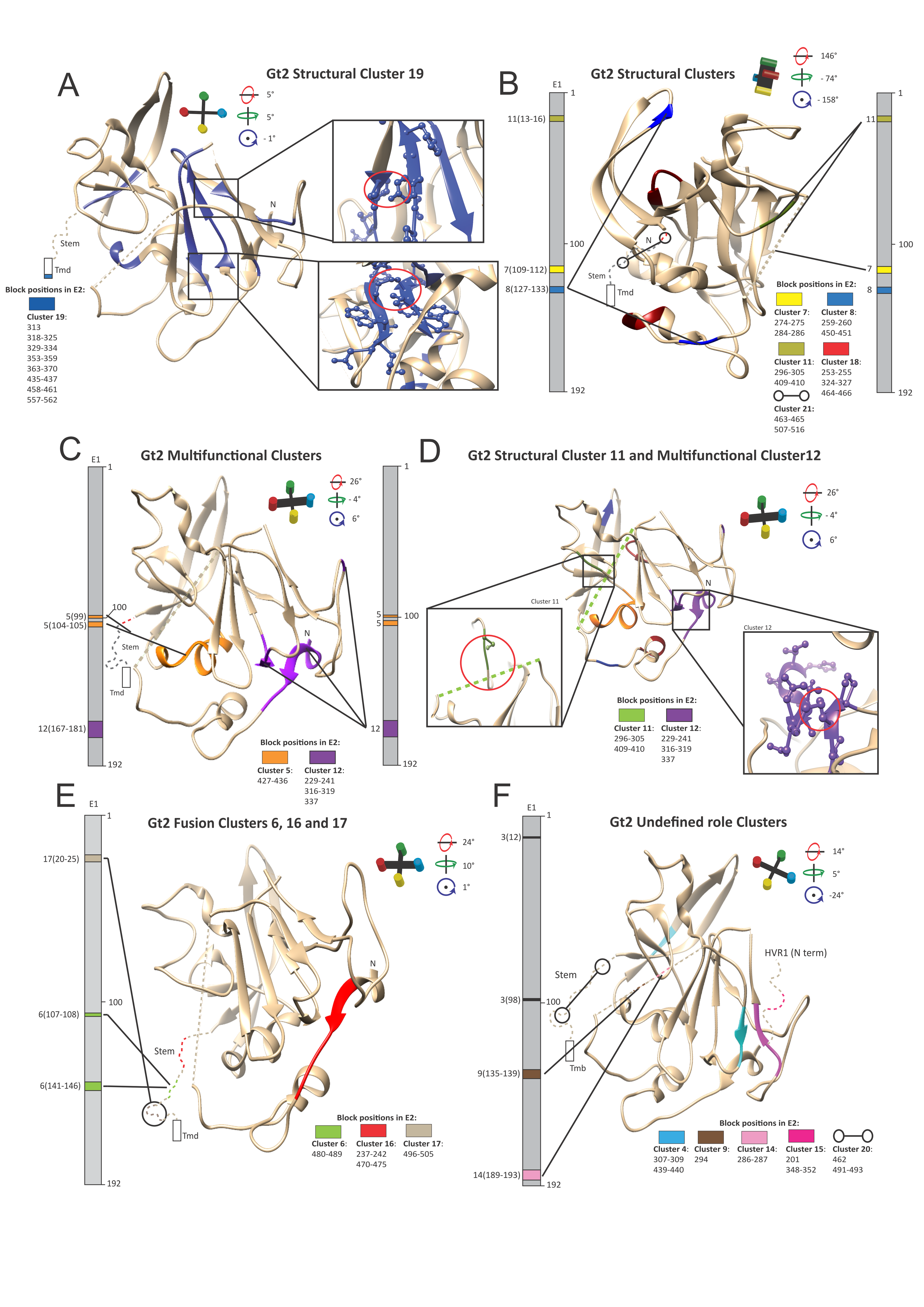

Supplement: S8 Fig — (A) Plot of the gt2 structural cluster 19 on a tridimensional view of the E2core structure (PDB 4MWF). Gt2 structural (B), multifunctional (C), structural and multifunction (D), fusion (E) and undefined (F) clusters were plotted both on a vertical linear representation of E1 and onto the E2 core structure. Each cluster is composed of blocks harboring a similar color, according to S6 Fig. The Stem region (Stem) is represented by a dotted line after the C-terminal part of the BL. The transmembrane domain (Tmd) is represented as a rectangle following the Stem region. In (B) and (C,E,F), bold lines link E1 and E2 blocks that coevolved. For each cluster, block positions in E1 (at the left of the linear structure) and E2 (below boxes whose color match the color of the corresponding cluster) are indicated. Inter-E1E2 interactions mediated by gt2 structural clusters 7, 8, 11 and intra-E2 interactions mediated by gt2 structural cluster 18 and 21 are represented in B, while multifunctional clusters 5 and 12 are represented in (C). Areas of close proximity between blocks in cluster 19 (A) and between blocks in cluster 11 (D) and 12 (D) are enlarged and highlighted by red circles. Panel E shows gt2 fusion clusters 6, 16 and 17. Panel F shows gt2 undefined role clusters 3, 4, 9, 14, 15 and 20. Rotation angles of the E2core structure are indicated. Viewing angle of E2core is indicated by a black cross (Reference: Fig 3B). HVR1, Hyper Variable Region 1, FL, Front Layer; BinL, CD81 Binding Loop; BL, Back Layer, βS, β-sandwich. (TIF) [file ppat.1006908.s020.tif]

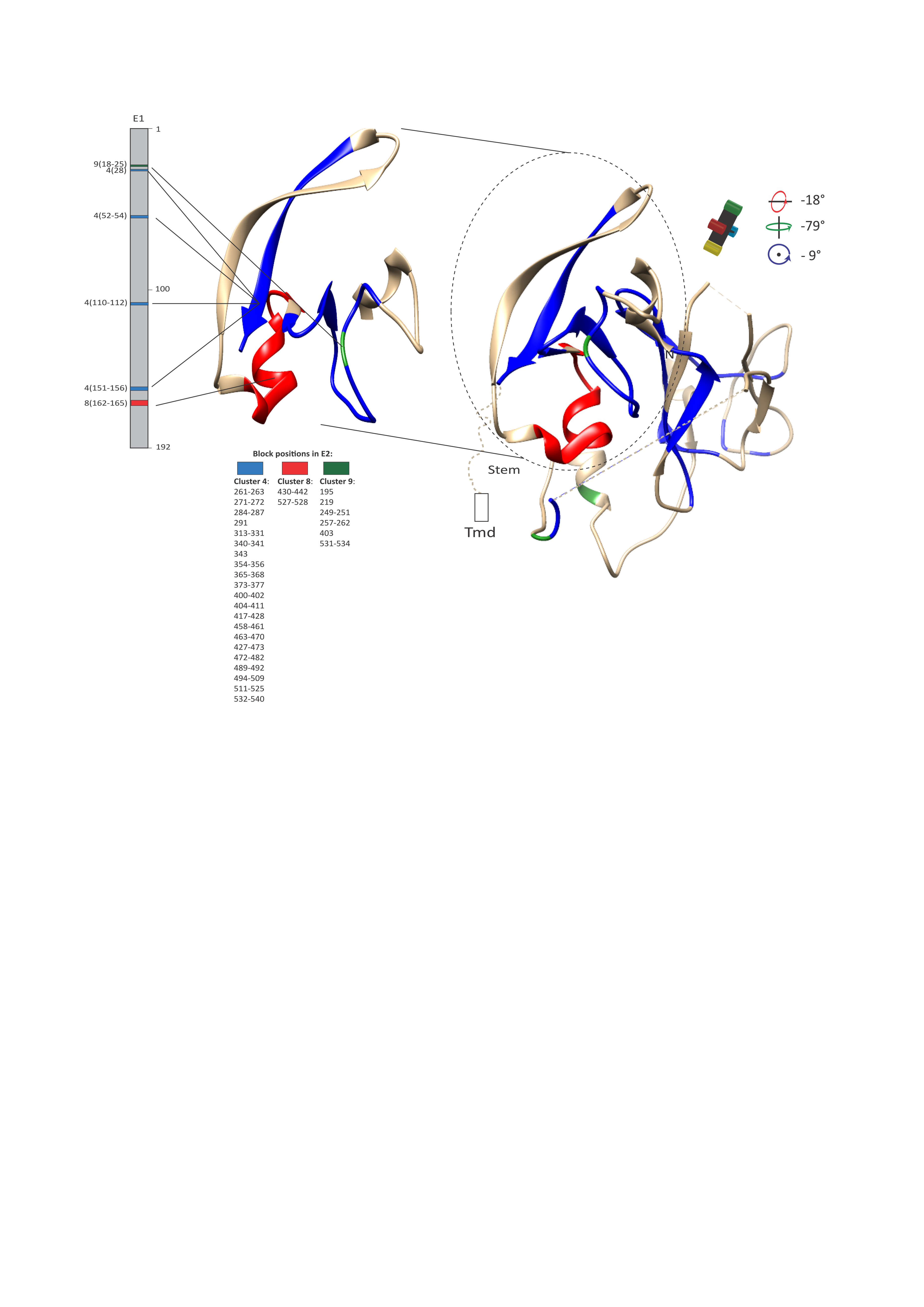

Supplement: S9 Fig — Genotype 3 cluster 4 (blue), cluster 8 (red) and cluster 9 (green) are plotted on a tridimensional view of the E2core structure (PDB 4MWF) and on a vertical linear representation of E1. Each cluster is composed of blocks harboring a similar color. The Stem region (Stem) is represented by a dotted line after the C-terminal part of the BL. The transmembrane domain (Tmd) is represented as a rectangle following the Stem region. The BL region is highlighted with a dotted circle on the E2core structure and enlarged alone at the right of the E1 linear representation. Bold lines link E1 and E2 blocks that coevolved. For each cluster, block positions in E1 (at the left of the linear structure) and E2 (below boxes whose color match the color of the corresponding cluster) are indicated. Rotation angles of the E2core structure are indicated. Viewing angle of E2core is indicated by a black cross (Reference: Fig 3B). Genotype 3 cluster positions are available through the following webpage: http://www.lcqb.upmc.fr/HCVenv/HCVenv.html/. (TIF) [file ppat.1006908.s021.tif]

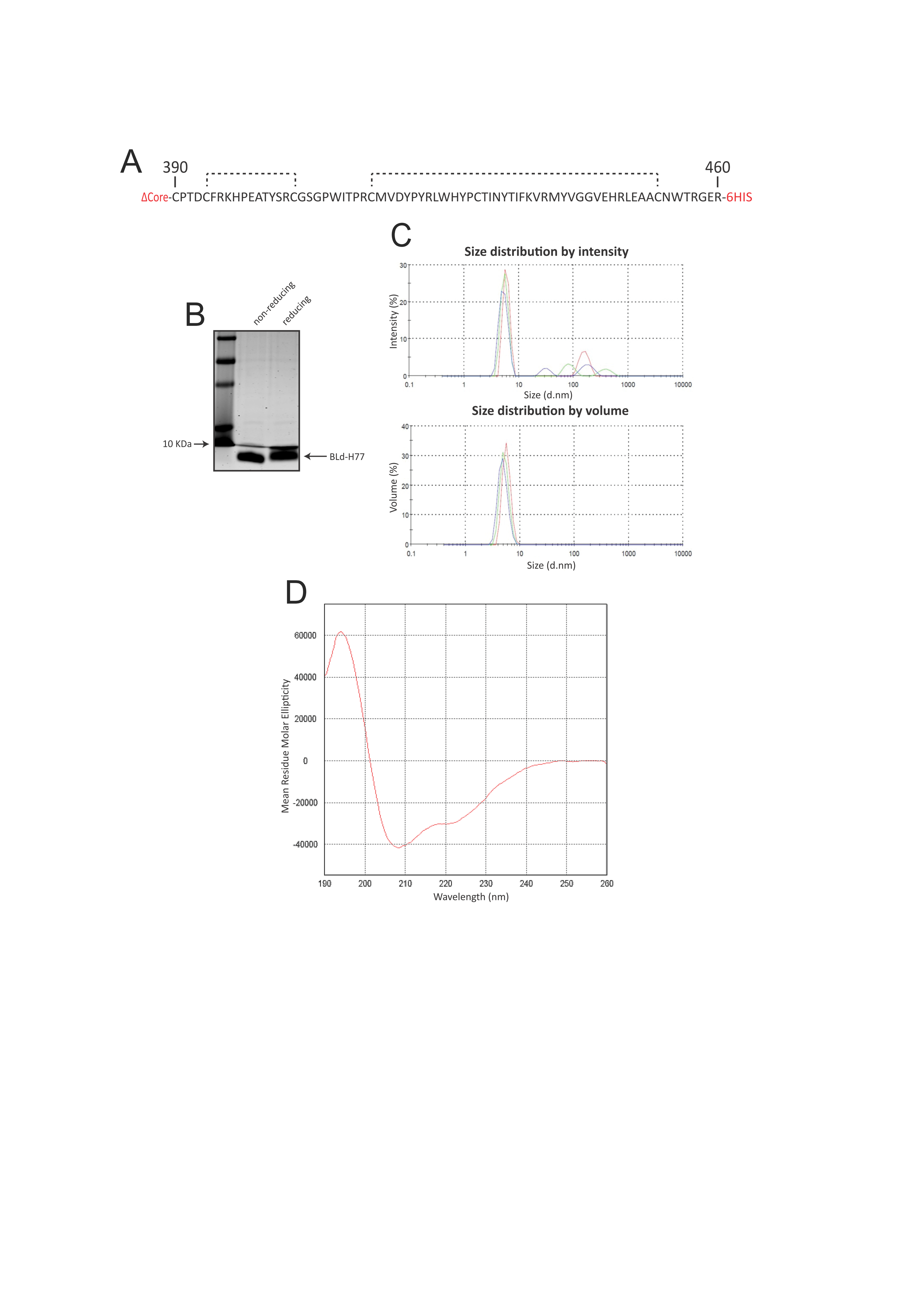

Supplement: S10 Fig — (A) Amino acid sequence of the soluble BLd-H77. Dotted lines represent internal disulfide bridges that might be involved in the functional folding of the soluble BLd-H77. (B) Detection of BLd-H77 in reducing and non-reducing condition following SDS-Page electrophoresis and coomassie blue staining. (C) Size distribution profiles as a function of the intensity and volume of soluble BLd-H77, by Dynamic Light Scattering. The graph shows the superposition of three successive measurements from 12 runs at each concentration, which are representative for at least three independent experiments. (D) Far UV circular dichroism spectrum of soluble BLd-H77 after purification by size exclusion chromatography. (TIF) [file ppat.1006908.s022.tif]

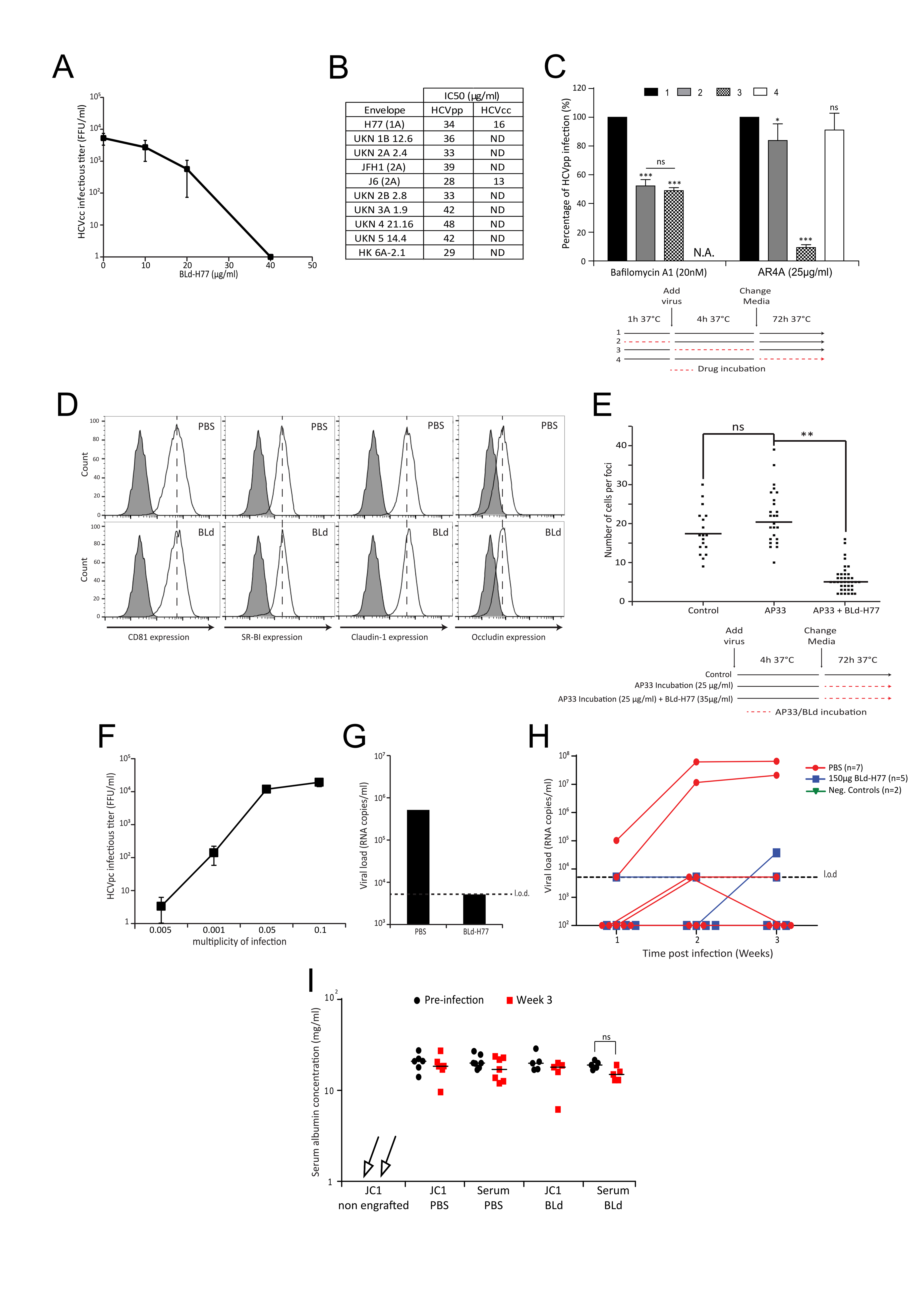

Supplement: S11 Fig — (A) Quantification of HCVcc infectious titers following dose-dependent neutralization of HCVcc H77/JFH-1 by BLd-H77. Four days following BLd-H77 dose-dependent neutralization of HCVcc H77/JFH-1 infection (see Fig 4F), Huh7.5 cell culture supernatants were harvested and used to infect naïve Huh7.5 cells. Four days post-secondary infection, viral titers were determined by NS5A immunostaining (mean ± SD; n = 3). (B) Average IC50 of BLd-H77 inhibition (μg/ml) for HCVpp or HCVcc particles of different genotypes or sub-types. ND, not determined. (C) Bafilomycin A1 and AR4A mediated-inhibition of HCV entry. Huh7.5 cells (cells) or HCVpp (HCVpp-H77, virus) were incubated 1h with Bafilomycin A1 (20nM) or AR4A (25μg/ml) prior infection (2), during the 4h infection (3) or following infection (4). As control, cells were incubated at each step with equivalent volume of PBS (1). Percentages of infection were calculated 72 post infection based on viral titer obtained from control conditions. (mean ± SD; n = 3). Statistical significances (*p<0.05, ***p<0.001, ns non-significant) were determined for each experimental condition versus control condition (100%). N.A., non applicable, as significant cell death was observed for this experimental condition. (D) HCV receptors expression is not impaired by BLd-H77. CD81, SR-BI, Claudin-1 and Occludin expression in Huh7.5 cells pre-incubated overnight with PBS (upper line) or BLd-H77 (50μg/ml; bottom line). Following PBS or BLd-H77 pre-incubation, cells were fixed, permeabilized (only for Occludin staining) and stained for the different HCV receptors with (white areas) or without (grey areas) receptor-specific antibody. Expression levels were then determined by flow cytometry. Data are representative of two independent experiments. (E) BLd-H77 inhibition of cell-to-cell transmission. After HCVcc H77/JFH-1 infection, cells were pre-incubated with PBS (control), with AP33 antibody alone (25μg/ml) or with AP33 mixed with BLd-H77 (35μg/ml). Aver [file ppat.1006908.s023.tif]

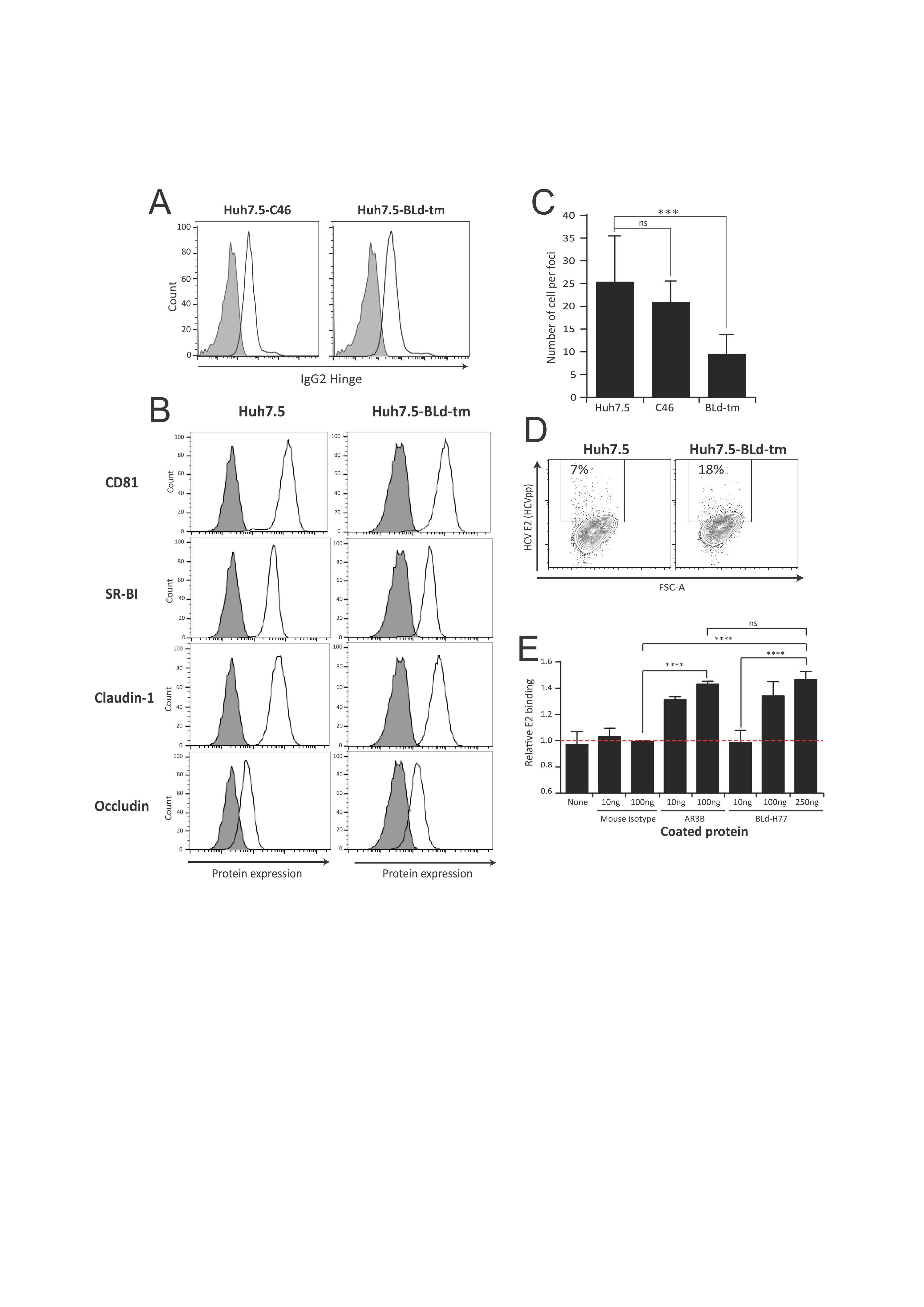

Supplement: S12 Fig — (A) Cell surface staining of C46 and BLd-tm following transduction of Huh7.5. Expression of C46 and BLd-tm (white) was measured by flow cytometry using an anti-hinge human IgG2 antibody and compared to the level of expression within non-transduced Huh7.5 (Grey). (B) CD81, SR-BI, Claudin-1 and Occludin expression within Huh7.5 (left column) and Huh7.5 expressing the transmembranous form of the BLd-H77 (Huh7.5-BLd-tm) (right column) cells. Cells were fixed, permeabilized (only for Occludin staining) and stained for the different HCV receptors with (white areas) or without (grey areas) receptor-specific antibody. Expression levels were then determined by flow cytometry. Data are representative of two independent experiments. (C) Effect of BLd-tm expression on cell-to-cell viral spread. Huh7.5, Huh7.5-C46, Huh7.5-BLd-tm cells were infected by H77/JFH-1 HCVcc. Four days post-infection, average number of cells per foci were determined for each cell type (mean ± SD; n = 3). ***p<0.001, ns non-significant. (D) Retention of HCVpp particles at the Huh7.5-BLd-tm cell surface. Huh7.5 (left) and Huh7.5-BLd-tm (right) were infected with H77 HCVpp particles. Presence of HCV E2 at the cell surface was quantified by flow cytometry using anti-E2 H53 antibody. Gating were set up on non-infected Huh7.5 (left) and Huh7.5-BLd-tm (right). Data are representative of two independent experiments. (E) Interaction between BLd-H77 and sE2 detected by ELISA. Different amounts of mouse IgG isotype, AR3B and BLd-H77 were coated overnight into 96-well plates. Coated peptides and antibodies were then incubated with 10ng of soluble E2 (sE2). After washing, soluble E2 was detected using the anti-E2 antibody 3/11 or a rat isotype IgG antibody. Following incubation with an anti-rat HRP antibody, 3/11 binding specificity to E2 was determined by calculating the ratio of O.D. between condition using 3/11 antibody and the rat isotype IgG antibody, for each coating condition (mean ± SD; n = 3). ****p<0.0001, [file ppat.1006908.s024.tif]

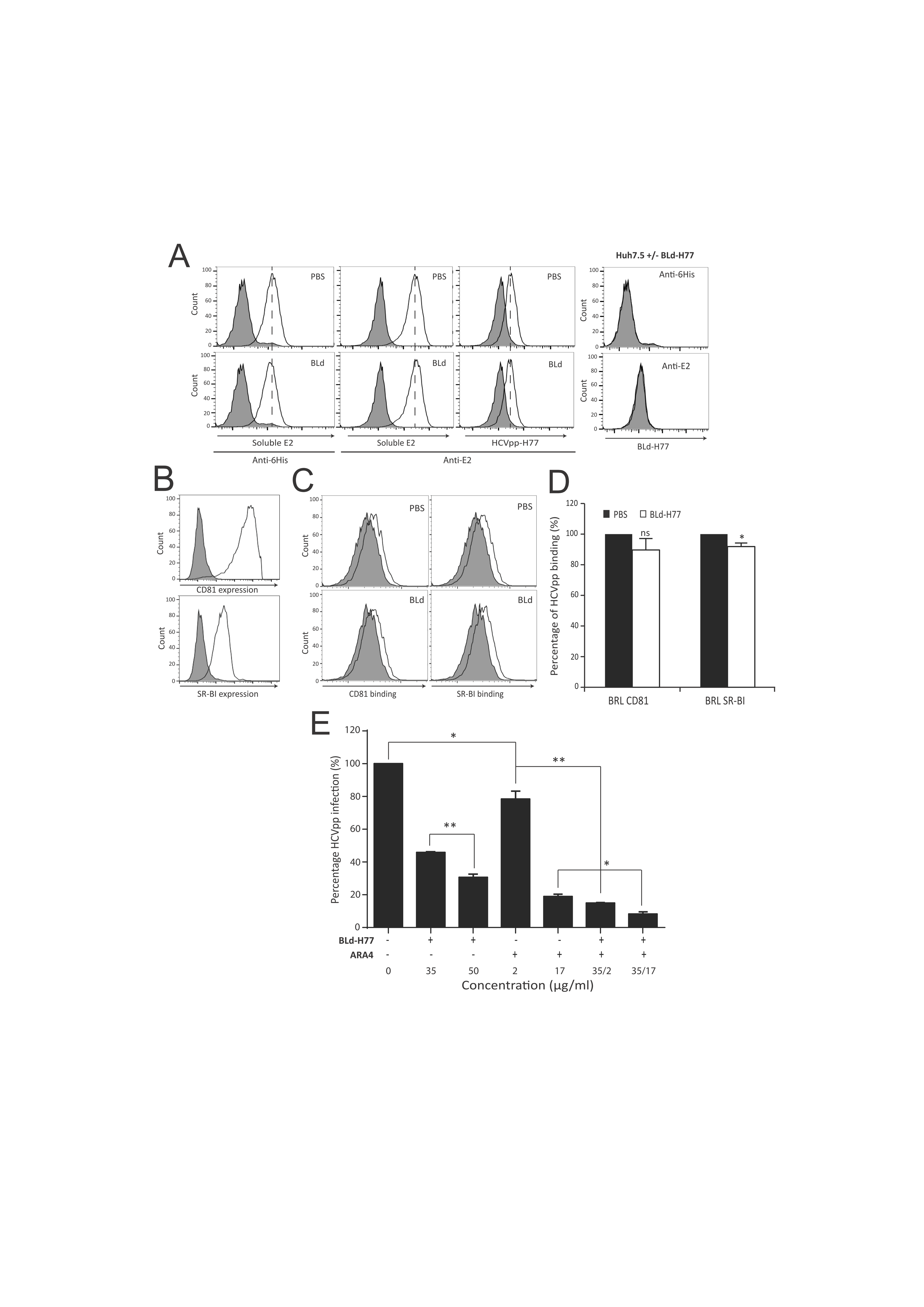

Supplement: S13 Fig — (A) BLd-H77 effect on E2 and HCVpp binding to Huh7.5 cells (Left). Huh7.5 cells, pre-incubated with PBS (top panels) or BLd-H77 (50μg/ml) (bottom panels), were mixed (white) or not (grey), at 37°C for 1h with soluble E2 or with concentrated H77-HCVpp. Bound soluble E2 or HCVpp were then stained with an anti-6his tag antibody or an anti-E2 antibody, respectively. Binding was then quantified by flow cytometry following incubation with appropriate APC antibody. Data are representative of two independent experiments. Ability of BLd-H77 to bind to Huh7.5 cell membrane (Right). BLd-H77 (50μg/ml, white) or PBS (grey) was incubated for 1h at 37°C with Huh7.5 cells. Absence of bound BLd-H77 at Huh7.5 cell surface was verified by flow cytometry following staining using an anti-6his antibody or an anti-E2 antibody as control. (B) Expression of CD81 and SR-BI at BRL cell surface and HCVpp binding. BRL cell lines were transduced by vectors encoding human CD81 or SR-BI (hSR-BI). After selection, naïve BRL (grey) or transduced-BRL (white) cells were stained by anti-CD81 or anti-hSR-BI and by the appropriate secondary APC antibody. Quantification of CD81 and hSR-BI expression was determined by flow cytometry. (C) BLd-H77 effect on E2 and HCVpp binding to CD81 and hSR-BI. Naïve BRL (grey) or HCV-receptor expressing BRL (white) cells were mixed with concentrated H77-HCVpp pre-incubated with PBS (top panels) or BLd-H77 (50μg/ml) (bottom panels). Bound HCVpp were then stained with an anti-E2 antibody and with an appropriate APC antibody. Binding was then quantified by flow cytometry following incubation. Data are representative of three independent experiments. (D) Relative quantification of HCVpp binding to BRL cells expressing exogenous hCD81 or hSR-BI. Binding of HCVpp is presented as percentage of binding, relatively to the binding efficiency of HCVpp pre-incubated with PBS. (mean ± SD; n = 3). Statistical significances (*p<0.05, ns non-significant) were determined for each experim [file ppat.1006908.s025.tif]

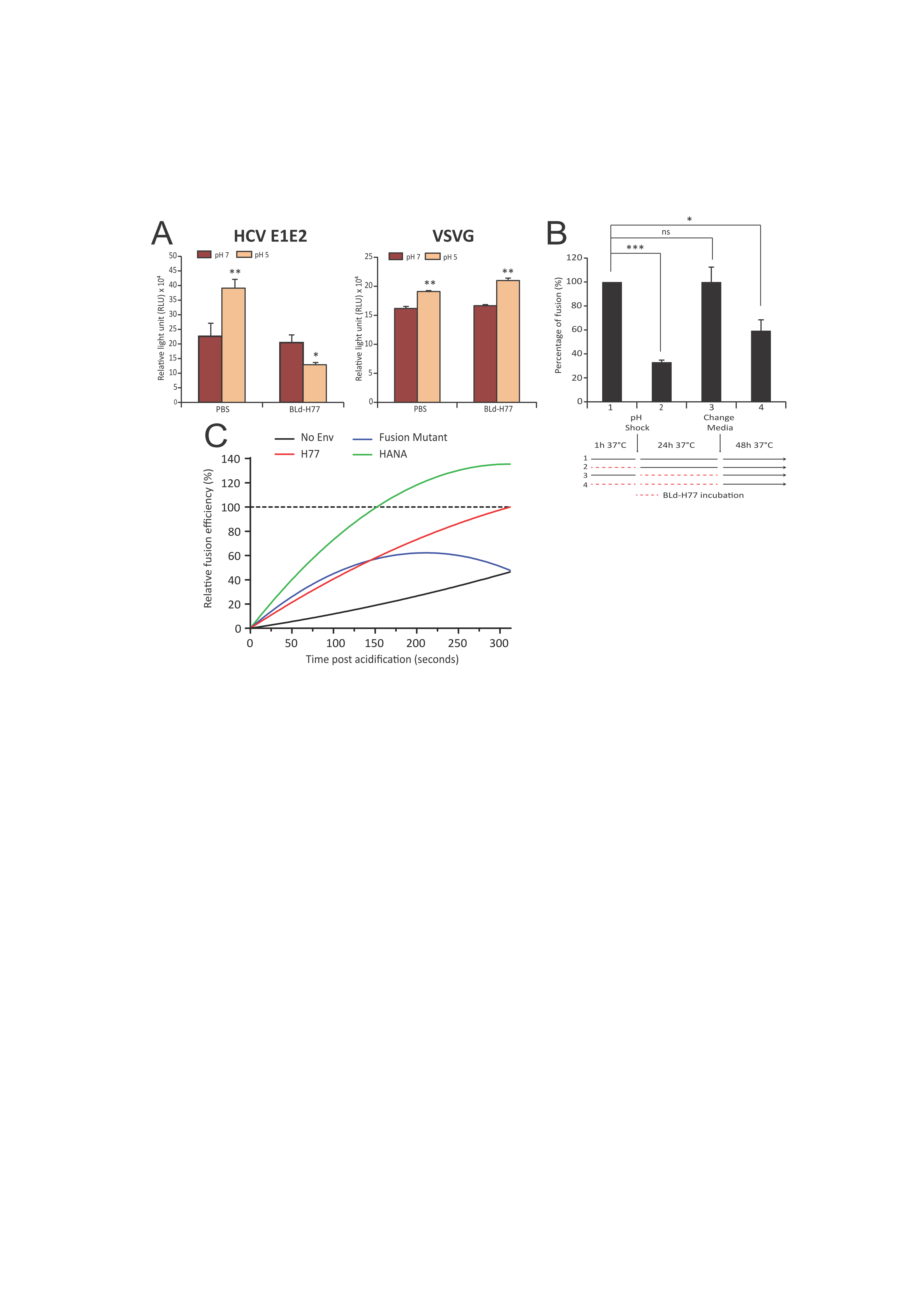

Supplement: S14 Fig — (A) LTRhiv-luciferase vector transduced 293T cells expressing H77 HCV (left panel) or VSV (right panel) envelope glycoproteins were co-cultured with Tat-expressing Huh7.5 cells. Co-culture were pre-incubated for 1h with 50μg/ml of BLd-H77 or PBS, washed and incubated for 3 min with a pH7 (red) or pH5 (orange) buffer. Luciferase activities were determined 72h post-exposure. Results are presented in relative light units (RLU) for each experimental condition (mean ± SD; n = 3). *p<0.05, **p<0.01. Statistical significances represent the significant differences between pH5 and pH7 condition, for each viral envelope and treatment (PBS or BLd-H77). (B) Inhibitory effect of the BLd-H77 prior and following pH shock. Co-cultures were either pre-incubated with BLd-H77 (50μg/ml, 2) for 1h prior pH shock (pH5), for 24h following pH shock (50μg/ml, 3) or both (4). As a control, co-cultures were pre-incubated with PBS for 1h prior pH shock, followed by an additional 24h incubation (1). 24h later, cells were washed and incubated for 48h. Relative fusion efficiency at pH5 between control (pre- and post-treated by PBS) and the different BLd-H77 incubation periods are indicated (mean ± SD; n = 3). Statistical significances (*p<0.05, ***p<0.001, ns non-significant) were determined for each experimental condition versus control condition (100%). (C) Specificity of HCVpp-liposome fusion assay. No enveloped HCVpp, H77 HCVpp, fusion mutant E1E2 HCVpp particles and HANApp particles (Retroviral particles pseudotyped with Influenza Hemagglutinin-Neuraminidase) were mixed with R18-labelled liposomes. Dequenching of R18 was quantified following sample acidification (pH5) over 5 minutes (300 seconds). Data are represented as non-linear polynomial fitted curves and display the evolution of the fusion rate from the time of acidification, relatively to the HCVpp-H77 fusion rate which has been normalized at 100% at 300s post acidification. Curves are representative of three independent experiments. [file ppat.1006908.s026.tif]

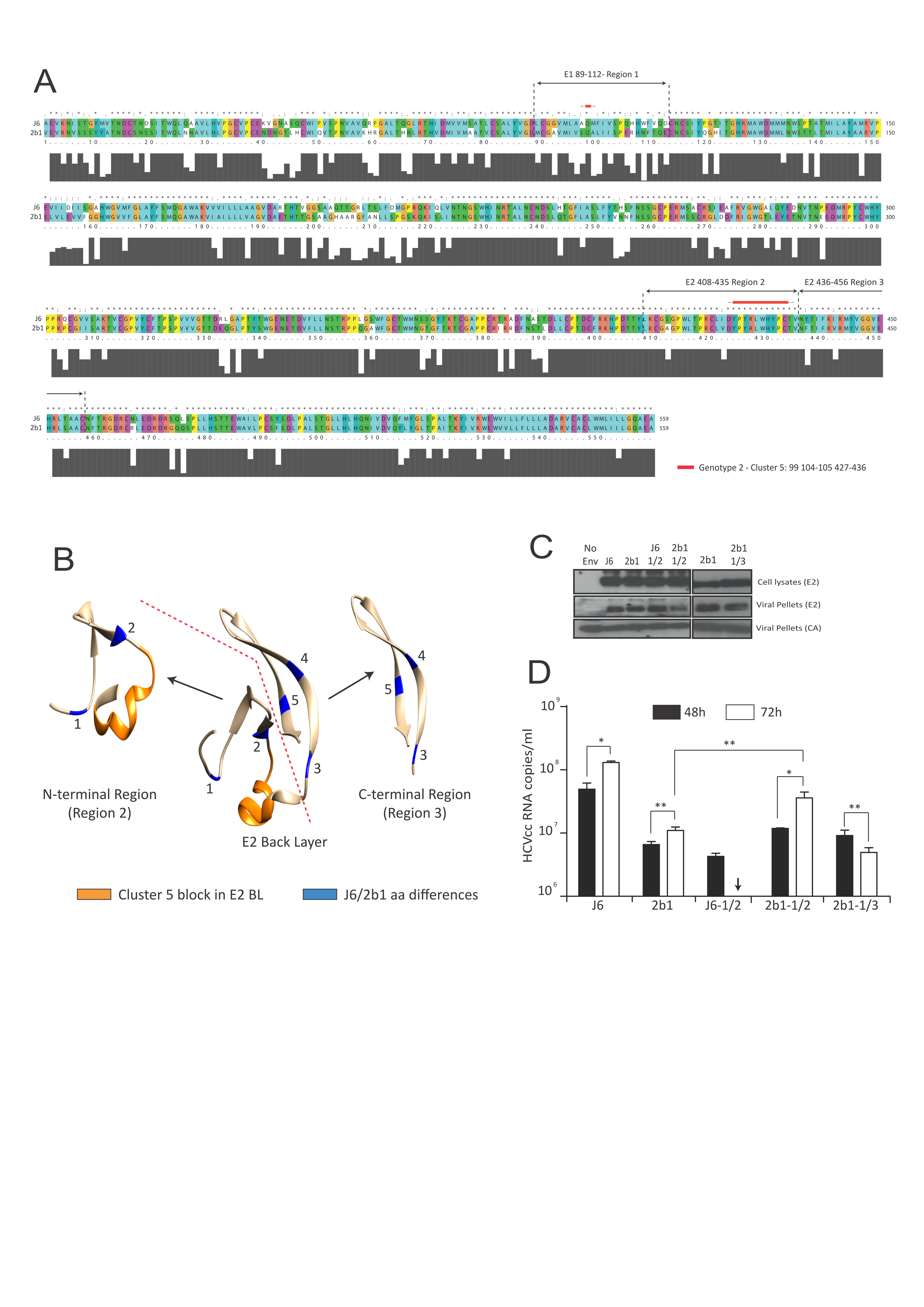

Supplement: S15 Fig — (A) Protein sequence alignment of E1E2 J6 and 2b1. Level of conservation for each amino acid position is indicated. Position of the coevolving blocks that belong to gt2 fusion cluster 5 are indicated by red rectangles. Position of the interchanged amino acid regions between J6 and 2b1 for the construction of the J6/2b1 chimeric envelope are shown (Region 1, 2 and 3). (B) Structural location of the E2 gt2 cluster 5 block (orange) and of the five J6/2b1 amino acids differences (blue, 1 to 5) within the E2 BL. A dotted line symbolizes the rational experimental dissection of the BL into two distinct sub-domains, noted region 2 (non-β sheet region) and region 3 (β sheet region), based on the distinct rearrangement of these two sub-domains. The distinct structural contexts of the mutation 1–2 (region 2, left) and of the mutation 3–5 (region 3, right) are highlighted. (C) J6/2b1 E2 chimera expression and incorporation onto HCVpp. Expression in transfected 293T cells (Cell lysates) and incorporation onto concentrated pseudoparticles (Viral Pellets) of E2 from J6, 2b1 and J6/2b1 chimera. Detection of E2 on pseudoparticles harboring no envelope glycoproteins was used as negative control. MLV-Capsid (CA) was detected to control equivalent HCVpp production between chimera. (D) Quantification of HCV viral RNA in the supernatant of Huh7.5 cell electroporated with viral RNA coding for HCVcc harboring different J6/2b1 envelope chimera. Cell culture supernatant were harvested at 48h and 72h post electroporation and viral RNA copy numbers per ml were determined by RT-qPCR for each viral strain (mean ± SD; n = 4). *p<0.05, **p<0.01. (TIF) [file ppat.1006908.s027.tif]

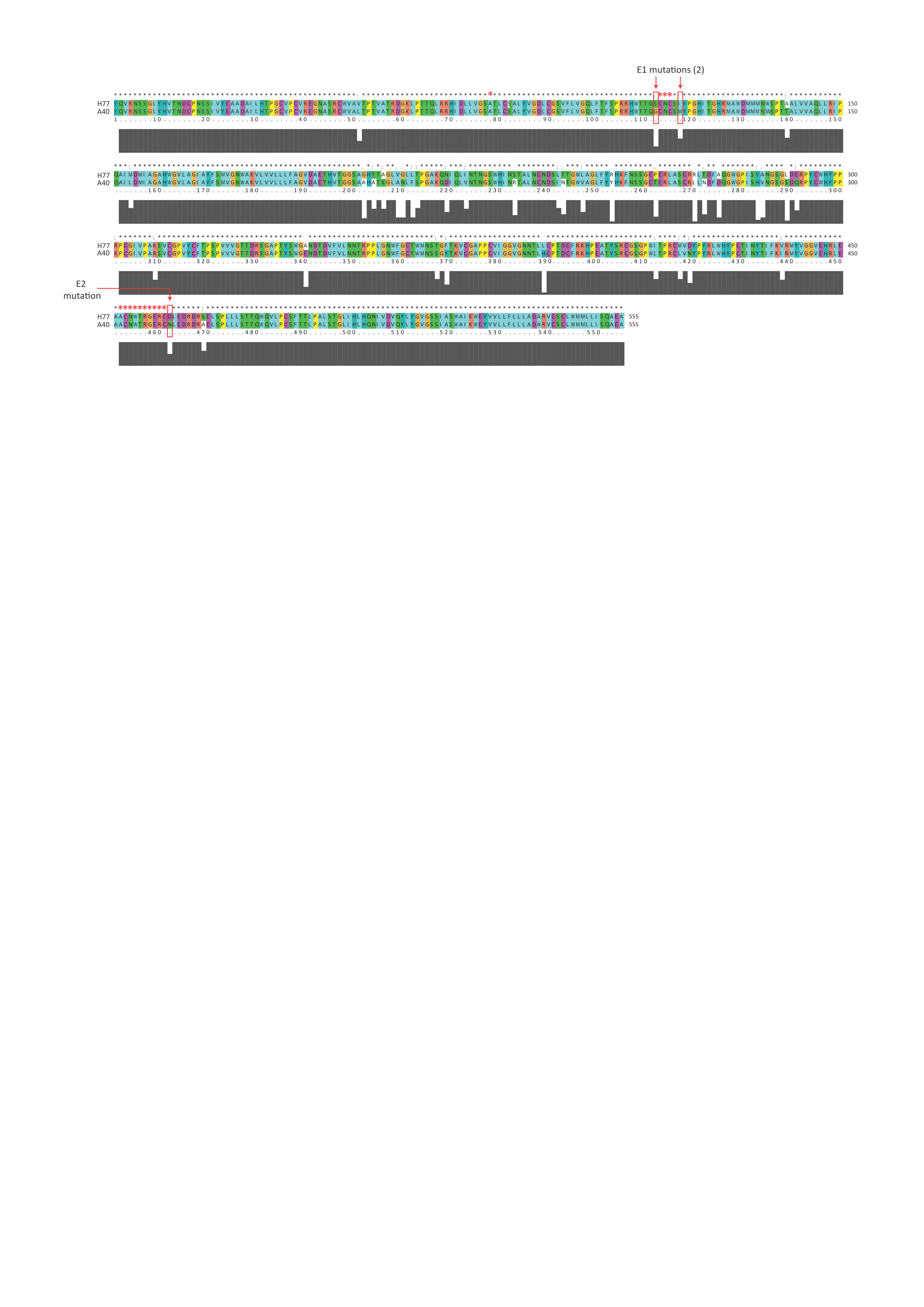

Supplement: S16 Fig — Protein sequence alignment of E1E2 H77 and A40. Level of conservation for each amino acid position is indicated. Position of the E1 and E2 block of interest belonging to the gt1a cluster 5 are indicated by red asterisks. Position of the three H77 amino acid residues that will be replaced by A40 residues for the construction of H77/A40 chimeric envelopes are indicated by red rectangles (two in E1 and one in E2). (TIF) [file ppat.1006908.s028.tif]
